# Not in My Backyard: Public Perceptions of Wildlife and ‘Pest Control’ in and around UK Homes, and Local Authority ‘Pest Control’

**DOI:** 10.3390/ani10020222

**Published:** 2020-01-30

**Authors:** Sandra E. Baker, Stephanie A. Maw, Paul J. Johnson, David W. Macdonald

**Affiliations:** 1 Wildlife Conservation Research Unit, Department of Zoology, University of Oxford, Recanati-Kaplan Centre, Tubney House, Abingdon Road, Tubney, Abingdon OX13 5QL, UK; paul.johnson@zoo.ox.ac.uk (P.J.J.); david.macdonald@zoo.ox.ac.uk (D.W.M.); 2 Campaigns Consultant to Humane Society International UK, 5 Underwood St, Hoxton, London N1 7LY, UK; smaw@hsi.org

**Keywords:** attitudes, mouse, pest, pest control, rat, tolerance, vermin, wasp, wildlife, wildlife management

## Abstract

**Simple Summary:**

Wildlife causes ‘pest’ problems globally. Controlling wildlife involves killing and harming the welfare of many animals. We examined public perceptions of 10 wildlife species and wildlife management, in and around UK homes, as well as council ‘pest control’ services, to identify ethical, welfare-friendly ways to reduce wildlife problems. Most people had never had problems with each of the 10 species, and problems with some species were largely tolerated. Wasps, mice, and rats were the most frequently problematic species, the least tolerated and those for which councils most often offered ‘pest control’ services. People preferred Do-It-Yourself pest control over professional control, except for with wasps. They wanted control to be quick, lasting, and safe for people and non-target animals. Where people accepted the killing of wildlife, they still considered animal welfare important. Factors influencing pest status were complicated, while factors influencing people’s demand for pest control were fewer, simpler, and species-specific. Council pest control provision increased over the four years studied, but only half of councils offered advice on preventing/deterring wildlife; this advice was patchy and variable in quality. More effort should be put into preventing/deterring rather than controlling wildlife problems. Councils should provide standardised, informative advice on prevention/deterrence and prevention/deterrence services.

**Abstract:**

Human–wildlife conflict occurs globally. Attempts to control ‘pest’ wildlife involve killing and harming the welfare of animals on a vast scale. We examined public perceptions of 10 wildlife species/groups and wildlife management, in and around UK homes, and public authority ‘pest control’ provision, in an effort to identify ethical, welfare-friendly ways to reduce conflict. Most people reported never having problems with each of the 10 species, and reported problems for some species were largely tolerated. Wasps, mice, and rats were the most frequently problematic species, the least tolerated, and those for which local authorities most often offered pest control services. Do-It-Yourself pest control was preferred over professional control, except for with wasps. People wanted control to be quick, lasting, and safe for people and non-target animals. Where people accepted lethal control, they were nevertheless concerned for animal welfare. Drivers of pest status were complex, while drivers of demand for control were fewer and species-specific. Local authority pest control provision increased over the four years studied, but only half of councils offered advice on preventing/deterring wildlife; this advice was patchy and variable in quality. Greater focus is required on preventing/deterring rather than controlling wildlife problems. Councils should provide standardised, comprehensive advice on prevention/deterrence and prevention/deterrence services.

## 1. Introduction

Human–wildlife conflict is an age-old and worldwide problem. Such conflict encompasses a wide diversity of issues and involves many different types of organism from plants to large carnivores [1]. In parts of the world, wildlife conflict frequently threatens human lives, either directly—for example, where large carnivores are involved—or indirectly, such as where livelihoods are affected by crop loss [2,3]. In the UK, problems of that severity occur rarely, but conflict is nonetheless widespread, particularly where agriculture is concerned. However, conflict with wildlife also affects people in an around their homes, and how humans react to this may have considerable implications for animal welfare.

A number of species are commonly regarded as problematic in and around UK homes and often acquire the label of ‘pest’ or ‘vermin’. For example, brown rats (*Rattus norvegicus*) and house mice (*Mus domesticus*) pose human and animal health risks and damage property and food [4,5]. European mole (*Talpa europaea*) activity in gardens often results in what is considered ‘damage’ [6]. Urban gulls (*Larus argentatus* and *L. fuscus*) are blamed for noise, mess, and aggression [7]. Wasps (e.g., *Vespula vulgaris*) cause painful stings, and (rarely) death through anaphylactic shock [8,9]. Grey squirrels (*Sciurus carolinensis*) damage trees, lawns, and property and take food left out for wild birds [10]. Feral pigeons (*Columbia livia*) foul pavements and buildings, and they are blamed for transmitting allergens, disease, and ectoparasites [11]. Red foxes (*Vulpes vulpes*), Eurasian badgers (*Meles meles*), and European rabbits (*Oryctolagus cuniculus*) may dig up lawns, badgers sometimes excavate and defecate in latrine pits, rabbits eat plants, and foxes may take or disturb poorly protected poultry, food waste, or domestic refuse kept outside. 

Not all people are equally vulnerable to pests. There is little information on the rate of wildlife conflict, or its causes, in and around UK homes, but useful data exist on rat and mouse problems. The prevalence of Norway rats and house mice in and around English domestic homes was estimated using a systematic survey between 1996 and 2010 [4,5]. Over the 15-year period, mice were found to be present in 1.7–2.65% of dwellings, while rats were present inside 0.28–0.46% of properties and outside 2.09–3.92% of dwellings [4]. Greater rodent prevalence was associated with older dwellings, homes in rural areas, those with pets or other animals kept in the garden, vacant or derelict properties, and homes with litter, drainage faults, or unkempt gardens [4,5]. Not only do rats and mice cause the most substantial wildlife conflict issues in and around human dwellings in the UK [4], but they are also among the most significant, prolific, and widespread urban pests in the world [12]. As well as the research on rats and mice, a 2007 public survey found that 13% of householders had experienced mole activity in their gardens, and most considered this to be damage [6].

The scale, or cause, of the presence of other problematic wildlife species in UK homes and gardens has not been studied. Some research has examined farmers’ perceptions of pests [13,14,15] and compared perceptions among farmers and the urban public, and among urban people with urban and rural backgrounds [16]. However, otherwise, there has been no research to our knowledge on public perceptions of problems with wildlife in and around people’s homes, nor how (if at all) people respond to these. 

Traditionally, where people have come into conflict with wildlife, they have attempted to ‘control’ the species concerned, usually by killing individuals of the species thought to be responsible. Efforts to control them can affect the welfare of vast numbers of animals. In the mid-16th century, partly prompted by an increasing human population, a series of vermin elimination campaigns began, and financial incentives for killing a wide range of species were introduced [17]. This killing continues in the 21st century, and while non-lethal wildlife management methods are increasingly used, many millions of rats and mice are killed each year as pests [18]. However, lethal control may not always be effective [19], particularly where territorial animals are concerned. The removal of territorial animals may produce only local and short-term reductions in damage as a result of density-dependent compensations and swift replacement by conspecifics [20,21]. In territorial species, effective culling should be targeted at breeding females [22,23], as animals killed non-selectively over the autumn or winter are likely to be dispersing juveniles [22] from the ‘doomed surplus’ [24] and destined to die anyway when resources are limited. Lethal control of territorial animals can sometimes be counter-productive: for example, the removal of badgers may cause perturbation of their social systems, such that unculled badgers roam further, increasing the spread of disease [25,26]. Finally, there is a risk that, despite great efforts at population reduction or eradication, a single remaining animal can cause a large amount of damage [27]. Lethal control can also be time-consuming [28] and therefore expensive [29].

Historically, there has been little regard for the welfare of animals regarded as ‘pests’ [30], and it is noteworthy that interactions with small problematic animals are often labelled ‘pest control’, while those with large animals are termed ‘human–wildlife conflict’ [31]. Welfare regard has been particularly poor for high-profile pest species, such as rats and mice, which can often be killed legally using lower-welfare methods than other species. Indeed, in their review of the humaneness of rodent pest control, Mason and Littin [18] highlighted “remarkable paradoxes in the way society treats different classes of animal”. For example, under EU law, despite the fact that anticoagulant rodenticides (ACRs) fail safety standards that would normally preclude them from approval, ACRs are currently approved (by way of an exception according to Article 5(2) of Regulation (EU) No 528/2012), because only limited viable alternatives for ‘controlling’ rats and mice are considered to be available. Meanwhile, under UK law, some unregulated spring traps may be used only for rats, mice, and moles [32]. Glue boards (sticky boards used for trapping rats and mice) can be used for trapping rats and mice, phosphine gas may be used for poisoning moles, rabbits, and rats, and neck snares can be used for capturing rabbits and foxes. Each of these methods, used in lethal control, is associated with conspicuous welfare impacts. However, it is important to recognise that while it is often assumed that non-lethal wildlife management is ‘humane’ or ‘welfare-friendly’, this is not always the case (e.g., [33,34,35]).

Recognition of the welfare needs of wild animals is slowly increasing. For example, in the UK, the Wild Mammals (Protection) Act (1996), the Hunting Act (2004), and the Animal Welfare Act (2006) have been introduced. The welfare impacts of some wildlife management methods are better understood (e.g., [34,36,37]) and an international collaboration published seven consensus principles for ethical wildlife control [38]. 

People’s relationships with animals are complex and riddled with irrationality [39]. People in the UK are no different where wildlife is concerned [40]. On one hand, people demonstrate deep concern for some wildlife species under threat of lethal management; for example, a proposed cull of ‘invasive’ hedgehogs on Uist off the west coast of Scotland was abandoned after widespread hostility from animal lovers and conservationists [41,42]. Also, thousands of people marched against the UK government badger cull policy, which is intended to reduce bovine tuberculosis (bTB) in domestic cattle [43]. Yet, a wide variety of amateur ‘pest control’ products, both lethal and non-lethal, are widely available. 

Where people are uncertain about appropriate action to take when encountering a wildlife nuisance, they may seek advice or help with preventing or managing problems from their local council. While not legally obliged to provide pest management services, many councils in the UK offer such services to residents and local businesses, and these are often at least partly funded by the public purse. However, we are not aware of any published data on the public pest control services provided by UK councils, for example, what services are offered, for what species, or by which councils. Such information would help to determine whether council pest control services are working to reduce wildlife problems in the long-term.

Our aim is to provide a comprehensive account of how a diverse sample of the public answered questions concerning wildlife species and their control in and around homes in the UK. Here, we investigated UK pest status and control from two overlapping perspectives.

First, we conducted a nationwide UK public survey about perceptions of 10 species/animal groups commonly found in UK homes and gardens. The main aims of the survey were:

(1) To examine public attitudes, experiences, and beliefs about the species, their perceived problematic traits, whether people attempt to control perceived problems, and what factors are important in choosing pest control services and products;
(2) To examine the factors driving public attitudes, perceived pest status, and demand for pest control. A range of factors could be involved. For example, attitudes might be influenced by a person’s age or whether or not they own their home. Experience of conflict with wildlife might depend on whether a person lives in a town or the countryside, the age of their home, and whether or not they enjoy gardening or keep poultry. Demand for pest control may be linked with the traits people associate with different species, e.g., whether they think the species spreads disease or that its numbers are out of control.

Second, we quantified the public pest control services provided by UK local authorities, using:

(1) A desk study of council websites to identify which councils offer any sort of pest-related information or pest control services;
(2) A study of those councils offering pest control services, using data gathered through Freedom of Information requests. Our aim was to gain a clearer picture of the scale and modus of local authority pest control services, including their scope and financial cost.

We aimed to explore the relationship between the UK public and commensal wildlife to illuminate whether the scale of action against pest species, and its welfare implications, could be mitigated by more efficient preventative action. Is the welfare impact of pest control action proportionate to the real effects? Do the pest control services offered by local authorities currently meet the perceived needs of the public? Lastly, what changes could conceivably be made in the interests of both welfare and conflict mitigation?

## 2. Materials and Methods 

### 2.1. Ethical Review

The YouGov^®^ survey work included in this study has been granted ethical approval by the Social Sciences and Humanities Interdivisional Research Ethics Committee (IDREC) at the University of Oxford, with Research Ethics Approval (CUREC 1A) number R53946/001. 

### 2.2. YouGov^®^ Survey

A survey was designed to assess public perceptions of wildlife in UK homes or gardens, including badgers, foxes, moles, mice, pigeons, rabbits, rats, gulls, grey squirrels, and wasps. These species/animal groups are commonly considered as problematic. The purpose of the survey was (1) to examine patterns in public attitudes, experiences, and beliefs about the 10 species/animal groups, the problems that the species/animal groups may be associated with, whether people try to control these problems, and what factors are important in choosing pest control services and products; and (2) to examine the factors driving public attitudes towards the species, perceived pest status, and public demand for pest control. We also investigated where people would seek help with control, who they feel should be responsible for ‘controlling’ infestations, and who should pay for this. See Supplementary Materials (Document S1) for a copy of the questionnaire survey.

The survey was conducted online in December 2017 by the UK public opinion polling company YouGov^®^ (https://yougov.co.uk/). YouGov^®^ surveyed 2000 people across the UK, who were balanced for age interlocked with gender, social grade, and UK Government Office Region (GOR). The 12 GORs were allocated to six larger ‘geographical areas’ for analytical purposes (Supplementary Materials Table S1).

The species and animal groups that are the focus of this survey comprised five mammal species (Eurasian badger (*Meles meles*), red fox (*Vulpes vulpes*), European mole (*Talpa Europaea*), European rabbit (*Oryctolagus cuniculus*), and grey squirrel (*Sciurus carolinensis*), two mammal groups (rats, including brown rats (*Rattus norvegicus*) and black rats (*Rattus rattus*), and mice, including house mice (*Mus musculus*) and wood mice (*Apodemus sylvaticus*)), two bird groups (pigeons, including feral pigeons (*Columba livia domestica*), wood pigeons (*C. palumbus*), etc.), and gulls, including lesser black-backed gulls (*Larus fuscus*), herring gulls (*L. argentatus*), etc.) and an invertebrate group (wasps, including the common wasp (*Vespula vulgaris*), European hornet (*Vespa crabro*), etc.). These animals are generally widely spread throughout the UK, although moles are absent from Northern Ireland [44] and grey squirrels are absent from some parts of the UK, e.g., parts of Scotland, parts of Northern Ireland, parts of northern England, the Isle of Wight and parts of Anglesey, etc. [45]. Black rats are now rarely found in the UK, but they are sometimes found in seaport towns. Throughout this report, we refer to the species and animal groups as ‘species’.

We used the R statistics package [46] for statistical analysis. We explored correlations among attitudes towards species and whether or not people had experienced problems with species personally, whether they believed that species cause problems for people generally, and whether people experiencing problems tolerated them. Then, we modelled potential drivers of (a) attitudes; (b) perceived pest status; and (c) public demand for pest control for each of the 10 species. We used the R ‘glm’ procedure for binary responses and Cumulative Links Models (CLM) fitted with the ‘ordinal’ R package [47] for Likert scale responses. Three responses concerning perceived pest status were modelled: (1) personal experience of problems with each species; (2) beliefs about whether each species caused problems for people generally in the UK (both ‘yes/no’ binary responses); and (3) use of negative language to describe each species (a Likert scale ranging through ‘not pest or vermin’, ‘pest’, ‘pest and vermin’, and ‘vermin’). One measure of demand for pest control was modelled: whether people that had reported prior experience of problems with a species had attempted control in the past (a ‘yes/no’ response). Attitudinal data were collected on a 5-point ‘Likert’ scale.

Predictors used in all models (except the model for negative language) described participants’ demography, homes, behaviours, and their perceptions concerning species and their traits. First, we created separate sub-models for each dependent variable for each species using predictors from only one category of predictor (e.g., there was a sub-model using demography predictors to model attitudes towards rats, a sub-model using home predictors to model attitudes towards rats, and so on). Then, we combined significant predictors from each sub-model (e.g., the demography, home, behaviour, perceptions, and perceived traits sub-models for attitudes towards rats) to create a final model for each species (e.g., predicting attitudes towards rats). For the models predicting the use of negative language to describe a species, we used only perceived species traits as predictors in an effort to identify whether certain types of traits led to the use of negative language. AICc (Akaike Information Criterion) values were used to identify the best performing model in each case. Effect sizes were visualised using the R ‘effects’ package [48]. Models including predictors with moderate levels of collinearity were retained only if each predictor added to a prediction based on the other predictor. In some cases, models failed to converge. Convergence failure can arise for a number of reasons. Here, we suspect that some models failed owing to a low response rate generating models too complex for the data set, or where responses were homogenous.

### 2.3. Council Freedom of Information (FOI) Requests

In order to determine the scale and mode of public pest control services provided by local authorities in the UK, as well as their scope and financial cost, we used a two-stage approach. First, we made a preliminary investigation into the availability of local authority pest control services, such as visits and treatments, and pest advice, by gathering information made publicly available on council websites across the UK in 2017/18. All 417 principal local authorities operating across the country were included. We established that the provision of public pest control services (for residents and local businesses) typically falls within the remit of unitary and district councils; 309 of 390 such councils were identified as providing some form of public pest control service within their jurisdiction.

Using these preliminary findings, requests were submitted under the Freedom of Information (FOI) Act 2000 to the 309 councils in order to gather information regarding service types, budgets, expenditure, and revenue as well as the number of requests received for pest control treatments for eight ‘species’ (species/animal groups); these were the only species/animal groups treated by councils (other than bedbugs, cockroaches, and ants), according to our preliminary investigation. Councils were requested to provide annual data for the most recent four-year financial period, from 2013/14 until 2016/17. 

We explored geographic and temporal patterns in the proportions of councils providing different service types. Similarly, we explored patterns in council expenditure and revenue related to pest control. We looked at councils’ net annual losses and the number of treatment requests per 1000 households.

## 3. Results

### 3.1. YouGov^®^ Survey

#### 3.1.1. Demographics

The 2000 survey participants were aged 18–93 and evenly split between men (48%) and women (52%). Most were English/Welsh/Scottish/Northern Irish/British (82%), and the remainder were from 16 other ethnic groups. All social grades were well represented (AB 24%, C1 32%, C2 16%, DE 28% [49]. Social grades are commonly defined: A, B, C1 = ‘middle class’ and C2, D, E = ‘working class’.

Participants lived across all 12 Government Office Regions, with most people in urban environments (80%), and the rest in town fringe (10%) or rural (10%) areas. People most commonly lived in semi-detached or terraced housing, or in flats or maisonettes (Supplementary Materials Figure S1), and in housing built in the 20^th^ century following the second World War (Supplementary Materials Figure S2). Most people owned or part-owned their home (Supplementary Materials Figure S3). 

Over three-quarters (77.2%) of people had a garden. Some kept cats or dogs, or they kept poultry, other birds, or pets in outdoor pens or aviaries. Some fed wildlife in their gardens, stored animal feed in an outside store, stored food waste in an outdoor bin, or made their own compost. One-fifth of people had children under 16 years of age (Supplementary Materials Figure S4).

#### 3.1.2. Attitudes towards Species

**Figure 1:**
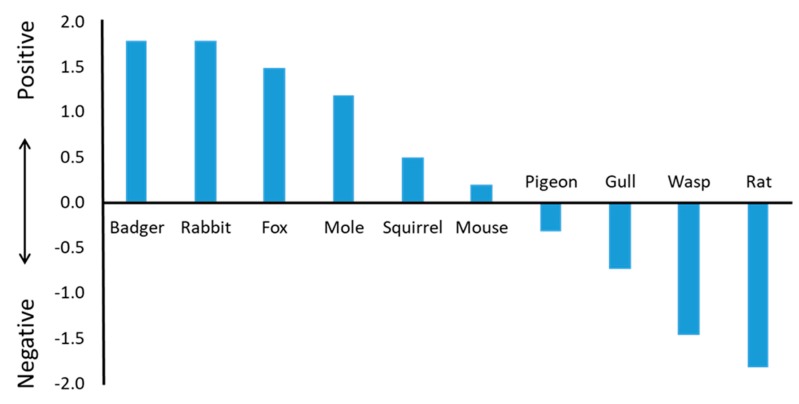
Mean attitude scores for each of the 10 species (*n* = 2000). Bars above the line represent positive attitudes and those below the line represent negative attitudes. Greater scores indicate more positive attitudes.

People were asked whether their attitude towards each species was very positive, somewhat positive, neutral, somewhat negative, or very negative (Supplementary Materials Figure S5). Responses were converted into an overall attitude score for each species. This was done by allocating the values 2, 1, 0, −1, and −2 to the scores for each of the five attitude levels ranging from very positive to very negative, and then totalling and dividing by 1000 to produce a score for each species (Figure 1). Badgers recorded the highest average and rats recorded the lowest (the only mammal with a negative average score).

#### 3.1.3. Perceived Pest Status

Most people (66–98%) said that each of the 10 species had never caused them a problem personally, while over one-third (36%) said that none of the species had done so (Table 1). Nine percent of people felt that none of the species posed problems, even generally, to people in the UK. 

While average attitude scores were related to the frequency of personal experience with a pest species (Table 1), gulls, for example, were ranked only seventh in terms of causing people problems personally but recorded the third worst attitude score, while mice recorded a positive average attitude score but were second most frequently reported to cause problems. However, mean attitude scores were more closely related to beliefs about whether species caused problems generally (Table 1).

**Figure 2:**
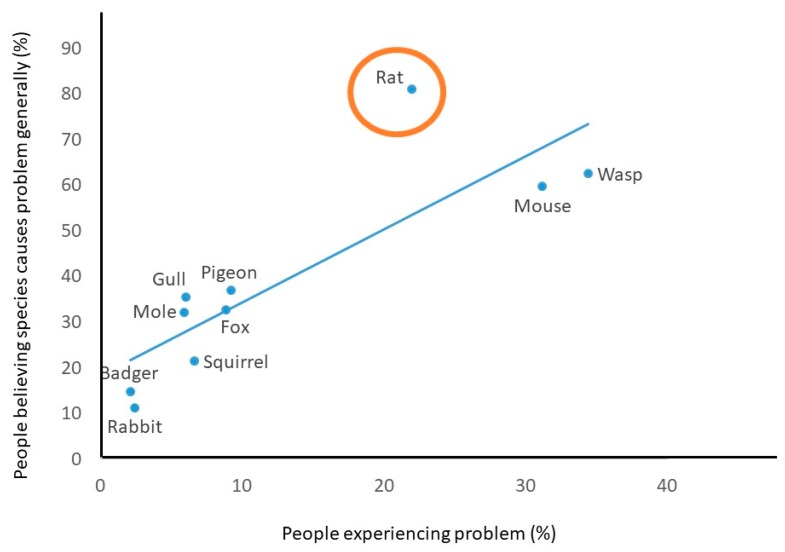
Relationship between whether people had experienced a problem with a species (percentage of people reporting) and whether they believed it to cause a problem generally in the UK (percentage of people believing) (*n* = 2000).

There was an approximately linear relationship between the frequency of personal experience of problems and believing species cause problems generally (Figure 2). As expected, more people reported perceiving a species to be a general problem than reported personal experience with that species, but this was conspicuously true of the rat (Table 1 and Figure 2).

Most people who believed that a particular species causes problems for people generally in the UK used negative language to describe the species (said they considered that species to be ‘vermin’ or a ‘pest’ or both): rats (94%), mice (91%), pigeons (90%), gulls (89%), wasps (87%), squirrels (85%), moles (73%), rabbits (71%), foxes (68%), and badgers (57%). The most popular term for rats was ‘vermin’, while mice were equally likely to be referred to as ‘pests’ or ‘vermin’, and all other species tended to be termed ‘pests’ (Supplementary Materials Figure S6).

#### 3.1.4. Perceived Species Traits

**Figure 3:**
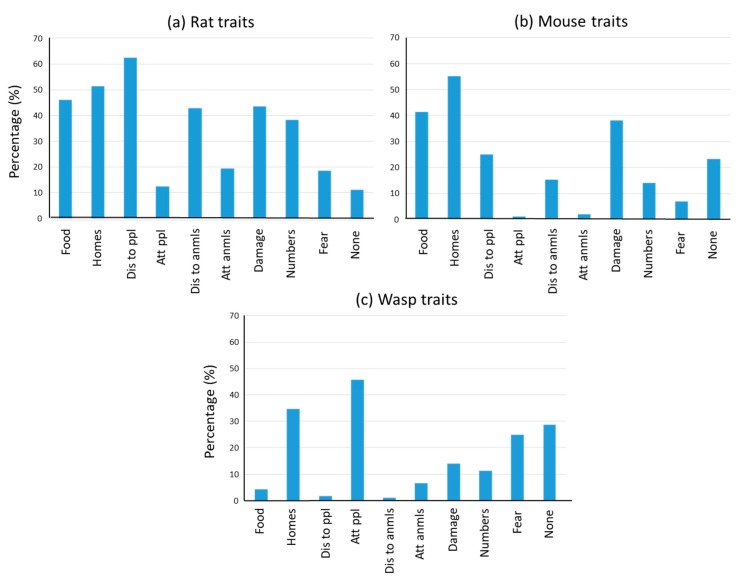
Traits attributed to: (**a**) rats; (**b**) mice; and (**c**) wasps (percentage of people attributing, *n* = 2000 per species). The traits are in order: ‘Eat our food’, ‘Enter homes’, ‘Spread disease to people’, ‘Attack people’, ‘Spread disease to pets/domestic poultry’, ‘Attack pets/domestic poultry’, ‘Damage property’, ‘Numbers are out of control’, ‘I’m afraid of them’, ‘None’.

The traits that people associated with different species varied widely (Figure 3, Figures S7 and S8). Those traits frequently attributed to the species most often reported to cause problems were ‘come into our homes’ (mice: 55%, rats: 51% and wasps: 35%) and ‘spread disease to people’ (rats: 63%), or ‘spread disease to pets/domestic poultry etc.’ (rats: 43%). Wasps were associated with the trait ‘attack people’ by nearly half of respondents (46%) (Figure 3). Many thought that rats and mice ‘eat our food’ (rats 46%, mice 41%) or ‘damage our property’ (rats 44%, mice 38%). ‘Fear’ was also conspicuous for wasps (25%), rats (19%), and mice (7%).

Almost half of participants (48%) believed that foxes ‘attack pets or domestic poultry etc.’, while around one-third felt that moles (34%) ‘damage our property’, gulls (30%) ‘attack people’, or that rat (38%), pigeon (31%) or gull (27%) ‘numbers are out of control’ (Supplementary Materials Figures S7 and S8).

#### 3.1.5. Past and Predicted Future Control

**Figure 4:**
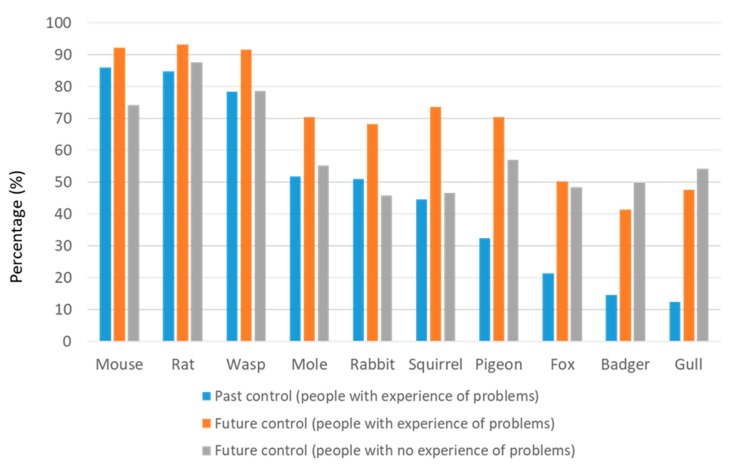
Reported past control and predicted future control rates for 10 species. Past control and predicted future control for people with experience of problems with a species (*n* values for numbers with experience of a problem: mouse = 622, rat = 438, wasp = 688, mole = 118, rabbit = 47, squirrel = 132, pigeon = 185, fox = 177, badger = 41, gull = 120) and predicted future control for people with no experience of problems (*n* values for people with no experience of a problem: mouse = 1378, rat = 1562, wasp = 1312, mole = 1882, rabbit = 1953, squirrel = 1868, pigeon = 1815, fox = 1823, badger = 1959, gull = 1880).

Most people who had experienced problems with mice, rats, and wasps said they had attempted some sort of control, while around half of people experiencing mole, rabbit, or squirrel problems had attempted to control these (Figure 4). A minority of the people reporting problems with pigeons, foxes, badgers, or gulls had used any sort of control. 

Respondents were asked whether, if they experienced a problem with each of the species in future, they would attempt some form of control. People who had experienced a pest problem were more likely to say they would opt for future control than those who had used control for a past problem, this difference being more marked where rates of past control were lowest, e.g., pigeons, foxes, badgers, and gulls (compare orange with blue bars, Figure 4).

More people with experience of a pest problem said they would opt for future control compared with those who had not experienced a problem (compare orange with grey bars, Figure 4). However, for pigeons, foxes, badgers, and gulls, predicted future control rates were roughly comparable for those with and without prior experience of problems with these species and greater than past control rates.

**Figure 5:**
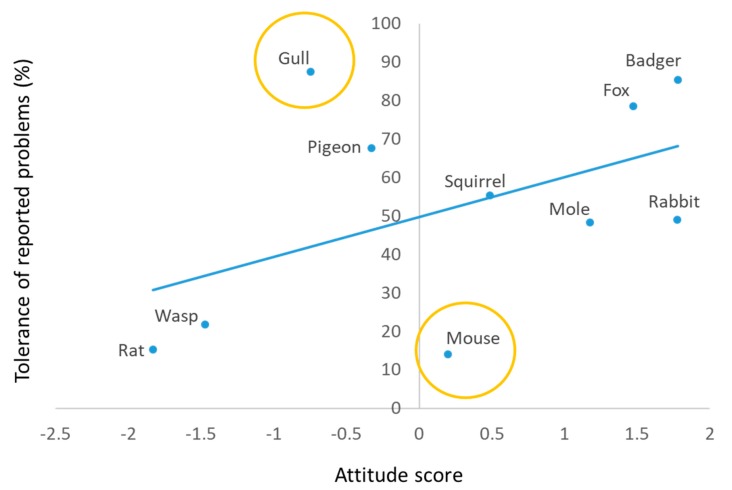
Relationship between attitude scores (*n* = 2000) and tolerance (% taking no action) of problems with species. (*n* values for respondents with experience of a problem): rat = 438, wasp = 688, gull = 120, pigeon = 185, mouse = 622, squirrel = 132, mole = 118, fox = 177, rabbit = 47, badger = 41).

Attitudes were not strongly correlated (R = 0.49, *p* = 0.154) with tolerance of problems (taking no action when a problem is experienced), but gull problems were tolerated more than might be expected, and mouse problems were tolerated less than might be expected (Figure 5).

**Figure 6:**
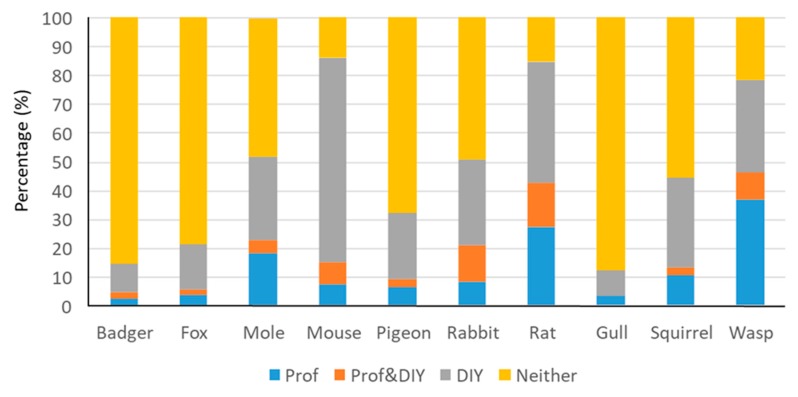
Control actions taken against 10 species where a problem was experienced in the past. Prof = Professional control service only, Prof&DIY = Professional control service and Do-It-Yourself control, DIY = Do-It-Yourself control only (percentage of people reporting, *n* values: badger = 41, fox = 177, mole = 118, mouse = 622, pigeon = 185, rabbit = 47, rat = 438, gull = 120, squirrel = 132, wasp = 688).

People who had experienced a problem with a particular species were asked whether they had used Do-It-Yourself (DIY) pest control, employed a professional pest control service, done both, or done neither. The type of control attempted in the past varied among species (Figure 6). For badgers, foxes, pigeons, gulls, and squirrels, the most common response was that no control had been attempted where a problem had been experienced, but where control had been applied, this had been done primarily using DIY pest control products. Where moles or rabbits were concerned, control was applied in around half of cases, primarily using DIY methods. Some sort of control was applied in the majority of cases where rat, mouse, or wasp problems occurred. Mouse control was largely conducted using DIY methods, while for rats, people primarily used DIY products but professional services were also important. Conversely, wasp control was most often done professionally, but DIY methods were also popular.

#### 3.1.6. Factors Influencing Choice of Control

People who had experienced a problem with species in the past were asked if they would be most likely to buy a DIY pest control product, employ a professional pest control service, do both, or do neither, if they experienced a problem with that species in future. Those saying they would use a DIY product were asked how important each of nine factors would be in choosing a DIY product for the particular species, and those saying they would employ a professional service were asked about the importance of factors in choosing a professional control service for the species. Importance ratings ranged from ‘very important’, to ‘somewhat important’, ‘neutral’, ‘not very important’, and ‘not at all important’. Then, the responses for each factor, for each species, were converted into an overall importance score, ranging from positive to negative, by allocating the values 2, 1, 0, −1, and −2 to the scores for each of the five importance levels ranging from very important to not at all important and calculating a mean for each species (Table 2).

Overall, at least seven of the nine factors were considered important (rated positively) for all species in both DIY and professional control. These seven were, in no particular order: solves problem quickly; long-lasting effect; low cost; easy to use; hygienic; safe for people and pets; and safe for non-target wild animals. In all but one case (professional control of badgers), the four factors ranked most important for both DIY and professional control of all species were: solves problem quickly; long-lasting effect; safe for people and pets; and safe for non-target wild animals (marked in bold in Table 2). While the factors ranked highest varied among species and control type (DIY/Professional), people were primarily concerned that DIY control for rats, mice, and wasps should solve problems quickly, while professional control should be quick for rats and wasps and lasting for mice.

**Figure 7:**
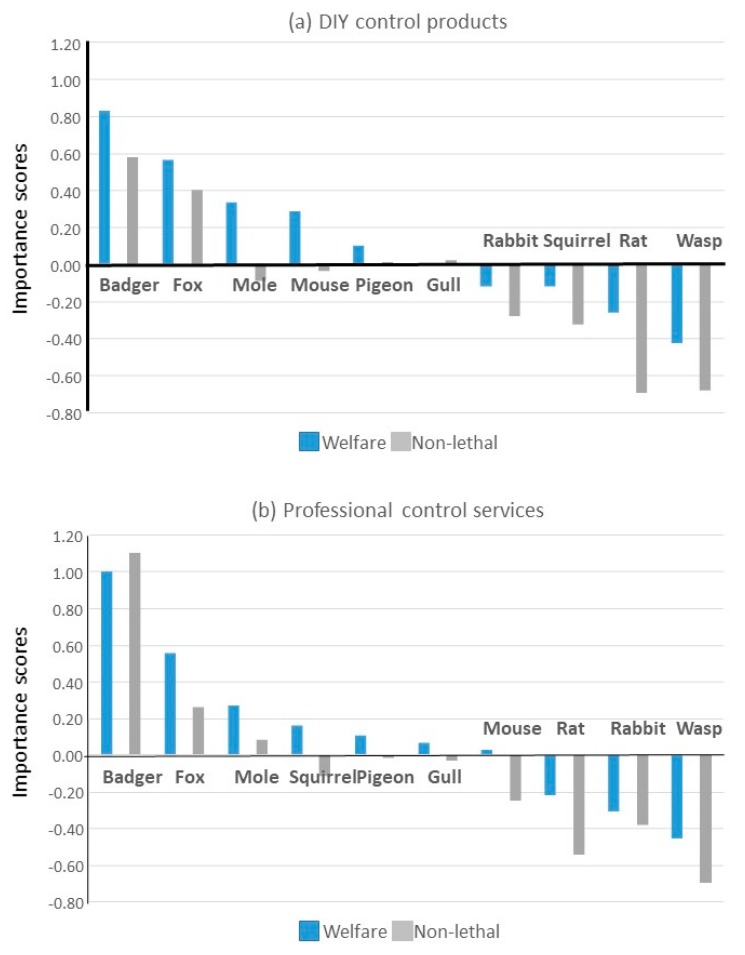
The importance of welfare-friendliness and non-lethality in choosing: (**a**) DIY pest control products; or (**b**) professional pest control services (*n* values as per Table 3). Positive importance scores (bars above horizontal axis) indicate that a factor is considered important in choosing control, and greater scores indicate greater importance.

Despite generally being ranked below the other factors in terms of importance for choosing pest control products and services, non-lethal and welfare-friendly approaches were nevertheless often considered somewhat important (rated positively) (Table 2 and Table 3, Figure 7). Welfare-friendliness was considered important in choosing both DIY products and professional services for controlling most species, including badgers, foxes, moles, mice, pigeons, gulls, as well as professional squirrel control services. Control being non-lethal was thought to be important in choosing DIY products and professional services for controlling badgers and foxes, as well as DIY products for both bird species and professional mole control services. In general, people felt that it was more important that control was welfare-friendly than non-lethal (Figure 7).

#### 3.1.7. Drivers of Public Attitudes towards Species

People’s attitudes towards species were successfully modelled for all species except squirrels and foxes (for which models failed to converge). Attitudes towards each species were driven by multiple factors, with evidence for effects of between 10 and 15 wide-ranging factors depending on species; these included factors related to human demography, homes, behaviours, and perceptions about species and species traits (Table 4). The large number of drivers involved indicate the complexity of attitudes towards wildlife. Summary tables associated with attitudes models are available in Supplementary Materials (Document S2).

Several factors were universally associated with negative attitudes (Table 4, Supplementary Materials Figure S9). People believing that a species causes problems generally in the UK, that the species spreads disease to people or that their numbers are out of control, or people who were afraid of the species had more negative attitudes towards them. Likewise, people who had experienced problems personally with any species (except badgers) also had more negative attitudes towards the species. Other factors associated with people having negative attitudes towards some species were beliefs that the species attacks people (rabbits, rats, gulls, and wasps) pets, or poultry (mice, gulls, and wasps), that it damages property (badgers, moles, rabbits and wasps), people being older (rats, rabbits, mice, and moles) and people having children (badgers and mice) or using an outdoor feed store (moles and mice). Men tended to have a more negative attitude to rabbits. 

People in older homes tended to have more positive attitudes. People with cats or dogs had more positive attitudes towards all of the mammal and bird species. People who fed wild birds or animals in their garden had more positive attitudes towards all species except rats.

#### 3.1.8. Drivers of Perceived Pest Status: Personal Experience of Problems with Species

People’s own experiences of problems with species were successfully modelled for all species except squirrels and wasps (for which models failed to converge). Experience of problems with each species was driven by 6–10 wide-ranging factors, including some related to human demography, homes, behaviours, and perceptions about species and species traits (Table 5). Drivers of experience of rat and mouse problems were particularly numerous, indicating the complexity of their pest status. Summary tables associated with models are available in the Supplementary Materials (Document S3).

**Figure 8:**
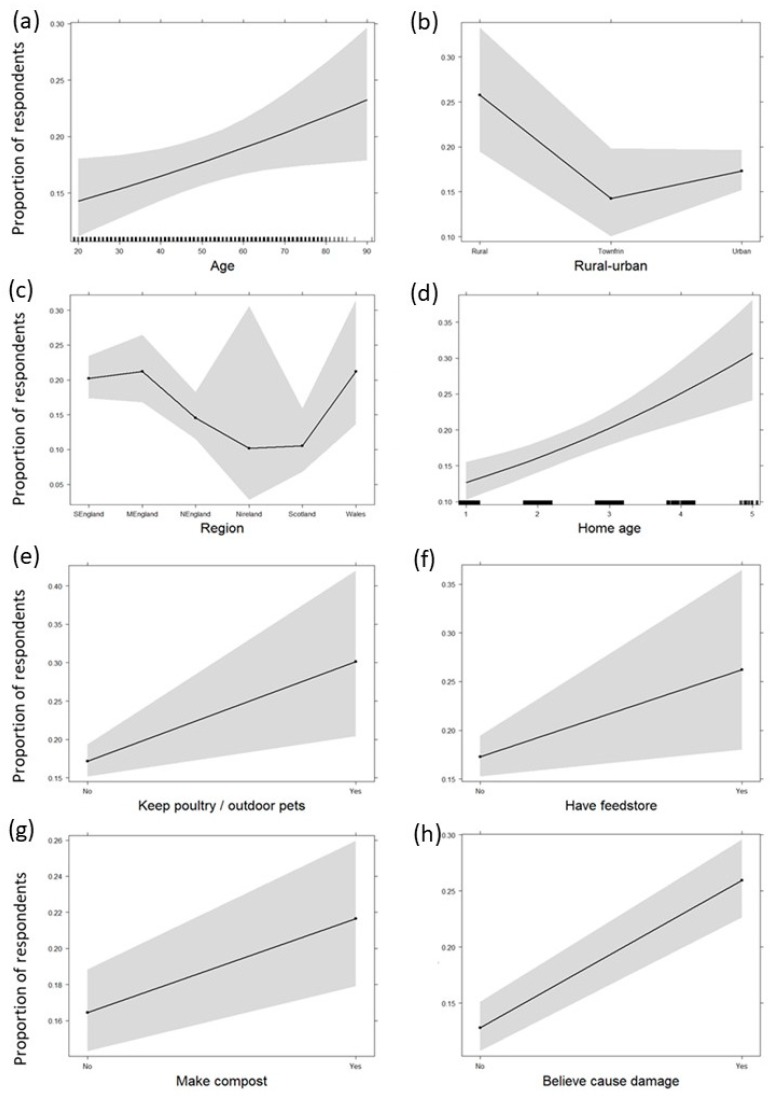
Drivers of respondents’ experience of problems with rats (marginal effects in logistic regression models), including: (**a**) respondent age; (**b**) whether respondent lives in an urban or rural area; (**c**) region; (**d**) home age; and whether respondent (**e**) keeps poultry or outdoor pets; (**f**) has an outdoor feedstore; (**g**) makes compost; or (**h**) believes that rats damage property. Confidence Intervals are shown in grey. Geographical areas are: Southern England, Middle England, Northern England, Northern Ireland, Scotland, and Wales. Home age categories are: 1 = 21^st^ Century (≥2001), 2 = Post-World War II (1946–2000), 3 = Pre-World War II (1902–1945), 4 = Victorian (1837–1901), and 5 = Pre-Victorian (≤1836).

There were noticeable similarities among several species in the factors associated with experience of pest problems (Table 5). People with a more negative attitude towards a species or who believed that a species causes problems for people generally in the UK were also more likely to report experiencing problems personally with that species. Older people and people living in older homes were more likely to report having problems with several of the species, including rats and mice. Experience of problems varied with geographic area for some species, mouse problems being more prominent in Northern Ireland, Scotland, and Southern England and rat problems more common in Middle England, Wales, and Southern England. People living in rural areas were more likely to report problems with mice, rats, rabbits, and moles, while men were more inclined to report problems with pigeons, gulls, and rabbits. People making their own compost or storing animal feed in an outdoor feed store more often reported problems with some species, and those keeping pets or domestic poultry in outdoor pens more often reported having problems with rats. People believing that a species causes damage to property tended to report problems with that species, while those believing that mice enter homes or spread disease to people more often reported having mouse problems. See Figure 8 for some example plots showing effect sizes for rats and Supplementary Materials Figure S10 for plots for all species.

#### 3.1.9. Drivers of Perceived Pest Status: Beliefs about whether Species Cause Problems for People Generally in the UK

Beliefs about whether species cause problems for people generally in the UK were successfully modelled for all 10 species. In contrast to factors driving the personal experience of problems, many factors driving general beliefs were related to human perceptions about species and species traits, and fewer were related to human demography, homes, and behaviours (Table 6). Summary tables associated with models are available in the Supplementary Materials (Document S4).

Older people were more inclined to say that pigeons, gulls, squirrels, and moles cause problems, while people in older homes more often felt that rabbits and pigeons cause problems. Urban dwellers tended more often to believe that foxes cause problems generally. Beliefs varied with geographic area for badgers and gulls, with badgers more often thought to cause problems generally by people living in Northern Ireland and Southern England, and gulls more often thought to cause problems by people in Scotland and Northern England. People with a garden were more likely to believe that squirrels cause problems generally, while those storing food waste in an outdoor bin for collection by the council were more inclined to believe the same of mice, foxes, and rabbits.

A wide range of species traits were identified as factors in public beliefs about species causing problems generally. For most species, beliefs that they damage property or that their numbers are out of control were (unsurprisingly) associated with beliefs that a species presented a problem to people generally. In addition, for example, other factors for rats included the perceptions that they enter homes and spread disease to people, while other factors for mice were the beliefs that mice enter people’s homes and eat their food, and those for wasps included the perceptions that wasps enter homes, attack people, pets, or poultry, and that people are afraid of them. Many of these perceived traits reflect traditionally held views about species, which will be the main source of information for most people, who have not experienced the species first-hand (see Perceived Pest Status, above). See Supplementary Materials Figure S11 for plots showing effect sizes.

#### 3.1.10. Drivers of Perceived Pest Status: Use of Negative Language: ‘Pest’ and ‘Vermin’

People who believed that a particular species causes problems for people generally in the UK were asked if they would refer to the species as a ‘pest’, ‘vermin’, both, or neither. The use of such negative language was modelled for all species using just perceived species traits as potential drivers. Disease, damage to property, fear, and a perception that numbers were too high tended to predict approval of negative terms (Table 7). Summary tables associated with models for individual species are available in the Supplementary Materials (Document S5). See Supplementary Materials Figure S12 for plots showing effect sizes.

#### 3.1.11. Drivers of Public Demand for Pest Control: Past Control of Species

People who had previously experienced problems with a species were asked if they had conducted any sort of control (bought DIY products or used professional control services). Whether or not they had conducted control was successfully modelled for rats, mice, wasps, and moles (models for other species failed to converge). Past control tended to be predicted by far fewer and more diverse factors compared with ‘pest status’ (Table 8). Summary tables associated with models are available in the Supplementary Materials (Document S6). Drivers varied widely among the species, indicating that when a problem is experienced, deciding whether to conduct control is species-specific (Supplementary Materials Figure S13). 

Among people previously experiencing rat problems, rat control was more often attempted by older people and by those thinking that rats spread disease to pets and domestic poultry, and less likely to be attempted by people that owned a cat or dog or who were afraid of rats. Mouse problem control was more often attempted by people that owned their own homes or lived in privately rented or social housing, and by those with a negative attitude towards mice or who think that mice enter people’s homes.

#### 3.1.12. Finding Help with Wild Animal Problems

Respondents were asked where they would look for information on dealing with a wild animal problem on their property. Responses were internet search (73%), local council (56%), pest control company (36%), wildlife protection group (29%), government website (25%), hardware store (17%), garden centre (16%), do nothing (4%), and other (3%).

#### 3.1.13. ‘Controlling’ Infestations

People were asked who they thought should control a wild animal infestation on a resident’s property and who should pay for this control. They were allowed to select multiple responses. Responses regarding who should control the infestation were the resident (44%), the owner (37%), the local council (33%), whoever is at fault (25%), don’t know (22%), and that the animals should not be disturbed (11%). Responses regarding who should pay were the owner (40%), followed by the resident (34%), whoever is at fault (27%), the local council (27%), don’t know (23%), and that the animals should not be disturbed (9%).

### 3.2. Council Freedom of Information (FOI) Requests

The survey achieved a response rate of 100% of those councils that provided some sort of pest control service in 2017/18 (*n* = 309), although 15 councils withheld some data, citing exemption 43 under the FOI Act 2000, to protect the councils’ commercial interests.

#### 3.2.1. Availability and Delivery of Local Authority Public Pest Control Services

**Figure 9:**
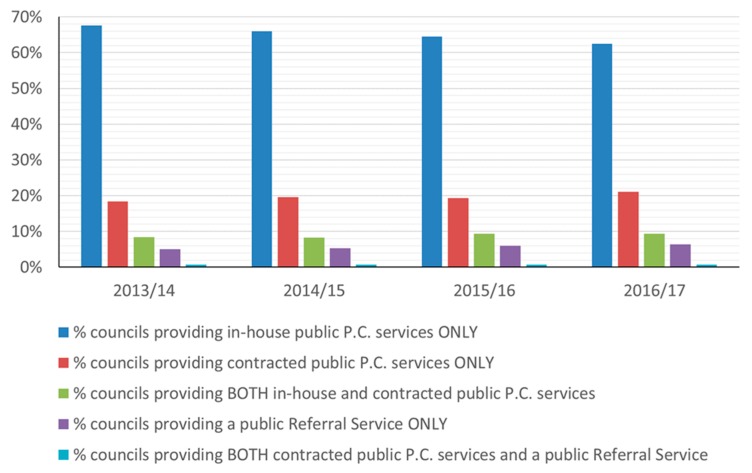
The proportion of UK councils providing each type of public pest control service out of all the councils providing a service in 2013/14 (*n* = 299), 2014/15 (*n* = 300), 2015/16 (*n* = 299), and 2016/17 (*n* = 299).

The majority of councils provided an in-house only pest control service throughout the study period. The proportion of councils offering in-house services decreased each year between 2013/14 and 2016/17, while the proportion using a contractor, operating a combined in-house and contracted service, or providing a referral service increased (Figure 9).

The in-house only service type was also the service most frequently offered in each of the 12 UK Government Office Regions (GORs) during the four-year period. The service types offered differed significantly between geographical areas (excluding Northern Ireland for which data were too few) (LR χ^2^ = 221.5, 16 dof, *p* ≤ 0.001, Supplementary Materials Figure S14), with in-house services being least available in Scotland and Southern and Middle England, but there was no evidence of a temporal trend (LR χ^2^ = 2.4, 4 dof, *p* = 0.663).

#### 3.2.2. Annual Expenditure, Revenue, and Losses Related to Public Pest Control

The mean gross annual expenditure and gross annual revenue related to control were calculated per council (for those councils providing data for each year). Mean gross annual expenditure decreased between 2013/14 and 2016/17, from £161k (*n* = 261) to £146k (*n* = 264), while, overall, the mean gross annual revenue increased from £87.0k (*n* = 270) to £95.5k (*n* = 277). 

We converted the gross annual expenditure and gross annual revenue data into figures per 1000 households (using data from the 2011 Census [50]). Between 2013/14 and 2016/17, the mean gross annual expenditure per 1000 households per council decreased significantly from £2.39k to £1.96k (F_1,781_ = 48.389, *p* < 0.001, Supplementary Materials Figure S15), but there was no statistically significant difference in expenditure among the geographical areas (excluding Northern Ireland) during this time (F_4,264_ = 1.318, *p* = 0.264). Meanwhile, the mean gross annual revenue per 1000 households, per council, varied significantly among years (£1.12k, £1.19k, £1.14k, £1.14k, for the four years respectively, F_1,814_ = 12.368, *p* < 0.001) and among geographical areas (£0.73k, £1.25k, £1.3k, £1.2k, and £1.01k, for Middle England, Southern England, Northern England, Scotland, and Wales respectively, F_4,276_ = 2.524, *p* = 0.041). 

Financial losses and gains data were generated for councils providing both expenditure and revenue figures for each year. The proportion of councils incurring a net annual loss decreased from 91.1% (*n* = 258) to 85.2% (*n* = 263) between 2013/14 and 2016/17, and the proportion generating a net annual gain increased accordingly (Supplementary Materials Figure S16). The mean net annual loss per council decreased from £73.7k (*n* = 258) to £48.3k (*n* = 263) over the four years, while the mean net annual loss per 1000 households, per council, decreased from £1.28k to £817 over the four years (Table S3). It was not possible to examine any possible trend in losses over time, because the pattern of losses over time differed among the five geographical areas (excluding Northern Ireland) (LR χ^2^ = 10.842, 4 dof, *p* = 0.028).

#### 3.2.3. Species Treatments Offered and Requested

Our preliminary council website search identified rats, mice, wasps, foxes, pigeons, grey squirrels, moles, and gulls as the only species/animal groups treated by councils (alongside cockroaches, bedbugs or ants). Subsequently, in the FOI survey, we asked councils to report the annual number of requests (including requests for call-out visits, on-site treatment visits, and repeat treatment visits, but not requests for advice provided over the phone or in writing only) that they responded to each year for each of these species. Many did not keep separate records of treatment requests for rats and mice and therefore provided only combined data for these species. The combined data generally followed similar patterns to those for the separate rat and mouse data, so the combined data were excluded from these analyses unless specified.

**Figure 10:**
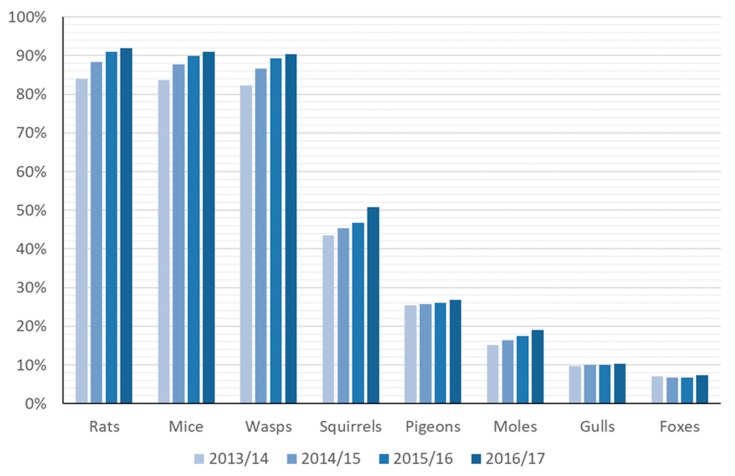
Annual proportion of councils offering public pest control per species out of all UK councils providing public pest control for one or more of the species selected for the study for 2013/14 (*n* = 299), 2014/15 (*n* = 300), 2015/16 (*n* = 299), and 2016/17 (*n* = 299).

The species for which councils most often provided public pest control services were rats, mice, and wasps (Figure 10). Over the four years, the proportion of councils providing treatments increased each year for rats, mice, wasps, squirrels, pigeons, and moles, and there was an overall increase for gulls and foxes. The overall ranking of the species remained the same throughout.

Councils who offered control services for one or more of the eight species received a total of half a million treatment requests per year (414k, 497k, 443k, and 465k for 2013/14, 2014/15, 2015/16, and 2016/17, respectively). By far, the largest number of requests received each year concerned rats (197k–249k p.a.), followed by mice (110k–126k p.a.) and wasps (80k–114k p.a.) (Table S4). In 2016/17, the combined number of requests for these three species made up 98.3% of the total number of requests for the selected species, around half of that figure being for rats. The mean annual number of treatment requests received by a council offering public pest control for any of the eight species (including combined data for rats and mice) was 17.9, 21.6, 19.5, and 20.5 requests per 1000 households over the four years, respectively.

The mean annual numbers of treatment requests received per species per council varied among 10.1–12.3, 4.6–6.1, and 5–5.6 for rats, wasps, and mice, respectively per 1000 households (Table S5). The pattern of treatment requests between geographical areas (except Northern Ireland) varied over time (Rats: F_4,775_ = 5.12, *p* < 0.001; Mice: F_4,767_ = 5.30, *p* < 0.001; Wasps: F_4,769_ = 13.07, *p* < 0.001), so it was not possible to look separately at trends in treatment requests over time or differences in treatment requests among areas.

#### 3.2.4. Provision of Free Pest Control Treatments

In our preliminary 2017/18 council website survey, we found that while some councils offered free treatments for certain species to all domestic properties, others offered free services only to certain individuals, including benefit recipients, older people, pensioners, and council housing tenants. Of the 309 councils providing some form of public pest control, in 2017/18, 18.1% of councils offered free treatments for rats, and 10.7% of councils offered free treatments for mice (Supplementary Materials Figure S17). Only one council provided free treatment of domestic properties for wasps or squirrels, while no councils offered free treatments for foxes, pigeons, or moles.

#### 3.2.5. Provision of Prevention and Deterrence Advice

In 2017/18, only 50.3% (*n* = 390) of councils provided their own prevention and deterrence advice to the public through their website. The remaining half of local authorities either supplied no information for any species, or simply referred people to services or websites run by external third parties (often private pest control companies). The availability of advice varied among GORs (Supplementary Materials Figure S18), with the largest and smallest proportions of councils offering advice in the East Midlands (77.5%, *n* = 40) and Wales (18.2%, *n* = 22), respectively. In 2017/18, the councils most likely to offer their own prevention and deterrence advice were those operating an in-house service (54.1%, *n* = 242), followed by councils offering no public pest control service (46.9%, *n* = 81) and then those using a contractor (40.3%, *n* = 67).

There was considerable variation in the proportion of all councils (*n* = 390) offering prevention and deterrence advice for each species: rats (40.5%), mice (34.1%), pigeons (20.8%), foxes (19.7%), squirrels (17.9%), wasps (6.2%), and moles (2.3%). Among councils that did supply advice, there were notable differences in the quality, coverage, level of detail, and format, and information was not standardised across councils. Some supplied their own fact sheets for species, with guidance on prevention and deterrence measures, while others offered very limited or generic information.

## 4. Discussion

Public attitudes to 10 wildlife species commonly regarded as problematic in the UK varied widely between the most and least popular (badgers and rats, respectively). Attitudes were not strongly associated with people’s experience of problems. For example, mice were on average viewed positively despite causing problems for almost one-third (31.1%) of respondents. Gulls were generally viewed negatively, despite rarely (6.0%) causing problems. Two-thirds or more (≥65.6%) of people reported never having problems with each of the 10 species, and 35.6% had never had a problem with any of them. Where problems were reported, people often reported taking no action to mitigate the problem, except where mice, rats, or wasps were involved. Levels of tolerance, taking no action when presented with a problem, were not well predicted by people’s attitudes, with mice being less tolerated than expected, on average, and gulls being more tolerated than expected. Rats had a conspicuously worse reputation for causing problems than predicted by people’s personal experiences. 

DIY control methods were preferred over professional services for all species except wasps. The safety of non-target wildlife was frequently prioritised in choosing control methods, while concerns for target species were not. While not a priority, the welfare-friendliness of pest control and non-lethal control were nevertheless considered ‘important’ (received a positive mean importance score) for some species, and there was evidence that even where people accepted lethal control, they were concerned that it should be welfare-friendly. While attitudes and measures of pest status were each driven by multiple and varied factors, whether people reported past efforts to control rat, mouse, wasp, and mole problems was driven by very few, species-specific variables. For example, control efforts tended to be linked to concerns about mice and wasps entering homes and rats spreading disease to pets or domestic poultry. Associating negative language with a species was largely driven by beliefs that species’ numbers were out of control and that species spread disease or cause damage.

Most councils surveyed offered pest control services for rats, mice, and wasps, probably reflecting public demand for help with these pests in the face of clear health risks and, in the case of rats and mice, councils’ duties under the Prevention of Damage by Pests Act (1949) to “take such steps as may be necessary to secure so far as practicable that their district is kept free from rats and mice” (see Part I, Section 2(1), http://www.legislation.gov.uk/ukpga/Geo6/12-13-14/55/section/2). The proportion of councils doing so increased between 2013/14 and 2016/17. These species also generated most of the treatment requests from the public, almost half being for rats. A clear deficiency from the perspective of cost-effectiveness is that only half of councils provided advice on preventing or deterring wildlife, this being more likely where councils offered in-house control. Advice on preventing or deterring wasps was particularly seldom available.

### 4.1. YouGov^®^ Survey

#### 4.1.1. Attitudes

Attitudes were often linked to completely rational reactions to the activity of different species, but this was not always the case; indeed, it has been observed that attitudes towards non-human animals are “mired in an uncomfortable mix of logic and emotion” [39]. Rats and wasps were the species viewed most negatively by the UK public, this chiming with Kellert’s [51] finding that rats and wasps were the least liked of 33 animals by the U.S. and Alaskan public, after cockroaches and mosquitoes. The other two negatively viewed species in our study were the birds, pigeons, and gulls. All of the positively viewed species were mammals, the best-liked being badgers, rabbits, and foxes, which supports Kellert’s finding that people prefer larger animals [51]. As here, Dunn et al. [52] found that the UK public viewed squirrels positively. Most respondents lived in urban environments and may be less likely to encounter some of the more positively viewed species. However, an increasingly urbanised population may develop more negative attitudes towards wildlife over time if people grow up without experience of nature [53].

People tend to relate more easily to mammals [54,55] and may attribute them with greater sentience than other taxa [56], while there is no reliable evidence that most invertebrates, including wasps, are sentient [57]. If people do feel a greater empathy with mammals, this may at least partly explain the attitudes observed here. For example, while house mice are widely considered pests in the UK and elsewhere, respondents viewed mice positively, along with other mammalian species that are sometimes considered problematic. Rats were the exception to this rule, being the most negatively viewed species, despite being a mammal. This apparent anomaly might be explained in part by the fact that people still associate rats with spreading the bubonic plague (or ‘Black Death’), which killed 60% of Europe’s human population in the 14^th^ century, although the rat’s role in this remains a matter of debate [58]. According to Lambert et al. [4], rats evoke fear, repulsion, and fascination among the public. They are probably regarded as the world’s greatest mammalian household pest. 

Pigeons and gulls have increasingly been portrayed as pests by the media [59]. In the past 50 years, gulls from coastal regions have been attracted to inland urban areas, where they take advantage of food waste and warmer and safer nesting opportunities [7]. Gulls have recently received a significant amount of negative media attention over ‘anti-social behaviour’ [60], and pigeons are frequently referred to as “rats with wings”, since the term was coined in 1966 [61]. In general, people have negative attitudes towards wasps [62]; they are frightened of them [63] and wasps are commonly regarded as anything from a minor nuisance, around food and sugary drinks, right through to a dangerous stinging insect sometimes causing fatal anaphylaxis [8,64]. According to Sumner et al. [9], wasps are universally disliked by the public because people strongly associate them with their ability to sting and fears regarding wasps may have been exacerbated by recent media reports of the arrival in the UK of larger, more aggressive Hymenopteran species such as Asian hornets [65].

#### 4.1.2. Perceived Pest Status

Overall, reported personal experiences of problems were strongly related to beliefs that species cause problems for people generally, and these beliefs were strongly related to attitudes. However, attitudes were less strongly related to experiences. Attitudes arise from an inner framework of beliefs, values, and emotional components [66]. Some of these, such as people relating more easily to mammals, have been mentioned above, perhaps explaining why attitudes towards mice are positive overall despite one-third of people reporting mouse problems, and why attitudes towards gulls are negative when they rarely cause problems. The lack of a strong relationship between attitudes and experience of problems may seem unexpected. However, while attitudes towards wildlife are thought to be more strongly influenced by costs than benefits [67], studies have shown that negative attitudes towards wildlife are often not related to experience of significant damage [68,69]. In fact, attitudes may be more strongly related to intangible costs such as perceived threats (e.g., fear of attack [70], psychological costs of danger, risk, or fear [67], cultural and social perceptions of potential threat [71]). 

Beliefs concerning different species could arise through personal contacts such as friends and relatives who have experienced problems, or from persistent beliefs in the population, myths, or alarmist media reporting (e.g., [72,73]). Beliefs that rats cause problems were conspicuously greater than might be expected based on people’s experience. This could be related to the wide range of negative traits associated with rats and the high rates of belief in these, compared to other species (Figure S7), or to long-standing public mistrust relating to disease transmission. 

Indeed, looking at the traits attributed to rats, mice, and wasps, it is no surprise that these were the most frequently cited problem species in this study. All three species were perceived as being far more likely to enter homes, while rats were considered much more likely to spread disease to people and wasps to attack them. Given the personally intrusive and threatening nature of these perceived traits, it is logical that these were also the most feared species (wasps 25%, rats 19%, mice 7%).

The four species most frequently thought to possess none of the nine species traits (rabbits, squirrels, moles, and badgers) were those thought least likely to cause general problems and four of the five least often reported to have caused respondents personal problems. Given that gulls were seldom reported as causing personal problems and the relatively low rates with which traits were attributed to them, their general unpopularity seems counter-intuitive until you consider their portrayal in the media [60] and even in parliament [74]. The species associated with the worst attitudes (rats, wasps, gulls, pigeons, mice, and squirrels) were also those most often described using negative language. Both bird species are among these, and given that one current definition of vermin is “animals that are harmful, annoying and often difficult to control” [75], this could be related to the fact that pigeons, gulls, and rats were the species whose numbers were most often thought to be ‘out of control’. Using words such as ‘vermin’ or ‘pest’ to describe wildlife species may create the impression that it is acceptable to treat them with less ethical or welfare consideration (see [38]).

#### 4.1.3. Past and Predicted Future Control

Pest control rates might be expected to reflect the actual level of nuisance that a species causes and the control options available. We defined ‘tolerance’ as a person not using control despite reporting problems with a species. The majority of people with rat, mouse, or wasp problems attempted to control them, and it is likely these were the least tolerable species because of the intrusive and personally harmful nature of the problems they are perceived to cause. In addition, in theory, under the Prevention of Damage by Pests Act (1949) (https://www.legislation.gov.uk/ukpga/Geo6/12-13-14/55/contents), it is the duty of local authorities to keep their district free from rats and mice, and of the occupier to inform the local authority “if rats or mice are living on or resorting to the land in substantial numbers”. However, it is unlikely that this was a factor in most domestic circumstances. For moles, rabbits, and squirrels, approximately half of people reporting problems had attempted some sort of control. At the other end of the spectrum, people rarely attempted to control problems they reported with pigeons, foxes, badgers, and gulls (note that both bird species are included here). 

Ngo et al. [53] found that attitude towards wildlife was the strongest predictor of tolerance to ‘nuisance animals’ in Singapore, but this was not always the case in our UK study, given that mice were viewed positively and gulls were the most tolerated species. Perhaps problems with birds, badgers, and foxes were more tolerable because they occurred largely outdoors and were associated with more temporary or fleeting incidents. Furthermore, because badgers and birds are protected in the UK, the available control options are limited and even if problems with these animals were equally intolerable as those with other species, they may be harder to do anything about. For example, although 44% of the UK population supports the idea of a gull cull [76], birds are protected, and so options for domestic gull control are limited, e.g., to physical deterrents (such as bird spikes or netting), chemical deterrents, or changing the behaviour of many people (such as not feeding gulls) [74]. In addition, problems with gulls may be ephemeral, e.g., defecating, dive-bombing people, and thus annoying but tolerable. On the other hand, mice are not protected and despite having the allure of mammals, they are most commonly associated with entering people’s homes and eating their food, which are both intrusive and likely intolerable problems.

Members of the public clearly consider DIY methods central in managing most species, but both professional services and DIY methods are deemed important for managing rats and wasps. People might choose DIY over professional control in many cases because they wish to retain control over how and when it is done and the cost. They may wish to eliminate the problem as soon as possible, or they might want control to be welfare-friendly for the target animal, but fear that a professional service will be expensive, slow, protracted, or will not offer welfare-friendly options. Since people tended to opt for DIY measures for managing all species except wasps, they might also opt to use DIY pest prevention measures, such as proofing products, if these were more readily available. Preventative methods are an essential and under-used component of effective rodent control [18]. However, proofing homes and other property is not necessarily straightforward, so there would likely also be public demand for professional proofing and other pest prevention services, such as those offered by Superproof^TM^, Total Pest Control, and Pestology, if a preventative approach was better promoted. Manufacturers of pest control products and providers of pest control services may be missing an important opportunity by not meeting the need for prevention and proofing materials and services.

#### 4.1.4. Important Factors in ‘Pest Control’

In general, people wanted control to be quick, long-lasting, and safe for people, pets, and non-target wild animals, with the primary concern for DIY rat, mouse, and wasp control, and professional rat and wasp control being that it is quick and that for professional mouse control being that it is lasting. It is noteworthy to see public concern for non-target wildlife, this being routinely ranked above control being easy, cheap, or hygienic. However, it was also noteworthy that for each potential target species, except for the professional control of badgers, welfare-friendly and non-lethal control were ranked last among public concerns. This may be no surprise given that the welfare of pest animals has generally been undervalued [18,30]. It might seem inconsistent to value the life and welfare of one wild vertebrate so much less than that of another, but less humane treatment may be tolerated because of a perceived ‘need’ to control certain species [18,77]. Indeed, less humane control methods are allowed in the UK for certain species. For example, unregulated spring traps may be used only with rats, mice, and moles [32], glue traps are allowed for rats and mice (although banned in Northern Ireland, New Zealand, and parts of Australia), strychnine poison could be used exclusively with moles until it was withdrawn in 2006 [6], while moles, rats, and rabbits can be gassed using phosphine, and rabbits and foxes may be caught in snares. 

Although welfare-friendliness and non-lethality were generally ranked least important, welfare-friendliness was considered somewhat important (scored positively on average) for most species (all except wasps, rats, and rabbits (and squirrels subjected to DIY control)), and non-lethality was considered important for some (badgers and foxes [and gulls and pigeons in DIY control and moles in professional]). The fact that people considered welfare-friendliness more important than whether control involved killing suggests that even where people accepted lethal control, they felt that this should involve minimal suffering. This is a very important point because it is often assumed that non-lethal equates to ‘humane’, and lethal equates to ‘inhumane’, but this is not necessarily the case [33,34,35]. The observed public concern for welfare in pest control is supported by reports that public concern for wild animal welfare is increasing [30,78] and by negative customer reviews left on the Amazon website (although this product is no longer available today) for a sealed mouse-killing trap that did not routinely kill trapped mice quickly and could not be opened to humanely kill the injured mouse [79].

#### 4.1.5. Modelling—Attitudes

Factors influencing attitudes towards species were numerous and varied, suggesting that drivers of attitudes towards the species are complex. Many of these factors related to people’s beliefs, either about species causing problems for people generally or about species traits, such as spreading disease to or attacking people, pets, or poultry, creating fear or their numbers being out of control. This is supported by previous work that suggested attitudes may be strongly related to intangible costs such as perceived threats [67,70,71]. People’s prior experience of problems was also a factor (see other examples regarding golden langurs (*Trachypithecus geei*) in Bhutan [80] and wildlife in Tanzania [81]). Older people had more negative attitudes towards some species; this may be a generational response and/or attitudes may tend to become more negative as people age, and older people will have had longer to accumulate negative associations with species. People with children were more negative about mice, perhaps because of fears regarding hygiene and the spread of disease [82] particularly to young children, who play on the floor and put their fingers in their mouths. People storing animal feed in an outdoor feed store were more negative towards moles and mice. They may have concerns about mice accessing, eating, and contaminating stored feed [82]; however, moles are unlikely to cause this sort of damage because they are omnivores preferring earthworms and other soil invertebrates [83].

People living in older homes, keeping cats or dogs, or feeding wild birds were generally more positive towards wild species and may be more in touch with and sympathetic towards wildlife. People making compost were more positive to badgers and mice. One study found that pet owners preferred closer links with wildlife and were more negative about hunting [84]. People feeding wildlife in our study were not more positive towards rats (which may eat food left out for other species); however, Bjerke et al. [85] found that an association between keeping pets and positive attitudes towards wildlife may not extend to less popular wild species. People living in older houses tend to have larger gardens, while people who make compost are likely to be interested in gardening, and dog owners are likely to enjoy being outdoors; each will have greater exposure to wildlife. Greater exposure to nature [53] or keeping pets [86], during childhood, are both linked with more positive attitudes towards wildlife, and since interests are often shared within families, then attitudes may also be shared.

#### 4.1.6. Modelling—Experience of Problems

Factors affecting the experience of wildlife problems were generally rational and less complex than factors affecting attitudes. Our finding that problems with rats and mice were associated with older homes and the rural, rather than the urban environment, were supported by data from the English House Condition Survey (EHCS) and the English Housing Survey (EHS) collected between 1996 and 2010 [4,5]. Urban rodent populations may be easier to manage, as there should be fewer opportunities for re-invasion following management in urban areas [4], while older properties may be less rodent-proof and have more mature gardens, providing more suitable rodent habitat [5]. A link with keeping poultry or outdoor pets is also supported by the EHCS/EHS data, which showed that rodent prevalence was greater where pets or other animals were kept outdoors [4,5]. The presence of an outdoor feed store was associated with both rat and mouse problems. Keeping animals outdoors or having an outdoor feed store may provide rodents with access to both food and stored bedding, which can provide harbourage [87]. Making and storing compost can attract rats, and our data showed an association between people making compost and reporting rat problems; however, measures can be taken to protect compost from rats [88]. People believing that species cause damage or cause problems for people generally and negative attitudes were also widespread predictors of people experiencing problems with those species. Beliefs about mice entering homes and spreading disease to people, and about fox numbers being out of control were also factors predicting mouse and fox problems, respectively, while people thinking that badgers spread disease to people (which is unlikely) were less likely to have reported experiencing badger problems themselves, indicating that they may not be well informed about this. Our finding that rat problems were most common in Middle England tallies with previous studies that found the West Midlands to have the greatest prevalence in England of rats living in the vicinity of people’s homes [4,5].

#### 4.1.7. Modelling—Beliefs about Species Causing General Problems

The factors predicting people’s beliefs about species causing problems for people generally were quite different to those predicting personal experience of problems. Whereas experience tended to be driven by a wide variety of factors (including many demographic, home, and human behaviour variables, as well as beliefs and attitudes), the main drivers of beliefs about species causing problems generally were beliefs about specific animal traits, together with attitudes and experience of problems. Perceptions that species damage property, negative attitudes, and personal experience of problems were universally associated with beliefs that species cause problems generally. Other factors included, in some cases, respondents being older (pigeons, gulls, moles, squirrels), older homes (pigeons, rabbits), and storing food waste outdoors (foxes, mice, rabbits), etc. Regional issues may influence people’s beliefs about species causing problems generally; for example, people from Northern Ireland and Southern England tended more often to say that badgers cause problems, and these are both areas of the UK where badgers are or have been culled in an effort to reduce bTB in cattle [89,90]. The number of perceived traits associated with wasps being thought to cause problems generally was striking, perhaps explaining why wasps were one of the most complained about species in this survey.

#### 4.1.8. Modelling—Negative Language

We modelled perceived species traits as potential drivers of negative language (‘pest’ and/or ‘vermin’) use among people believing that a particular species causes problems for people generally in the UK. Some factors were consistently associated with negative language use. The most important (in order of decreasing importance) were species numbers being out of control, species spreading disease to people, pets, or poultry, species damaging property, and respondents being afraid of the species. Where people feel justified in using negative language about wildlife, this may provide an excuse to treat wildlife with less ethical or welfare concern [38]. Therefore, information campaigns to counter unfounded concerns could help to rationalise fears and improve the treatment of wildlife.

#### 4.1.9. Modelling—Demand for Control

Models predicting the past use of control by people who had reported pest problems revealed some predictors of low tolerance. It was striking how few factors were involved and how these varied among the four species for which models converged. Behaviours, such as conducting control measures, are how attitudes, beliefs, and values are expressed [91]. Older people and people believing that rats spread disease to pets and poultry were more likely to have attempted rat control, while people with cats or dogs, or who were afraid of rats were less likely to have done so. Older people may have more entrenched views about rats, and perceptions about risk of disease spread to valued animals is clearly a key trigger for rat control, whereas people with cats and dogs may feel that these will kill or deter rats or, being pet-lovers, they may be more inclined to tolerate rats. The UK public famously loves it’s pets, with 40% of people owning a pet, most being dogs or cats [92], and pet ownership may be an important factor in how people view and interact with wildlife in the UK. People who fear rats may not be prepared to engage with rat control. Mice were viewed positively overall, but people with negative attitudes towards them, those thinking that mice enter homes, and people owning their own home or living in rented accommodation were more likely to control them. This suggests that the perceived entry of mice into homes and home tenure can influence people’s tolerance of mice, with homeowners or renters being more inclined to attempt control or seek this from their council. Residents with gardens and those who perceive that wasps enter and damage homes, e.g., by making nests [9], may be more inclined to control wasps, while those thinking that wasps are a nuisance only around food outdoors may not bother. People in detached or semi-detached homes, keeping pets or poultry, growing their own food, or maintaining gardens to a high standard may be less inclined to tolerate mole activity because of their greater investment of time and money in these activities. The improved availability of good quality information on how to prevent rats infiltrating outdoor pet or poultry pens, feeders, and food supplies; deter mice and wasps from entering homes; and use non-lethal ways of managing perceived mole damage (e.g., molehills and surface tunnels) should improve the tolerance of these species’ presence. In addition, positive portrayal of species could improve perceptions; for example, positive representation of wasps as pollinators and natural ‘pest controllers’ could help improve public perceptions of them as has been achieved for bees [9].

#### 4.1.10. Finding Help with a Problem

Most people in our public survey said they would search the internet (73%) or go to their local council (56%) for information on dealing with a wild animal problem. A survey of pest control services in England found that 50% and 51% of residents would go to their council for information on rodents and insects respectively, with the next largest response being that they would seek information on the internet [93]. While more people in our study felt that the resident or owner of a property should be responsible for conducting and paying for ‘controlling’ an infestation, around one-third felt that their local council should be responsible. However, in a 2013 study, 64% of people in England said that responsibility for “making sure we don’t have pests” lay with the council, while 44% said the council should pay for an infestation to be dealt with, and 38% thought that the person at fault should pay [93]. This suggests that many respondents would be prepared to assume responsibility for solving a wildlife problem themselves, reflecting their strong reliance on DIY pest control for most species. Nonetheless, councils are closely linked with pest control in people’s minds, and there is likely to be an appetite among the public not only for council advice on pest control services but also advice on prevention and deterrence, which is currently not widely available.

### 4.2. Council Pest Control Services

#### 4.2.1. Council Pest Control Finances

Increased out-sourcing of public pest control by councils may be a bid to reduce council costs or a result of reduced Government funding [94]. However, it is uncertain whether using contracted-out or referral services presents a financial benefit or dis-benefit to councils. In addition, there are no data on whether service quality has declined with out-sourcing. The observed increase in councils’ revenue from public pest control may be linked to the fact that increasing numbers of UK councils are introducing charges to the public for their pest control services, scrapping subsidies or increasing existing service fees to commercial levels [95], or a result of councils increasingly opting for a referral service. It has been suggested that councils consider charging as “a way of countering the service being exploited when free and of encouraging a greater sense of responsibility on the part of householders who could do more to prevent infestations” [96]. However, introducing or raising charges could lead to a decline in public complaints to councils and to an increase in DIY treatments, potentially at the cost of increased animal welfare impacts and risks to the safety of users, non-target animals and wildlife, and the wider environment. 

High net losses from public pest control cannot necessarily be attributed to high expenditure, as costs may be recovered through service revenue. While net annual gain appears to be the result of increasing charges for public pest control, it has been argued that “the motivation [of councils] for charging was not to make a profit but simply to reduce net costs so that pest control could be kept as part of Environmental Health services in view of budget cuts” [96] and so that councils could retain their pest control services. Councils have no statutory duty to provide public pest control services, with their only legal obligations under the Prevention of Damage by Pests Act (1949) applying to rats and mice, to ensure that the area is safe from damage. Indeed, upon enquiry, several councils stated that their net annual gain from public pest control was not treated as a profit but used to help fund the delivery of pest control and other Environmental Health services.

#### 4.2.2. Council Pest Control by Species

The vast majority (79.9–88.0%) of councils that offered any public pest control offered services for rats, mice, and wasps (all three species), while rats (18.1%) and mice (10.7%) were the species for which councils most often offered free treatments. This suggests that local authorities perceive these three species as posing the greatest risk to public health and safety. Indeed, FOI requests revealed that rats, mice, and wasps were the species for which people most frequently requested treatments from the council, and this matches our YouGov^®^ public survey finding that these were the three species most frequently reported by the public as causing problems. Councils may also wish to encourage the public reporting of issues with these particular animals, potentially to avoid infestations getting out of control owing to the species’ rapid reproduction rates. The findings may also reflect councils’ recognition of their obligations regarding the management of rats and mice under the Prevention of Damage by Pests Act (1949), which requires councils “to take such steps as may be necessary to secure so far as practicable that their district is kept free from rats and mice” (Prevention of Damage by Pests Act (1949), Section 2(1)).

The increasing proportions of councils providing control services for rats, mice, wasps, squirrels, pigeons, moles, gulls, and foxes could indicate that human–wildlife conflicts involving these species are increasing. Pest control provision may previously have been inadequate, potentially allowing problems to increase or spread to other areas and forcing more councils to control animals. This highlights the need for a systematic and coordinated approach across councils to achieve sustainable long-term solutions (see [38]). Many councils do not keep separate records of the number of call-outs, treatments, and repeat treatments they undertake (Stephanie Maw, unpublished data), and this should be rectified to assist in joined-up monitoring and planning. An alternative explanation for the increase in council pest control availability could be that this is motivated by financial gain. However, some councils participating in this survey reported that pest control was not seen as financially profitable for councils, and over 85% of councils made a loss in 2016/17. 

Interestingly, gulls appeared midway down the ranking of species by the total annual number of treatment requests to councils, but they ranked only seventh in terms of the annual proportion of councils treating for them. This dichotomy mirrors that observed in our public survey. This suggests that although only a relatively small number of councils offered public pest control for gulls, these councils combined received a relatively high number of annual treatment requests. This is likely linked to councils in certain regions experiencing a higher presence of gulls, e.g., coastal areas, their traditional breeding and feeding ground, or inland urban areas, where gulls have been increasingly settling [7].

#### 4.2.3. Finding Advice on Prevention and Deterrence

Only half of councils provided information on their websites on preventing and deterring wildlife problems during 2017/18. Some councils with the greatest number of treatment requests and others that did not provide an in-house service provided no such information. Coverage of information varied in quality and detail, and some species were particularly poorly represented. For example, while wasps were the third most commonly treated species (with 90.3% of pest control providing UK councils offering wasp treatments in 2016/17), only 6.2% of all councils supplied information on how to prevent and deter wasps. 

In contrast, a much greater proportion of councils provided prevention and deterrence advice for foxes (19.7%) than offered fox treatments (7.4% in 2016/17). The low rate of council provision of fox control services could be explained by the status of the red fox as an iconic British mammal [97], as well as the public controversy surrounding fox culling, as seen over fox hunting [98]. Moreover, the vast majority of councils have come to recognise that control of fox populations, especially urban fox populations, “is difficult, expensive and not normally successful” [99]; removing foxes creates vacant territory where other foxes will quickly establish themselves, while the cost of control generally outweighs the level of nuisance caused [100]. By contrast, “Deterring foxes is cheaper, more humane and can be more effective” [99]. Several councils have published their own extensive advice on their websites on how to prevent and discourage foxes in residential areas in order to allow people and foxes to coexist with minimal conflict [101,102]. 

Providing the public with advice and information on how to prevent wildlife from entering a property or causing damage is an essential step for any council seeking to adopt a more proactive, efficient, ethical, and humane approach to managing and living with wildlife (see Table S6, [18,38]) and to provide long-term, effective solutions to its residents. Councils operating an in-house pest control service may be the most likely to provide advice on preventing and deterring unwanted wildlife, because there may be financial incentives for them to minimise pest problems, and they have trained internal staff at their disposal. Meanwhile, councils offering a contracted-out service may have lost any in-house expertise, and there may be little reason for commercial pest control companies to reduce pest problems in the long-term. However, the potential financial trap of conducting ineffective wildlife management should be reason for concern to local authorities because public pest control is funded from the public purse. 

The quality of information available on the wider internet is extremely variable, so it is important that all local council websites provide clear and comprehensive advice to the public about both preventing and solving problems with wild animals. Given the observed shift from in-house to outsourced pest control services, it is important that all councils provide such information. Drafting and updating high-quality, standardised information would probably be most efficient if done centrally and the information distributed to councils for use. The costs of producing this information should be offset by reductions in demand for control services from councils. 

Similar public-friendly information could be made available on government and other public body websites, e.g., the Department for Environment, Food and Rural Affairs (Defra) and the Chartered Institute for Environmental Health (CIEH). Advice should be kept up to date and standardised across the UK, unless there are geographically specific issues. Sound advice and reliable services would help to ensure that people use only legal, safe, and effective methods.

#### 4.2.4. Refocusing Wildlife Management

Current wildlife management practices tend to be reactive, removing animals without addressing the conditions attracting them, and cannot therefore provide a long-term solution. It would be better to focus less on the removal of animals, which probably fuels concern about the number of animals, and instead use a proactive approach, focussing on preventing unwelcome wildlife from becoming established in the first place. This would be more sustainable and ethical, e.g., reducing the vast numbers of rodents that are killed inhumanely [18], and it would concur with Dubois et al.’s [38] first principle of ethical wildlife control, to start by changing human behaviour. Indeed, anticoagulant rodenticides, the most commonly used method of rodent control, take several days to kill, causing distress, disability, and pain during this time. Preventative methods such as proofing are humane and a necessary factor in effective control [18]. For example, Canadian pest control professionals believed that while baiting is the cheapest and easiest method of rat control, environmental modification is the most effective way to control rat infestations in the long-term [12]. In addition, preventing access to human food resources, stopping the public feeding of pigeons [103,104], and avoiding the use of attractive building structures and high-rise buildings, in residential areas should reduce pigeon density [11,103]. Concerns about species spreading disease to people or animals will vary, e.g., rats may be associated with Weil’s disease (Leptospirosis) [105], pigeons with *Listeria* and *E. coli* [106], and badgers with bTB [107]. Increased education about real versus perceived or exaggerated risks (e.g., regarding the risks of disease transmission, attacks by gulls or foxes, and how to avoid these, or about natural cycles in population numbers) might help alleviate public concerns and increase tolerance.

## 5. Animal Welfare Summary

The presence of wildlife species in and around people’s homes may lead to different types of conflict and to action with animal welfare impacts. Control is often attempted, and professional control in particular (including that administered by councils) usually involves killing the animals concerned. Inhumane methods are frequently used, including anticoagulant rodenticides and poor quality, unregulated rodent and mole traps. Some species are killed on an enormous scale [18]. Non-lethal methods can also be inhumane, such as translocating live-trapped moles [33]. Removing animals without first addressing the source of the original problem (e.g., changing human behaviour or the animal’s environment) will not provide a long-term, ethical, or sustainable solution [38], because new animals are likely to replace those that have been removed and the cycle will continue. There needs to be much greater emphasis on preventing wildlife problems through proofing and deterrence, and in some cases by promoting greater tolerance, such as by managing mole ‘damage’ rather than removing moles [34]. Better information on preventing problems (such as on proofing poultry runs and avoiding dive-bombing by gulls), and when necessary resolving them, should be made available on council and other websites. Tackling myths about the risks involved with some species, such as attacks by foxes, may also be fruitful. Informing people about the benefits wildlife brings, such as pollination by wasps, may also help to improve tolerance. Councils, pest controllers and manufacturers of pest control products are missing an important opportunity by focussing on control rather than prevention.

## Figures and Tables

**Table 1:** Species ranked by positive mean attitude score and by the percentage of people reporting that the species had caused them a problem personally and the percentage believing that the species causes problems for people generally in the UK (*n* = 2000). Greater attitude scores indicate more positive attitudes.

Negative-Positive Scale	Mean Attitude Score Rank Score			Believe Cause Problem Generally		Experienced Problem Personally	
				%		%	
						**None of the**	
						**species**	**35.6**
**Most**	**1**	Rat	−1.832	Rat	81.1	Wasp	34.4
**negative**	**2**	Wasp	−1.471	Wasp	62.6	Mouse	31.1
**|**	**3**	Gull	−0.746	Mouse	59.8	Rat	21.9
**|**	**4**	Pigeon	−0.33	Pigeon	37.0	Pigeon	9.2
**|**	**5**	Mouse	0.196	Gull	35.6	Fox	8.8
**|**	**6**	Squirrel	0.488	Fox	32.8	Squirrel	6.6
**|**	**7**	Mole	1.174	Mole	32.2	Gull	6.0,
**|**	**8**	Fox	1.474	Squirrel	21.5	Mole	5.9
**Most**	**9**	Rabbit	1.776	Badger	14.9	Rabbit	2.4
**positive**	**10**	Badger	1.78	Rabbit	11.3	Badger	2.1
				**None of the**			
				**species**	**8.9**		
**Attitude vs. belief**		**R = −0.83, *p* = 0.003**					
**Belief vs. experience**				**R = 0.85, *p* = 0.002**			
**Attitude vs. experience**		**R = −0.66, *p* = 0.038**					

**Table 2:** Importance ranking of factors in choosing Do-It-Yourself (DIY) and professional (Pro.) control services for 10 species. Scores shown are mean importance scores; positive scores indicate that a factor is considered important and greater scores indicate greater importance. Q = ’Solves problem quickly’, L = ’Long-lasting effect’, C = ’Low cost’, NL = ’Non-lethal (does not involve killing)’, W = ’Welfare-friendly for the target pest, E = ’Easy to use’, H = ’Hygienic (requires me to have minimal contact with dead animal)’, SP = ’Safe for people and pets’, SN = ’Safe for non-target wild animals’. The four most important factors (ranked highest) for each species are shown in bold.

**DIY Badger**		**DIY Fox**		**DIY Mole**		**DIY Mouse**		**DIY Pigeon**		**DIY Rabbit**		**DIY Rat**		**DIY Gull**		**DIY Squirrel**		**DIY Wasp**	
***n* = 12**		***n* = 67**		***n* = 54**		***n* = 492**		***n* = 101**		***n* = 25**		***n* = 255**		***n* = 45**		***n* = 74**		***n* = 355**	
**L**	**1.58**	**SN**	**1.61**	**SN**	**1.69**	**Q**	**1.65**	**L**	**1.54**	**SP**	**1.60**	**Q**	**1.75**	**SP**	**1.44**	**SP**	**1.68**	**Q**	**1.82**
**SP**	**1.58**	**SP**	**1.60**	**SP**	**1.56**	**SP**	**1.59**	**SN**	**1.50**	**SN**	**1.48**	**L**	**1.67**	**L**	**1.38**	**L**	**1.64**	**SP**	**1.63**
**SN**	**1.5**	**L**	**1.58**	**L**	**1.39**	**SN**	**1.45**	**SP**	**1.47**	**L**	**1.32**	**SP**	**1.54**	**Q**	**1.29**	**Q**	**1.58**	**L**	**1.56**
**Q**	**1.42**	**Q**	**1.52**	**Q**	**1.35**	**L**	**1.45**	**Q**	**1.44**	**Q**	**1.24**	**SN**	**1.49**	**SN**	**1.24**	**SN**	**1.57**	**SN**	**1.55**
E	1.42	E	1.39	E	1.33	E	1.42	E	1.26	E	1.16	E	1.35	E	0.98	E	1.35	E	1.45
H	1.25	H	1.15	C	0.78	H	0.99	C	0.96	C	0.72	H	1.02	H	0.93	C	1.05	C	0.94
C	1.17	C	0.75	H	0.52	C	0.95	H	0.95	H	0.44	C	0.94	C	0.91	H	0.86	H	0.92
W	0.83	W	0.57	W	0.33	W	0.29	W	0.10	W	−0.12	W	−0.26	NL	0.02	W	−0.12	W	−0.43
NL	0.58	NL	0.40	NL	−0.09	NL	−0.04	NL	0.01	NL	−0.28	NL	−0.70	W	0.00	NL	−0.32	NL	−0.68
**Pro. Badger**		**Pro. Fox**		**Pro. Mole**		**Pro. Mouse**		**Pro. Pigeon**		**Pro. Rabbit**		**Pro. Rat**		**Pro. Gull**		**Pro. Squirrel**		**Pro. Wasp**	
***n* = 10**		***n* = 54**		***n* = 37**		***n* = 141**		***n* = 56**		***n* = 13**		***n* = 230**		***n* = 30**		***n* = 44**		***n* = 400**	
**Q**	**1.40**	**SP**	**1.63**	**Q**	**1.51**	**L**	**1.83**	**L**	**1.71**	**Q**	**1.31**	**Q**	**1.82**	**L**	**1.73**	**Q**	**1.80**	**Q**	**1.87**
**L**	**1.40**	**L**	**1.59**	**L**	**1.51**	**Q**	**1.77**	**Q**	**1.55**	**L**	**1.23**	**L**	**1.78**	**Q**	**1.60**	**L**	**1.77**	**L**	**1.78**
**SN**	**1.30**	**SN**	**1.56**	**SP**	**1.46**	**SP**	**1.58**	**SP**	**1.55**	**SP**	**1.08**	**SP**	**1.69**	**SN**	**1.40**	**SP**	**1.77**	**SP**	**1.65**
NL	1.10	Q	1.48	SN	1.35	SN	1.40	SN	1.52	SN	0.92	SN	1.53	SP	1.37	SN	1.68	SN	1.53
C	1.00	E	0.89	C	0.92	H	1.20	E	1.04	E	0.54	H	1.24	H	1.13	H	1.27	E	0.99
W	1.00	C	0.80	E	0.86	E	1.10	C	0.98	C	0.31	E	1.11	E	0.80	E	1.05	H	0.98
**SP**	**1.00**	H	0.70	H	0.59	C	0.96	H	0.96	H	0.31	C	0.87	C	0.77	C	0.91	C	0.84
H	0.80	W	0.56	W	0.27	W	0.03	W	0.11	W	−0.31	W	−0.22	W	0.07	W	0.16	W	−0.46
E	0.70	NL	0.26	NL	0.08	NL	−0.25	NL	−0.02	NL	−0.38	NL	−0.55	NL	−0.03	NL	−0.11	NL	−0.70

**Table 3:** Mean importance scores showing the importance in choosing DIY/professional control and of control being non-lethal and welfare-friendly for 10 species. Imp score = Importance score, DIY = Do-It-Yourself control products, Pro. = professional control services. Positive importance scores (shown yellow) indicate that a factor is considered important in choosing control, and greater scores indicate greater importance. Species are ranked in order of decreasing importance of welfare-friendliness or non-lethality.

Non-Lethal						Welfare-Friendly					
DIY	Imp Score	*n*	Pro.	Imp Score	*n*	DIY	Imp Score	*n*	Pro.	Imp Score	*n*
Badger	0.58	12	Badger	1.10	10	Badger	0.83	12	Badger	1.00	10
Fox	0.40	67	Fox	0.26	54	Fox	0.57	67	Fox	0.56	54
Gull	0.02	45	Mole	0.08	37	Mole	0.33	54	Mole	0.27	37
Pigeon	0.01	101	Pigeon	−0.02	56	Mouse	0.29	492	Squirrel	0.16	44
Mouse	−0.04	492	Gull	−0.03	30	Pigeon	0.10	101	Pigeon	0.11	56
Mole	−0.09	54	Squirrel	−0.11	44	Gull	0.00	45	Gull	0.07	30
Rabbit	−0.28	25	Mouse	−0.25	141	Rabbit	−0.12	25	Mouse	0.03	141
Squirrel	−0.32	74	Rabbit	−0.38	13	Squirrel	−0.12	74	Rat	−0.22	230
Wasp	−0.68	355	Rat	−0.55	230	Rat	−0.26	255	Rabbit	−0.31	13
Rat	−0.70	255	Wasp	−0.70	400	Wasp	−0.43	355	Wasp	−0.46	400

**Table 4:** Factors associated with people’s negative attitudes towards eight species (*n* = 1828). Factors are grouped under: Demog. = ‘Demography’, Home = ‘Home’, Human behav. = ‘Human behaviour’, Perc. spp. traits = ‘Perceived species traits’, and Perc. about spp. = ‘Perceptions about species’. Factors shown in red were associated with a more positive attitude, those shown in green were associated with a more negative attitude, and those shown yellow were associated with a more complex effect. See list of abbreviations for factors in Supplementary Materials Table S2.

Item	Badger	Fox	Mole	Mouse	Pigeon	Rabbit	Rat	Gull	Squirrel	Wasp
Demog.		X				Gender (M)			X	Gender (M)
		X	Increasing age	Increasing age		Increasing age	Increasing age		X	
		X							X	Social grade (work. class)
		X							X	
		X							X	
Home		X	Home type						X	
	Older home	X	Older home	Older home	Older home		Older home	Older home	X	Older home
		X		Home tenure	Home tenure	Home tenure	Home tenure	Home tenure	X	
Human behav.	Children	X		Children					X	
		X							X	
	Cats / dogs	X	Cats / dogs	Cats / dogs	Cats / dogs	Cats / dogs	Cats / dogs	Cats / dogs	X	
		X							X	
		X	Feedstore	Feedstore					X	
	Feed birds / animals	X	Feed birds / animals	Feed birds / animals	Feed birds / animals	Feed birds / animals		Feed birds / animals	X	Feed birds / animals
	Compost	X		Compost					X	
		X							X	
Perc. Spp. traits		X							X	
		X							X	
	Disease to people	X	Disease to people	Disease to people	Disease to people	Disease to people	Disease to people	Disease to people	X	Disease to people
		X				Attack people	Attack people	Attack people	X	Attack people
	Disease to pets / poul	X		Disease to pets / poul	Disease to pets / poul		Disease to pets / poul	Disease to pets / poul	X	
		X		Attack pets /poul				Attack pets /poul	X	Attack pets /poul
	Damage	X	Damage			Damage			X	Damage
	Numbers	X	Numbers	Numbers	Numbers	Numbers	Numbers	Numbers	X	Numbers
	Fear	X	Fear	Fear	Fear	Fear	Fear	Fear	X	Fear
Perc. about spp.		X	Experience	Experience	Experience	Experience	Experience	Experience	X	Experience
	Belief	X	Belief	Belief	Belief	Belief	Belief	Belief	X	Belief

**Table 5:** Factors associated with people’s personal experience of problems with eight species (*n* = 1828). Factors are grouped under: Demog. = ‘Demography’, Home = ‘Home’, Human behav. = ‘Human behaviour’, Perc. spp. traits = ‘Perceived species traits’, and Perc. about spp. = ‘Perceptions about species’. Factors shown in red were associated with a lower likelihood of experiencing problems, those shown in green were associated with a greater likelihood, and those shown in yellow were associated with a more complex effect. See the list of abbreviations for factors in Supplementary Materials Table S2.

Item	Badger	Fox	Mole	Mouse	Pigeon	Rabbit	Rat	Gull	Squirrel	Wasp
Demog.					Gender (M)	Gender (M)		Gender (M)	X	X
	Increasing age	Increasing age		Increasing age	Increasing age		Increasing age		X	X
									X	X
			Urban	Urban		Urban	Urban / fringe		X	X
		Region		Region			Region	Region	X	X
Home						Home type			X	X
	Older home	Older home		Older home		Older home	Older home		X	X
									X	X
Human behav.									X	X
								Garden	X	X
									X	X
							Poultry / OD pets		X	X
	Feedstore	Feedstore	Feedstore	Feedstore			Feedstore		X	X
									X	X
		Compost	Compost		Compost		Compost		X	X
									X	X
Perc. Spp. traits									X	X
				Enter homes					X	X
	Disease to people			Disease to people					X	X
									X	X
									X	X
									X	X
	Damage	Damage	Damage	Damage	Damage		Damage	Damage	X	X
		Numbers							X	X
									X	X
Perc. about spp.	Belief	Belief	Belief	Belief	Belief	Belief	Belief	Belief	X	X
		Neg attitude	Neg attitude	Neg attitude	Neg attitude	Neg attitude	Neg attitude	Neg attitude	X	X

**Table 6:** Factors associated with people’s beliefs about 10 species causing problems for people generally in the UK (*n* = 1828). Factors are grouped under: Demog. = ‘Demography’, Home = ‘Home’, Human behav. = ‘Human behaviour’, Perc. spp. traits = ‘Perceived species traits’, and Perc. about spp. = ‘Perceptions about species’. Factors shown in red were associated with a lower likelihood of believing species cause problems generally, those shown in green were associated with a greater likelihood, and those shown in yellow were associated with a more complex effect. See the list of abbreviations for factors in Supplementary Materials Table S2.

Item	Badger	Fox	Mole	Mouse	Pigeon	Rabbit	Rat	Gull	Squirrel	Wasp
Demog.										
			Increasing age		Increasing age			Increasing age	Increasing age	
									
		Urban								
	Region							Region		
Home										
					Older home	Older home				
									
Human behav.										
									Garden	
									
									
									
									
									
		Food waste		Food waste		Food waste				
Perc. Spp. traits		Eat our food		Eat our food		Eat our food		Eat our food	Eat our food	
		Enter homes		Enter homes			Enter homes		Enter homes	Enter homes
					Disease to people		Disease to people			
						Attack people		Attack people		Attack people
	Disease to pets / poul	Disease to pets / poul								Disease to pets / poul
		Attack pets / poul								Attack pets / poul
	Damage	Damage	Damage	Damage	Damage	Damage	Damage	Damage	Damage	Damage
	Numbers	Numbers			Numbers		Numbers	Numbers	Numbers	Numbers
										Fear
Perc. about spp.	Experience	Experience	Experience	Experience	Experience	Experience	Experience	Experience	Experience	Experience
	Neg attitude	Neg attitude	Neg attitude	Neg attitude	Neg attitude	Neg attitude	Neg attitude	Neg attitude	Neg attitude	Neg attitude

**Table 7:** Factors associated with people using negative language (‘pest’ or ‘vermin’) to refer to each of 10 species, when they believed that the particular species causes problems for people generally in the UK. Factors are all Perc. spp. traits = ‘Perceived species traits’. Factors shown in green were associated with a greater likelihood of using negative language. See the list of abbreviations for factors in Supplementary Materials Table S2.

Item	Badger	Fox	Mole	Mouse	Pigeon	Rabbit	Rat	Gull	Squirrel	Wasp
	*n* = 298	*n* = 655	*n* = 645	*n* = 1195	*n* = 741	*n* = 226	*n* = 1622	*n* = 713	*n* = 430	*n* = 1253
Perc. Spp. traits			Eat our food					Eat our food		
									
	Disease to people	Disease to people		Disease to people	Disease to people	Disease to people	Disease to people	Disease to people		Disease to people
	Attack people	Attack people								
	Disease to pets / poul	Disease to pets / poul		Disease to pets / poul	Disease to pets / poul		Disease to pets / poul	Disease to pets / poul	Disease to pets / poul	
										Attack pets / poul
		Damage	Damage	Damage	Damage	Damage	Damage		Damage	
	Numbers	Numbers	Numbers	Numbers	Numbers	Numbers	Numbers	Numbers	Numbers	Numbers
			Fear	Fear	Fear		Fear			Fear

**Table 8:** Factors associated with people’s past control of each of four species when they had experienced problems personally with that species. Factors are grouped under: Demog. = ‘Demography’, Home = ‘Home’, Human behav. = ‘Human behaviour’, Perc. spp. traits = ‘Perceived species traits’, and Perc. about spp. = ‘Perceptions about species’. Factors shown in red were associated with a lower likelihood of having used control, those shown in green were associated with a greater likelihood, and those shown in yellow were associated with a more complex effect. See list of abbreviations for factors in Supplementary Materials Table S2.

Item	Badger	Fox	Mole	Mouse	Pigeon	Rabbit	Rat	Gull	Squirrel	Wasp
			*n* = 115	*n* = 658			*n* = 411			*n* = 649
Demog.	X	X			X	X		X	X	
	X	X			X	X	Increasing age	X	X	
	X	X	Social grade (work. class)		X	X		X	X	
	X	X			X	X		X	X	
	X	X			X	X		X	X	
Home	X	X	Home type		X	X		X	X	Home type
	X	X			X	X		X	X	
	X	X		Home tenure	X	X		X	X	
Human behav.	X	X			X	X		X	X	
	X	X			X	X		X	X	Garden
	X	X			X	X	Cats / dogs	X	X	
	X	X			X	X		X	X	
	X	X			X	X		X	X	
	X	X			X	X		X	X	
	X	X			X	X		X	X	
	X	X			X	X		X	X	
Perc. Spp. traits	X	X			X	X		X	X	Eat our food
	X	X		Enter homes	X	X		X	X	Enter homes
	X	X			X	X		X	X	
	X	X			X	X		X	X	
	X	X			X	X	Disease to pets / poul	X	X	
	X	X			X	X		X	X	
	X	X			X	X		X	X	Damage
	X	X			X	X		X	X	
	X	X			X	X	Fear	X	X	
Perc. about spp.	X	X			X	X		X	X	
	X	X	Neg attitude	Neg attitude	X	X		X	X	

## Supplementary Materials

The following are available online at https://www.mdpi.com/2076-2615/10/2/222/s1, Document S1: Questionnaire, Document S2: Model summaries attitudes, Document S3: Model summaries experience of problems, Document S4: Model summaries belief in general problems, Document S5: Model summaries negative language, Document S6: Model summaries past control, Table S1: Government Office Regions, Table S2: Variable abbreviations, Table S3: Council net losses, Table S4: Total council treatment requests, Table S5: Mean council treatment requests per 1000 households, Table S6: Ethical wildlife management infographic, Figure S1: Respondent home type, Figure S2: Respondent home age, Figure S3: Respondent home tenure, Figure S4: Respondent behaviours, Figure S5: Respondent attitudes, Figure S6: Use of negative language, Figure S7: Traits by species, Figure S8: Species by trait, Figure S9: Drivers of attitudes, Figure S10: Drivers of experience of problems, Figure S11: Drivers of belief in general problems, Figure S12: Drivers of negative language use, Figure S13: Drivers of past control, Figure S14: Pest control service types, Figure S15: Council expenditure and revenue, Figure S16: Councils making losses and gains, Figure S17: Councils offering free treatments, Figure S18: Councils providing prevention/deterrence advice.
