# Erratum: Baker, S.E.; Maw, S.A.; Johnson, P.J.; Macdonald, D.W. Not in My Backyard: Public Perceptions of Wildlife and ‘Pest Control’ in and around UK Homes, and Local Authority ‘Pest Control’. *Animals* 2020, *10*, 222

**DOI:** 10.3390/ani10040644

**Published:** 2020-04-08

**Authors:** Sandra E. Baker, Stephanie A. Maw, Paul J. Johnson, David W. Macdonald

**Affiliations:** 1 Wildlife Conservation Research Unit, Department of Zoology, University of Oxford, Recanati-Kaplan Centre, Tubney House, Abingdon Road, Tubney, Abingdon OX13 5QL, UK; paul.johnson@zoo.ox.ac.uk (P.J.J.); david.macdonald@zoo.ox.ac.uk (D.W.M.); 2 Campaigns Consultant to Humane Society International UK, 5 Underwood St, Hoxton, London N1 7LY, UK; smaw@hsi.org

The authors wish to make the following erratum to their paper [1]:

The Funding information has now been corrected (see the corrected version below):
