# Supplementary material for: Not in My Backyard: Public Perceptions of Wildlife and ‘Pest Control’ in and around UK Homes, and Local Authority ‘Pest Control’
Source: Animals (Basel). 2020 Jan 30;10(2):222. doi: 10.3390/ani10020222 (PMC7071040; doi:10.3390/ani10020222)

Supplementary Information

Document S1. Code for front page and questionnaire used to gather public survey data via YouGov.

```
{questionnaire UK5113406_Oxford_Pest widget_all_required=HARD nav_back=0 hide_progress=0}

{page p_information_page}

<<style>
.intro_heading{display:block;color:#1F497D;font-weight:bold;}
.consent_text{color:#000080;font-size:0.9em; font-weight:bold;}
</style>>

<<p><span class="intro_heading">General Information</span>
We appreciate your interest in participating in this survey. Please read through these terms before agreeing to participate by ticking the ‘yes’ box below.</p>

<p>We, the University of Oxford, in collaboration with Humane Society International-UK, are investigating how people feel about wild animals in their homes and gardens, and about controlling wild animals in these situations.</p>

<p>You will be asked about your opinions and experiences of ten wildlife species and your most likely actions if you were experiencing problems with these. The survey should take about 5-10 minutes. No background knowledge is required. Your answers will help efforts to reduce problems with wild animals, and will provide insights for developing better pest control products and services.</p>

<p><span class="intro_heading">How will your data be used?</span>
Your answers will be completely anonymous, and we will use all reasonable endeavours to keep them confidential. Your IP address will not be collected. Your participation is voluntary. You may withdraw at any point during the survey, before submitting your answers, by closing the browser. Only complete responses will be stored.</p>

<p>Your anonymised responses will be passed from YouGov to the University of Oxford, where they will be securely stored and analysed for use in academic publications. You are welcome to request a copy of the results in due course, using the contact details provided below.</p>

<p><span class="intro_heading">Who will have access to your data?</span>
The University of Oxford is the data controller for the purposes of the Data Protection Act 1998. Sometimes study data need to be submitted to academic journals in support of research papers, or to be made publicly available, but your responses will always remain anonymous.</p>

<p>The principal researcher on this project is Dr Sandra Baker, who is a member of the Wildlife Conservation Research Unit (WildCRU), in the Department of Zoology at the University of Oxford.</p>

<p>This project has been reviewed by, and received ethics clearance through, the University of Oxford Central University Research Ethics Committee [reference number R53946/001].</p>
```

<p>What if there is a problem?
If you have a concern about any aspect of this project, please contact Dr Sandra Baker at pestsurvey@zoo.ox.ac.uk who will do her best to answer your query. Dr Baker should acknowledge your concern within 10 working days and give you an indication of how she intends to deal with it. If you remain unhappy or wish to make a formal complaint, please contact the relevant Chair of the Research Ethics Committee at the University of Oxford:</p>

<p>Chair, Social Sciences and Humanities Inter-Divisional Research Ethics Committee; Email: ethics@socsci.ox.ac.uk; Address: Research Services, University of Oxford, Wellington Square, Oxford OX1 2JD.</p>

<p>The Chair will seek to resolve the matter in a reasonably expeditious manner.</p>>

[consent_box1] {multiple required=HARD required_text="Please tick the box below to continue"} <<span class="consent_text"> Age 18 or older>
<1/"consent"> <<span class="consent_text">Please note that you may only participate in this survey if you are 18 years of age or over. Please tick the box to certify that you are 18 or older.>

[consent_box2] {multiple required=HARD required_text="Please tick the box below to continue"} <<span class="consent_text"> Consent>
<1/"consent"> <<span class="consent_text">If you have read the information above and agree to participate with the understanding that the data (including any personal data) you submit will be processed accordingly, please tick the box to get started.>

{end page p_information_page}

{page intro}

We would like to know how you feel about wild animals and their control. Throughout, where the survey mentions your **‘property’**, this refers to **your (main) home and garden (or allotment if you have one) and their contents (your personal property)**. ‘Pest control’ refers to either killing animals or managing them non-lethally, e.g. by moving them, or using a bird scarer, etc.

{end page intro}

{page section_intro}

[q16] {grid} In general, how do you feel about each of these species? Please tick one for each species.
-[q16_1] Badger
-[q16_2] Fox
-[q16_3] Mole
-[q16_4] Mouse
-[q16_5] Pigeon
-[q16_6] Rabbit
-[q16_7] Rat
-[q16_8] Gull (including seagull)
-[q16_9] Grey squirrel
-[q16_10] Wasp

<1> Very positive
<2> Somewhat positive
<3> Neutral
<4> Somewhat negative
<5> Very negative

[q3] {multiple} Do you believe any of these species **cause problems for people/their household/property in the UK**? Please tick all the species that apply, or tick ‘None of the above’.
<1> Badger
<2> Fox
<3> Mole
<4> Mouse
<5> Pigeon
<6> Rabbit
<7> Rat
<8> Gull (including seagull)
<9> Grey squirrel
<10> Wasp
<99 xor> None of the above

{end page section_intro}

#those who believe at least one of the species in q3 to provide a threat
[q4a if not 99 in q3] {grid} Which of these terms best fits your perception of each of these species in the UK, **“pest”**, **“vermin”**, **“pest and vermin”** or *“neither pest nor vermin”**? There are no right or wrong answers here. Please tick one of the following for each species.
-[q4a_1 if 1 in q3] Badger
-[q4a_2 if 2 in q3] Fox
-[q4a_3 if 3 in q3] Mole
-[q4a_4 if 4 in q3] Mouse
-[q4a_5 if 5 in q3] Pigeon
-[q4a_6 if 6 in q3] Rabbit
-[q4a_7 if 7 in q3] Rat
-[q4a_8 if 8 in q3] Gull (including seagull)
-[q4a_9 if 9 in q3] Grey squirrel
-[q4a_10 if 10 in q3] Wasp

<1> Pest
<2> Vermin
<3> Pest and vermin
<4> Neither pest nor vermin

[q5] {multiple} Have any of these species **ever caused a problem to you/your household/property?** Please tick all the species that apply, or tick ‘None of the above’.
<1> Badger
<2> Fox
<3> Mole
<4> Mouse
<5> Pigeon
<6> Rabbit

<7> Rat
<8> Gull (including seagull)
<9> Grey squirrel
<10> Wasp
<99 xor> None of the above

#only those who have experienced a problem in the past
[q5b if not 99 in q5] {grid} In the past, when you experienced a problem with each of these species on your property, did you **buy and use a DIY pest control product** or **employ a professional pest control service** to treat the problem on your property? Please tick one of the following for each species.
-[q5b_1 if 1 in q5] Badger
-[q5b_2 if 2 in q5] Fox
-[q5b_3 if 3 in q5] Mole
-[q5b_4 if 4 in q5] Mouse
-[q5b_5 if 5 in q5] Pigeon
-[q5b_6 if 6 in q5] Rabbit
-[q5b_7 if 7 in q5] Rat
-[q5b_8 if 8 in q5] Gull (including seagull)
-[q5b_9 if 9 in q5] Grey squirrel
-[q5b_10 if 10 in q5] Wasp

<1> Bought and used a DIY pest control product only
<2> Employed a professional pest control service only
<3> Have both bought and used a DIY product and employed a professional pest control service
<99> Have done neither

#only those who have experienced a problem in the past
[q8a if not 99 in q5] {grid} In the future, if you experienced a further problem with each of these species on your property, would you be most likely to consider **buying a DIY pest control product** or **employing a professional pest control service** to treat the problem on your property? Please tick one of the following for each species.
-[q8a_1 if 1 in q5] Badger
-[q8a_2 if 2 in q5] Fox
-[q8a_3 if 3 in q5] Mole
-[q8a_4 if 4 in q5] Mouse
-[q8a_5 if 5 in q5] Pigeon
-[q8a_6 if 6 in q5] Rabbit
-[q8a_7 if 7 in q5] Rat
-[q8a_8 if 8 in q5] Gull (including seagull)
-[q8a_9 if 9 in q5] Grey squirrel
-[q8a_10 if 10 in q5] Wasp

<1> Most likely to consider buying a DIY pest control product
<2> Most likely to consider employing a professional pest control service
<3> Equally likely to consider choosing either
<99> Would do neither

[q9a if q8a_1 in [1,3]] {grid} If you were going to **buy and use a DIY Badger pest control product** for you or your family to use on your property, how important would you consider each of the factors below in making your choice of product?

-[q9a_1] Solves problem quickly
-[q9a_2] Long lasting effect
-[q9a_3] Low cost
-[q9a_4] Non-lethal (does not involve killing)
-[q9a_5] Welfare-friendly for the target pest
-[q9a_6] Easy to use
-[q9a_7] Hygienic (requires me to have minimal contact with dead animal)
-[q9a_8] Safe for people and pets
-[q9a_9] Safe for non-target wild animals

<1> Very important
<2> Somewhat important
<3> Neutral
<4> Not very important
<5> Not at all important

[q11a if q8a_1 in [2,3]] {grid} If you were going to **employ a professional Badger pest control service** to treat a problem on your property, how important would you consider each of the factors below in making your choice of professional service?
-[q11a_1] Solves problem quickly
-[q11a_2] Long lasting effect
-[q11a_3] Low cost
-[q11a_4] Non-lethal (does not involve killing)
-[q11a_5] Welfare-friendly for the target pest
-[q11a_6] Easy to use
-[q11a_7] Hygienic (requires the user to have minimal contact with dead animal)
-[q11a_8] Safe for people and pets
-[q11a_9] Safe for non-target wild animals

<1> Very important
<2> Somewhat important
<3> Neutral
<4> Not very important
<5> Not at all important

[q9b if q8a_2 in [1,3]] {grid} If you were going to **buy and use a DIY Fox pest control product** for you or your family to use on your property, how important would you consider each of the factors below in making your choice of product?
-[q9b_1] Solves problem quickly
-[q9b_2] Long lasting effect
-[q9b_3] Low cost
-[q9b_4] Non-lethal (does not involve killing)
-[q9b_5] Welfare-friendly for the target pest
-[q9b_6] Easy to use
-[q9b_7] Hygienic (requires me to have minimal contact with dead animal)
-[q9b_8] Safe for people and pets
-[q9b_9] Safe for non-target wild animals

<1> Very important
<2> Somewhat important

<3> Neutral
<4> Not very important
<5> Not at all important

[q11b if q8a_2 in [2,3]] {grid} If you were going to **employ and use a professional Fox pest control service** to treat a problem on your property, how important would you consider each of the factors below in making your choice of professional service?
-[q11b_1] Solves problem quickly
-[q11b_2] Long lasting effect
-[q11b_3] Low cost
-[q11b_4] Non-lethal (does not involve killing)
-[q11b_5] Welfare-friendly for the target pest
-[q11b_6] Easy to use
-[q11b_7] Hygienic (requires the user to have minimal contact with dead animal)
-[q11b_8] Safe for people and pets
-[q11b_9] Safe for non-target wild animals

<1> Very important
<2> Somewhat important
<3> Neutral
<4> Not very important
<5> Not at all important

[q9c if q8a_3 in [1,3]] {grid} If you were going to **buy and use a DIY Mole pest control product** for you or your family to use on your property, how important would you consider each of the factors below in making your choice of product?
-[q9c_1] Solves problem quickly
-[q9c_2] Long lasting effect
-[q9c_3] Low cost
-[q9c_4] Non-lethal (does not involve killing)
-[q9c_5] Welfare-friendly for the target pest
-[q9c_6] Easy to use
-[q9c_7] Hygienic (requires me to have minimal contact with dead animal)
-[q9c_8] Safe for people and pets
-[q9c_9] Safe for non-target wild animals

<1> Very important
<2> Somewhat important
<3> Neutral
<4> Not very important
<5> Not at all important

[q11c if q8a_3 in [2,3]] {grid} If you were going to **employ and use a professional Mole pest control service** to treat a problem on your property, how important would you consider each of the factors below in making your choice of professional service?
-[q11c_1] Solves problem quickly
-[q11c_2] Long lasting effect
-[q11c_3] Low cost
-[q11c_4] Non-lethal (does not involve killing)
-[q11c_5] Welfare-friendly for the target pest

-[q11c_6] Easy to use
-[q11c_7] Hygienic (requires the user to have minimal contact with dead animal)
-[q11c_8] Safe for people and pets
-[q11c_9] Safe for non-target wild animals

<1> Very important
<2> Somewhat important
<3> Neutral
<4> Not very important
<5> Not at all important

[q9d if q8a_4 in [1,3]] {grid} If you were going to **buy and use a DIY Mouse pest control product** for you or your family to use on your property, how important would you consider each of the factors below in making your choice of product?
-[q9d_1] Solves problem quickly
-[q9d_2] Long lasting effect
-[q9d_3] Low cost
-[q9d_4] Non-lethal (does not involve killing)
-[q9d_5] Welfare-friendly for the target pest
-[q9d_6] Easy to use
-[q9d_7] Hygienic (requires the user to have minimal contact with dead animal)
-[q9d_8] Safe for people and pets
-[q9d_9] Safe for non-target wild animals

<1> Very important
<2> Somewhat important
<3> Neutral
<4> Not very important
<5> Not at all important

[q11d if q8a_4 in [2,3]] {grid} If you were going to **employ a professional Mouse pest control service** to treat a problem on your property, how important would you consider each of the factors below in making your choice of professional service?
-[q11d_1] Solves problem quickly
-[q11d_2] Long lasting effect
-[q11d_3] Low cost
-[q11d_4] Non-lethal (does not involve killing)
-[q11d_5] Welfare-friendly for the target pest
-[q11d_6] Easy to use
-[q11d_7] Hygienic (requires the user to have minimal contact with dead animal)
-[q11d_8] Safe for people and pets
-[q11d_9] Safe for non-target wild animals

<1> Very important
<2> Somewhat important
<3> Neutral
<4> Not very important
<5> Not at all important

[q9e if q8a_5 in [1,3]] {grid} If you were going to **buy and use a DIY Pigeon pest control product** for you or your family to use on your property, how important would you consider each of the factors below in making your choice of product?
-[q9e_1] Solves problem quickly
-[q9e_2] Long lasting effect
-[q9e_3] Low cost
-[q9e_4] Non-lethal (does not involve killing)
-[q9e_5] Welfare-friendly for the target pest
-[q9e_6] Easy to use
-[q9e_7] Hygienic (requires me to have minimal contact with dead animal)
-[q9e_8] Safe for people and pets
-[q9e_9] Safe for non-target wild animals

<1> Very important
<2> Somewhat important
<3> Neutral
<4> Not very important
<5> Not at all important

[q11e if q8a_5 in [2,3]] {grid} If you were going to **employ a professional Pigeon pest control service** to treat a problem on your property, how important would you consider each of the factors below in making your choice of professional service?
-[q11e_1] Solves problem quickly
-[q11e_2] Long lasting effect
-[q11e_3] Low cost
-[q11e_4] Non-lethal (does not involve killing)
-[q11e_5] Welfare-friendly for the target pest
-[q11e_6] Easy to use
-[q11e_7] Hygienic (requires the user to have minimal contact with dead animal)
-[q11e_8] Safe for people and pets
-[q11e_9] Safe for non-target wild animals

<1> Very important
<2> Somewhat important
<3> Neutral
<4> Not very important
<5> Not at all important

[q9f if q8a_6 in [1,3]] {grid} If you were going to **buy and use a DIY Rabbit pest control product** for you or your family to use on your property, how important would you consider each of the factors below in making your choice of product?
-[q9f_1] Solves problem quickly
-[q9f_2] Long lasting effect
-[q9f_3] Low cost
-[q9f_4] Non-lethal (does not involve killing)
-[q9f_5] Welfare-friendly for the target pest
-[q9f_6] Easy to use
-[q9f_7] Hygienic (requires me to have minimal contact with dead animal)
-[q9f_8] Safe for people and pets
-[q9f_9] Safe for non-target wild animals

<1> Very important
<2> Somewhat important
<3> Neutral
<4> Not very important
<5> Not at all important

[q11f if q8a_6 in [2,3]] {grid} If you were going to **employ a professional Rabbit pest control service** to treat a problem on your property, how important would you consider each of the factors below in making your choice of professional service?
-[q11f_1] Solves problem quickly
-[q11f_2] Long lasting effect
-[q11f_3] Low cost
-[q11f_4] Non-lethal (does not involve killing)
-[q11f_5] Welfare-friendly for the target pest
-[q11f_6] Easy to use
-[q11f_7] Hygienic (requires the user to have minimal contact with dead animal)
-[q11f_8] Safe for people and pets
-[q11f_9] Safe for non-target wild animals

<1> Very important
<2> Somewhat important
<3> Neutral
<4> Not very important
<5> Not at all important

[q9g if q8a_7 in [1,3]] {grid} If you were going to **buy and use DIY Rat pest control product** for you or your family to use on your property, how important would you consider each of the factors below in making your choice of product?
-[q9g_1] Solves problem quickly
-[q9g_2] Long lasting effect
-[q9g_3] Low cost
-[q9g_4] Non-lethal (does not involve killing)
-[q9g_5] Welfare-friendly for the target pest
-[q9g_6] Easy to use
-[q9g_7] Hygienic (requires me to have minimal contact with dead animal)
-[q9g_8] Safe for people and pets
-[q9g_9] Safe for non-target wild animals

<1> Very important
<2> Somewhat important
<3> Neutral
<4> Not very important
<5> Not at all important

[q11g if q8a_7 in [2,3]] {grid} If you were going to **employ a professional Rat pest control service** to treat a problem on your property, how important would you consider each of the factors below in making your choice of professional service?
-[q11g_1] Solves problem quickly
-[q11g_2] Long lasting effect

-[q11g_3] Low cost
-[q11g_4] Non-lethal (does not involve killing)
-[q11g_5] Welfare-friendly for the target pest
-[q11g_6] Easy to use
-[q11g_7] Hygienic (requires the user to have minimal contact with dead animal)
-[q11g_8] Safe for people and pets
-[q11g_9] Safe for non-target wild animals

<1> Very important
<2> Somewhat important
<3> Neutral
<4> Not very important
<5> Not at all important

[q9h if q8a_8 in [1,3]] {grid} If you were going to **buy and use a DIY Gull (including seagull) pest control product** for you or your family to use on your property, how important would you consider each of the factors below in making your choice of product?
-[q9h_1] Solves problem quickly
-[q9h_2] Long lasting effect
-[q9h_3] Low cost
-[q9h_4] Non-lethal (does not involve killing)
-[q9h_5] Welfare-friendly for the target pest
-[q9h_6] Easy to use
-[q9h_7] Hygienic (requires me to have minimal contact with dead animal)
-[q9h_8] Safe for people and pets
-[q9h_9] Safe for non-target wild animals

<1> Very important
<2> Somewhat important
<3> Neutral
<4> Not very important
<5> Not at all important

[q11h if q8a_8 in [2,3]] {grid} If you were going to **employ a professional Gull (including seagull) pest control service** to treat a problem on your property, how important would you consider each of the factors below in making your choice of professional service?
-[q11h_1] Solves problem quickly
-[q11h_2] Long lasting effect
-[q11h_3] Low cost
-[q11h_4] Non-lethal (does not involve killing)
-[q11h_5] Welfare-friendly for the target pest
-[q11h_6] Easy to use
-[q11h_7] Hygienic (requires the user to have minimal contact with dead animal)
-[q11h_8] Safe for people and pets
-[q11h_9] Safe for non-target wild animals

<1> Very important
<2> Somewhat important
<3> Neutral
<4> Not very important

<5> Not at all important

[q9i if q8a_9 in [1,3]] {grid} If you were going to **buy and use a DIY Grey squirrel pest control product** for you or your family to use on your property, how important would you consider each of the factors below in making your choice of product?
-[q9i_1] Solves problem quickly
-[q9i_2] Long lasting effect
-[q9i_3] Low cost
-[q9i_4] Non-lethal (does not involve killing)
-[q9i_5] Welfare-friendly for the target pest
-[q9i_6] Easy to use
-[q9i_7] Hygienic (requires me to have minimal contact with dead animal)
-[q9i_8] Safe for people and pets
-[q9i_9] Safe for non-target wild animals

<1> Very important
<2> Somewhat important
<3> Neutral
<4> Not very important
<5> Not at all important

[q11i if q8a_9 in [2,3]] {grid} If you were going to **employ a professional Grey squirrel pest control service** to treat a problem on your property, how important would you consider each of the factors below in making your choice of professional service?
-[q11i_1] Solves problem quickly
-[q11i_2] Long lasting effect
-[q11i_3] Low cost
-[q11i_4] Non-lethal (does not involve killing)
-[q11i_5] Welfare-friendly for the target pest
-[q11i_6] Easy to use
-[q11i_7] Hygienic (requires the user to have minimal contact with dead animal)
-[q11i_8] Safe for people and pets
-[q11i_9] Safe for non-target wild animals

<1> Very important
<2> Somewhat important
<3> Neutral
<4> Not very important
<5> Not at all important

[q9j if q8a_10 in [1,3]] {grid} If you were going to **buy and use DIY Wasp pest control product** for you or your family to use on your property, how important would you consider each of the factors below in making your choice of product?
-[q9j_1] Solves problem quickly
-[q9j_2] Long lasting effect
-[q9j_3] Low cost
-[q9j_4] Non-lethal (does not involve killing)
-[q9j_5] Welfare-friendly for the target pest
-[q9j_6] Easy to use
-[q9j_7] Hygienic (requires me to have minimal contact with dead animal)

-[q9j_8] Safe for people and pets
-[q9j_9] Safe for non-target wild animals

<1> Very important
<2> Somewhat important
<3> Neutral
<4> Not very important
<5> Not at all important

[q11j if q8a_10 in [2,3]] {grid} If you were going to **employ a professional Wasp pest control service** to treat a problem on your property, how important would you consider each of the factors below in making your choice of professional service?
-[q11j_1] Solves problem quickly
-[q11j_2] Long lasting effect
-[q11j_3] Low cost
-[q11j_4] Non-lethal (does not involve killing)
-[q11j_5] Welfare-friendly for the target pest
-[q11j_6] Easy to use
-[q11j_7] Hygienic (requires the user to have minimal contact with dead animal)
-[q11j_8] Safe for people and pets
-[q11j_9] Safe for non-target wild animals

<1> Very important
<2> Somewhat important
<3> Neutral
<4> Not very important
<5> Not at all important

#asked of everyone unless they've experienced a problem with all 10 species, it only lists those species they haven't had a problem with
[q8b if not q5.has_all([1,2,3,4,5,6,7,8,9,10])] {grid} In the future, if you experienced a problem with each of these species on your property, would you be most likely to consider **buying a DIY pest control product** or **employing a professional pest control service** to treat the problem on your property? Please tick one of the following for each species.
-[q8b_1 if not 1 in q5] Badger
-[q8b_2 if not 2 in q5] Fox
-[q8b_3 if not 3 in q5] Mole
-[q8b_4 if not 4 in q5] Mouse
-[q8b_5 if not 5 in q5] Pigeon
-[q8b_6 if not 6 in q5] Rabbit
-[q8b_7 if not 7 in q5] Rat
-[q8b_8 if not 8 in q5] Gull (including seagull)
-[q8b_9 if not 9 in q5] Grey squirrel
-[q8b_10 if not 10 in q5] Wasp

<1> Most likely to consider buying a DIY pest control product
<2> Most likely to consider employing a professional pest control service
<3> Equally likely to consider choosing either
<99> Would do neither

#all
[q7] {grid-check displaymax=5} Do you believe any of the following statements are true for any of the following species? Tick all the statements that you believe are true for each species, or tick 'none of these'.
-[q7_1] Badger
-[q7_2] Fox
-[q7_3] Mole
-[q7_4] Mouse
-[q7_5] Pigeon
-[q7_6] Rabbit
-[q7_7] Rat
-[q7_8] Gull (including seagull)
-[q7_9] Grey squirrel
-[q7_10] Wasp

<1> They eat our food
<2> They come into our homes
<3> They spread disease to people
<4> They attack people
<5> They spread disease to pets / domestic poultry etc.
<6> They attack pets / domestic poultry etc.
<7> They damage our property
<8> Their numbers are out of control
<9> I'm afraid of them
<99 xor> None of these

#all
[q13] {multiple} If you were experiencing a problem with wild animals on your property, where would you look to find out more information on dealing with the problem? Please tick all that apply, or tick "Do nothing".
<1> Your local council
<2> Internet search
<3> Government or public body website, e.g. DEFRA, Natural England
<4> Pest control company
<5> Wildlife protection group
<6> Hardware store
<7> Garden centre
<96> Other [q13_other] {open}
<99 xor> Do nothing

#all
[q12a] {grid-check transpose=true} Who do you think should ... Please tick all that apply, or tick "Not sure".
-[q12a_1] Control a wild animal infestation on a resident's property
-[q12a_2] Pay for the control of a wild animal infestation on a resident's property

<1> The resident
<2> The (non-resident) owner (e.g. landlord, local council or housing association)
<3> Your local council
<4> Whoever is at fault
<5> The animals should not be disturbed
<99 xor> Not sure

{page demo_intro}

And finally some questions about your home and household

{end page demo_intro}

[q14] {multiple} Please tick all those that apply to your main household unit, or tick “None of the above”. Do you…?
<1> Have any children (0-16 years) living with you
<2> Have a garden
<3> Keep any cats or dogs
<4> Keep any pets or domestic poultry in outdoor pens / aviaries in your garden (e.g. rabbits/guinea pigs/chickens/ducks/other birds)
<5> Use an outdoor store for animal feed
<6> Feed wild birds or animals in your garden
<7> Make your own compost
<8> Store food waste in an outdoor bin for collection by the council
<99 fixed xor> None of the above

[q15] How old is your home? Please tick the period in which you think your house was built.
<1> Pre-Victorian (before 1836)
<2> Victorian (1837-1901)
<3> 20th Century Pre World War II (1902-1945)
<4> 20th Century Post World War II (1946-2000)
<5> 21st century (since 2001)
<99> Don’t know

[ONS_urban_pdl if 0] {pdl-update ONS_urban}

[house_type_pdl if pdl.house_type.last > months(12)] {pdl-update house_type}
#{single varlabel = "House type lived in"} What sort of home do you currently live in?
#<1> Detached house
#<2> Semi-detached house
#<3> Terraced house
#<4> Maisonette
#<5> Studio/Flat
#<6> Bungalow
#<7> Static Caravan
#<95 fixed xor> Other
#<96 fixed xor> Don't know
#<99 fixed xor> Prefer not to say

[profile_house_tenure_pdl if pdl.profile_house_tenure.last > months(12)] {pdl-update profile_house_tenure}
#{single varlabel="House Tenure"} Do you own or rent the home in which you live?
#<1>Own – outright
#<2>Own – with a mortgage
#<3>Own (part-own) – through shared ownership scheme (i.e. pay part mortgage, part rent)
#<4>Rent – from a private landlord
#<5>Rent – from my local authority
#<6>Rent – from a housing association

#<7>Neither – I live with my parents, family or friends but pay some rent to them
#<8>Neither – I live rent-free with my parents, family or friends
#<9>Other

[profile_religion_pdl if pdl.profile_religion.last > months(12)] {pdl-update profile_religion}
#{single varlabel="Religious affiliation"} Do you regard yourself as belonging to any particular religion, and if so, to which of these do you belong?
#<1> No, I do not regard myself as belonging to any particular religion.
#<2> Yes - Church of England/Anglican/Episcopal
#<3> Yes - Roman Catholic
#<4> Yes - Presbyterian/Church of Scotland
#<5> Yes - Methodist
#<6> Yes - Baptist
#<17> Yes – Orthodox Christian
#<18> Yes - Pentecostal (e.g. Assemblies of God, Elim Pentecostal Church, New Testament Church of God, Redeemed Christian Church of God)
#<19> Yes - Evangelical – independent/non-denominational (e.g. FIEC, Pioneer, Vineyard, Newfrontiers)
#<7> Yes - United Reformed Church
#<8> Yes - Free Presbyterian
#<9> Yes - Brethren
#<10> Yes - Judaism
#<11> Yes - Hinduism
#<12> Yes - Islam
#<13> Yes - Sikhism
#<14> Yes - Buddhism
#<15> Yes - Other
#<16> Prefer not to say

[ethnicity_new_pdl if pdl.ethnicity_new.last > months(24)] {pdl-update ethnicity_new}
#{single varlabel = "Ethnicity"} To which of these groups do you consider you belong? Please select one option only. (We ask the question in this way so that it is consistent with Census definitions.)
#<1> English / Welsh / Scottish / Northern Irish / British
#<2> Irish
#<3> Gypsy or Irish Traveller
#<4> Any other White background
#<5> White and Black Caribbean
#<6> White and Black African
#<7> White and Asian
#<8> Any other Mixed / Multiple ethnic background
#<9> Indian
#<10> Pakistani
#<11> Bangladeshi
#<12> Chinese
#<13> Any other Asian background
#<14> African
#<15> Caribbean
#<16> Any other Black / African / Caribbean background
#<17> Arab
#<18 fixed> Any other ethnic group
#<19 fixed> Prefer not to say

{page end}

Thank you for completing this survey; your results have been stored. We hope the results will help people to reduce pest problems as well as helping pest control companies to provide better pest control products and services.

{end page end}

Document S2. Model summaries for models of attitudes towards species. Attitudes towards foxes and squirrels could not be modelled.

Badgers

```
> summary(att_badBEST)
formula: 
ATT_BAD ~ HOME_AGENUM2 + BEH_KIDS + BEH_PETS + BEH_FEEDBI + BEH_COMP + 
TRTBAD_DSP + TRTBAD_DSA + TRTBAD_DAM + TRTBAD_NUM + TRTBAD_FEA + BEL_BAD
data:    cdata

 link  threshold nobs logLik   AIC     niter max.grad cond.H 
 logit flexible  1828 -2175.47 4380.94 6(0)  1.06e-09 3.7e+02

Coefficients:
              Estimate Std. Error z value Pr(>|z|)    
HOME_AGENUM2  -0.15215    0.04522  -3.365 0.000765 ***
BEH_KIDSYes    0.23129    0.11182   2.069 0.038593 *  
BEH_PETSYes   -0.66383    0.09214  -7.205 5.81e-13 ***
BEH_FEEDBIYes -0.49600    0.09746  -5.089 3.60e-07 ***
BEH_COMPYes   -0.28877    0.10884  -2.653 0.007973 ** 
TRTBAD_DSPYes  0.57441    0.15753   3.646 0.000266 ***
TRTBAD_DSAYes  0.94355    0.11061   8.530  < 2e-16 ***
TRTBAD_DAMYes  0.51914    0.14087   3.685 0.000229 ***
TRTBAD_NUMYes  1.27104    0.22007   5.776 7.67e-09 ***
TRTBAD_FEAYes  1.09412    0.26074   4.196 2.71e-05 ***
BEL_BADYes     0.67150    0.13691   4.905 9.35e-07 ***
---
Signif. codes:  0 '***' 0.001 '**' 0.01 '*' 0.05 '.' 0.1 ' ' 1

Threshold coefficients:
          Estimate Std. Error z value
Vpos|Spos  -1.0294     0.1350  -7.625
Spos|Neut   0.3316     0.1329   2.495
Neut|Sneg   2.5603     0.1560  16.416
Sneg|Vneg   4.4912     0.2409  18.642
```

Moles

```
> summary(att_molBEST)
formula: 
ATT_MOL ~ DEM_AGE + HOME_TYPE2 + HOME_AGENUM2 + BEH_PETS + BEH_FEEDST + 
BEH_FEEDBI + TRTMOL_DSP + TRTMOL_DAM + TRTMOL_NUM + TRTMOL_FEA + EXP_MOL 
+ BEL_MOL
data:    cdata

 link  threshold nobs logLik   AIC     niter max.grad cond.H 
 logit flexible  1828 -2382.32 4804.63 6(0)  1.23e-11 5.1e+05

Coefficients:
                   Estimate Std. Error z value Pr(>|z|)    
DEM_AGE            0.007943   0.002830   2.806  0.00501 ** 
HOME_TYPE2Other   -0.594082   0.356644  -1.666  0.09576 .  
HOME_TYPE2Detach  -0.071039   0.185117  -0.384  0.70116    
HOME_TYPE2Flamai  -0.997845   0.194323  -5.135 2.82e-07 ***
HOME_TYPE2Semidet -0.440017   0.178117  -2.470  0.01350 *  
HOME_TYPE2Terrace -0.484048   0.188011  -2.575  0.01004 *  
HOME_AGENUM2      -0.142054   0.045654  -3.112  0.00186 ** 
BEH_PETSYes       -0.519923   0.091896  -5.658 1.53e-08 ***
BEH_FEEDSTYes      0.383973   0.193109   1.988  0.04677 *  
BEH_FEEDBIYes     -0.720359   0.098119  -7.342 2.11e-13 ***
TRTMOL_DSPYes      1.690446   0.339319   4.982 6.30e-07 ***
TRTMOL_DAMYes      0.726058   0.102772   7.065 1.61e-12 ***
TRTMOL_NUMYes      1.580962   0.312573   5.058 4.24e-07 ***
TRTMOL_FEAYes      1.570303   0.366516   4.284 1.83e-05 ***
EXP_MOLYes         0.821674   0.201291   4.082 4.46e-05 ***
BEL_MOLYes         0.867087   0.106019   8.179 2.87e-16 ***
---
Signif. codes:  0 '***' 0.001 '**' 0.01 '*' 0.05 '.' 0.1 ' ' 1
```

```
Threshold coefficients:
          Estimate Std. Error z value
Vpos|Spos  -1.3501     0.2479  -5.446
Spos|Neut  -0.1864     0.2453  -0.760
Neut|Sneg   1.6832     0.2505   6.718
Sneg|Vneg   3.8789     0.2856  13.581
```

Mice

```
> summary(att_mouBEST)
formula:
ATT_MOU ~ DEM_AGE + HOME_AGENUM2 + HOME_TENU2 + BEH_KIDS + BEH_PETS
+ BEH_FEEDST + BEH_FEEDBI + BEH_COMP + TRTMOU_DSP + TRTMOU_DSA +
TRTMOU_ATA + TRTMOU_NUM + TRTMOU_FEA + EXP_MOU + BEL_MOU
data:    cdata

 link  threshold nobs logLik   AIC     niter max.grad cond.H
 logit flexible  1828 -2543.64 5131.29 5(0)  2.01e-08 3.3e+05

Coefficients:
                    Estimate Std. Error z value Pr(>|z|)
DEM_AGE             0.018595   0.003265   5.695 1.23e-08 ***
HOME_AGENUM2       -0.188821   0.044619  -4.232 2.32e-05 ***
HOME_TENU2Other    -0.154900   0.359542  -0.431 0.666596
HOME_TENU2Own      -0.149127   0.169753  -0.878 0.379674
HOME_TENU2Social   -0.618903   0.198554  -3.117 0.001827 **
HOME_TENU2Rentpriv -0.654010   0.181680  -3.600 0.000318 ***
BEH_KIDSYes         0.285242   0.116565   2.447 0.014402 *
BEH_PETSYes        -0.510920   0.089899  -5.683 1.32e-08 ***
BEH_FEEDSTYes       0.415995   0.190546   2.183 0.029023 *
BEH_FEEDBIYes      -0.504027   0.097657  -5.161 2.45e-07 ***
BEH_COMPYes        -0.260961   0.106001  -2.462 0.013821 *
TRTMOU_DSPYes       0.627773   0.113980   5.508 3.63e-08 ***
TRTMOU_DSAYes       0.395685   0.132306   2.991 0.002784 **
TRTMOU_ATAYes       0.902514   0.354196   2.548 0.010832 *
TRTMOU_NUMYes       0.955743   0.137984   6.926 4.31e-12 ***
TRTMOU_FEAYes       1.204147   0.190446   6.323 2.57e-10 ***
EXP_MOUYes          0.526122   0.103598   5.078 3.80e-07 ***
BEL_MOUYes          0.855455   0.096955   8.823  < 2e-16 ***
---
Signif. codes:  0 ‘***’ 0.001 ‘**’ 0.01 ‘*’ 0.05 ‘.’ 0.1 ‘ ’ 1

Threshold coefficients:
          Estimate Std. Error z value
Vpos|Spos  -1.0239     0.2119  -4.832
Spos|Neut   0.2356     0.2090   1.127
Neut|Sneg   1.7219     0.2128   8.090
Sneg|Vneg   3.4730     0.2275  15.265
```

Pigeons

```
> summary(att_pigBEST)
formula:
ATT_PIG ~ HOME_AGENUM2 + HOME_TENU2 + BEH_PETS + BEH_FEEDBI + TRTPIG_DSP
+ TRTPIG_DSA + TRTPIG_NUM + TRTPIG_FEA + EXP_PIG + BEL_PIG
data:    cdata

 link  threshold nobs logLik   AIC     niter max.grad cond.H
 logit flexible  1828 -2481.49 4996.97 5(0)  3.72e-08 9.3e+02

Coefficients:
                     Estimate Std. Error z value Pr(>|z|)
HOME_AGENUM2       -1.445e-01  4.443e-02  -3.252  0.00115 **
HOME_TENU2Other    -9.142e-05  3.403e-01   0.000  0.99979
HOME_TENU2Own       2.353e-01  1.548e-01   1.520  0.12863
HOME_TENU2Social   -3.472e-01  1.910e-01  -1.818  0.06908 .
HOME_TENU2Rentpriv -2.651e-01  1.803e-01  -1.470  0.14161
BEH_PETSYes        -4.087e-01  8.872e-02  -4.606 4.10e-06 ***
BEH_FEEDBIYes      -4.798e-01  9.143e-02  -5.248 1.54e-07 ***
TRTPIG_DSPYes       5.173e-01  1.108e-01   4.668 3.05e-06 ***
TRTPIG_DSAYes       5.343e-01  1.241e-01   4.304 1.68e-05 ***
TRTPIG_NUMYes       1.340e+00  1.029e-01  13.013  < 2e-16 ***
```

```
TRTPIG_FEAYes       1.386e+00  3.088e-01   4.487 7.23e-06 ***
EXP_PIGYes          9.956e-01  1.658e-01   6.005 1.92e-09 ***
BEL_PIGYes          8.313e-01  1.025e-01   8.109 5.11e-16 ***
---
Signif. codes:  0 ‘***’ 0.001 ‘**’ 0.01 ‘*’ 0.05 ‘.’ 0.1 ‘ ’ 1

Threshold coefficients:
          Estimate Std. Error z value
Vpos|Spos  -2.0481     0.1934 -10.592
Spos|Neut  -0.8242     0.1853  -4.448
Neut|Sneg   0.8025     0.1857   4.321
Sneg|Vneg   2.6661     0.1988  13.414
```

Rabbits

```
> summary(att_rabBEST)
formula:
ATT_RAB ~ DEM_GENDER + DEM_AGE + HOME_TENU2 + BEH_PETS + BEH_FEEDBI +
TRTRAB_DSP + TRTRAB_ATP + TRTRAB_DAM + TRTRAB_NUM + TRTRAB_FEA + EXP_RAB
+ BEL_RAB
data:    cdata

 link  threshold nobs logLik   AIC     niter max.grad cond.H
 logit flexible  1828 -2175.97 4389.94 6(0)  3.64e-08 5.7e+05

Coefficients:
                    Estimate Std. Error z value Pr(>|z|)
DEM_GENDERMale      0.187833   0.088488   2.123  0.03378 *
DEM_AGE             0.022520   0.003183   7.076 1.49e-12 ***
HOME_TENU2Other    -0.257583   0.364812  -0.706  0.48014
HOME_TENU2Own      -0.050723   0.173920  -0.292  0.77056
HOME_TENU2Social   -0.329359   0.204470  -1.611  0.10722
HOME_TENU2Rentpriv -0.612250   0.187606  -3.263  0.00110 **
BEH_PETSYes        -0.472971   0.090903  -5.203 1.96e-07 ***
BEH_FEEDBIYes      -0.542759   0.096345  -5.633 1.77e-08 ***
TRTRAB_DSPYes       1.099654   0.281258   3.910 9.24e-05 ***
TRTRAB_ATPYes       1.196869   0.522088   2.292  0.02188 *
TRTRAB_DAMYes       0.762756   0.131010   5.822 5.81e-09 ***
TRTRAB_NUMYes       1.108237   0.158064   7.011 2.36e-12 ***
TRTRAB_FEAYes       1.802641   0.622879   2.894  0.00380 **
EXP_RABYes          1.012872   0.313644   3.229  0.00124 **
BEL_RABYes          0.873941   0.153882   5.679 1.35e-08 ***
---
Signif. codes:  0 ‘***’ 0.001 ‘**’ 0.01 ‘*’ 0.05 ‘.’ 0.1 ‘ ’ 1

Threshold coefficients:
          Estimate Std. Error z value
Vpos|Spos  0.05435    0.18785   0.289
Spos|Neut  1.75498    0.19205   9.138
Neut|Sneg  4.02469    0.21829  18.437
Sneg|Vneg  5.84826    0.28505  20.517
```

Rats

```
> summary(att_ratTOP)
formula:
ATT_RAT ~ DEM_AGE + HOME_AGENUM2 + HOME_TENU2 + BEH_PETS + TRTRAT_DSP
+ TRTRAT_ATP + TRTRAT_DSA + TRTRAT_NUM + TRTRAT_FEA + EXP_RAT + BEL_RAT
data:    cdata

 link  threshold nobs logLik   AIC     niter max.grad cond.H
 logit flexible  1828 -2148.67 4333.34 5(0)  6.23e-10 3.2e+05

Coefficients:
                    Estimate Std. Error z value Pr(>|z|)
DEM_AGE             0.018114   0.003191   5.677 1.37e-08 ***
HOME_AGENUM2       -0.184205   0.047611  -3.869 0.000109 ***
HOME_TENU2Other     0.193409   0.383198   0.505 0.613753
HOME_TENU2Own       0.141873   0.174560   0.813 0.416362
HOME_TENU2Social   -0.267904   0.207357  -1.292 0.196359
HOME_TENU2Rentpriv -0.316423   0.186361  -1.698 0.089525 .
```

```
BEH_PETSYes        -0.561503   0.093657  -5.995 2.03e-09 ***
TRTRAT_DSPYes       0.625560   0.101163   6.184 6.26e-10 ***
TRTRAT_ATPYes       0.565196   0.175414   3.222 0.001273 **
TRTRAT_DSAYes       0.392264   0.102532   3.826 0.000130 ***
TRTRAT_NUMYes       0.843750   0.105887   7.968 1.61e-15 ***
TRTRAT_FEAYes       1.205890   0.143364   8.411  < 2e-16 ***
EXP_RATYes          0.538773   0.121553   4.432 9.32e-06 ***
BEL_RATYes          1.067464   0.123245   8.661  < 2e-16 ***
---
Signif. codes:  0 ‘***’ 0.001 ‘**’ 0.01 ‘*’ 0.05 ‘.’ 0.1 ‘ ’ 1

Threshold coefficients:
          Estimate Std. Error z value
Vpos|Spos  -1.1469     0.2402  -4.774
Spos|Neut   0.0822     0.2290   0.359
Neut|Sneg   1.3153     0.2297   5.726
Sneg|Vneg   2.7457     0.2373  11.571
```

Gulls

```
> summary(att_gulBEST)
formula:
ATT_GUL ~ HOME_AGENUM2 + HOME_TENU2 + BEH_PETS + BEH_FEEDBI + TRTGUL_DSP
+ TRTGUL_ATP + TRTGUL_DSA + TRTGUL_ATA + TRTGUL_NUM + TRTGUL_FEA +
EXP_GUL + BEL_GUL
data:    cdata

 link  threshold nobs logLik   AIC     niter max.grad cond.H
 logit flexible  1828 -2431.77 4901.55 5(0)  9.99e-08 9.4e+02

Coefficients:
                   Estimate Std. Error z value Pr(>|z|)
HOME_AGENUM2       -0.15241    0.04452  -3.423 0.000619 ***
HOME_TENU2Other    -0.15564    0.34154  -0.456 0.648595
HOME_TENU2Own      -0.21220    0.15654  -1.356 0.175234
HOME_TENU2Social   -0.70799    0.19269  -3.674 0.000239 ***
HOME_TENU2Rentpriv -0.67423    0.18271  -3.690 0.000224 ***
BEH_PETSYes        -0.28462    0.08940  -3.184 0.001453 **
BEH_FEEDBIYes      -0.32531    0.09135  -3.561 0.000369 ***
TRTGUL_DSPYes       0.51196    0.17565   2.915 0.003562 **
TRTGUL_ATPYes       0.43710    0.10253   4.263 2.02e-05 ***
TRTGUL_DSAYes       0.40862    0.18806   2.173 0.029794 *
TRTGUL_ATAYes       0.37497    0.16824   2.229 0.025829 *
TRTGUL_NUMYes       1.19672    0.10722  11.162  < 2e-16 ***
TRTGUL_FEAYes       0.82162    0.21630   3.799 0.000146 ***
EXP_GULYes          0.91774    0.20532   4.470 7.83e-06 ***
BEL_GULYes          0.92461    0.10015   9.233  < 2e-16 ***
---
Signif. codes:  0 ‘***’ 0.001 ‘**’ 0.01 ‘*’ 0.05 ‘.’ 0.1 ‘ ’ 1

Threshold coefficients:
          Estimate Std. Error z value
Vpos|Spos -2.84372    0.20375 -13.957
Spos|Neut -1.61090    0.19074  -8.445
Neut|Sneg  0.05515    0.18576   0.297
Sneg|Vneg  1.99736    0.19497  10.244
```

Wasps

```
> summary(att_wasBEST)
formula:
ATT_WAS ~ DEM_GENDER + DEM_SOCGRADE2 + HOME_AGENUM2 + BEH_FEEDBI + TRTWAS_DSP + T
RTWAS_ATP + TRTWAS_ATA + TRTWAS_DAM + TRTWAS_NUM + TRTWAS_FEA + EXP_WAS + BEL_WAS
data:    cdata

 link  threshold nobs logLik   AIC     niter max.grad cond.H
 logit flexible  1828 -2332.28 4696.57 5(0)  2.49e-08 1.1e+03

Coefficients:
```

```
               Estimate Std. Error z value Pr(>|z|)
DEM_GENDERMale -0.18005    0.09057  -1.988  0.04681 *
DEM_SOCGRADE2  -0.08826    0.03904  -2.261  0.02376 *
HOME_AGENUM2   -0.13374    0.04500  -2.972  0.00296 **
BEH_FEEDBIYes  -0.28813    0.09068  -3.178  0.00148 **
TRTWAS_DSPYes   0.84456    0.39191   2.155  0.03116 *
TRTWAS_ATPYes   0.64512    0.09597   6.722 1.80e-11 ***
TRTWAS_ATAYes   0.41267    0.21710   1.901  0.05732 .
TRTWAS_DAMYes   0.27987    0.13658   2.049  0.04045 *
TRTWAS_NUMYes   1.37836    0.17805   7.741 9.84e-15 ***
TRTWAS_FEAYes   1.07495    0.11445   9.392  < 2e-16 ***
EXP_WASYes      0.22767    0.10370   2.195  0.02813 *
BEL_WASYes      1.02201    0.10236   9.984  < 2e-16 ***
---
Signif. codes:  0 ‘***’ 0.001 ‘**’ 0.01 ‘*’ 0.05 ‘.’ 0.1 ‘ ’ 1

Threshold coefficients:
          Estimate Std. Error z value
Vpos|Spos  -2.5986     0.1997 -13.010
Spos|Neut  -1.4119     0.1833  -7.702
Neut|Sneg   0.1524     0.1786   0.853
Sneg|Vneg   1.7182     0.1837   9.354
```

Document S3. Model summaries for models of experience of problems with species personally. Experience of problems with squirrels and wasps could not be modelled.

```
Badgers
> summary(exp_badgerBEST)

Call:
glm(formula = EXP_BAD ~ DEM_AGE + HOME_AGENUM2 + BEH_FEEDST +
    TRTBAD_DSP + TRTBAD_DAM + BEL_BAD, family = binomial, data = cdata,
    na.action = na.fail)

Deviance Residuals:
    Min       1Q   Median       3Q      Max
-1.4853  -0.1197  -0.0795  -0.0517   3.7785

Coefficients:
              Estimate Std. Error z value Pr(>|z|)
(Intercept)   -9.00190    1.03817  -8.671  < 2e-16 ***
DEM_AGE        0.04005    0.01257   3.187  0.00144 **
HOME_AGENUM2   0.41141    0.17856   2.304  0.02122 *
BEH_FEEDSTYes  1.24179    0.50329   2.467  0.01361 *
TRTBAD_DSPYes -2.07077    1.04349  -1.984  0.04720 *
TRTBAD_DAMYes  2.31790    0.40816   5.679 1.36e-08 ***
BEL_BADYes     2.23536    0.41694   5.361 8.26e-08 ***
---
Signif. codes:  0 ‘***’ 0.001 ‘**’ 0.01 ‘*’ 0.05 ‘.’ 0.1 ‘ ’ 1

(Dispersion parameter for binomial family taken to be 1)

    Null deviance: 384.89  on 1827  degrees of freedom
Residual deviance: 238.01  on 1821  degrees of freedom
AIC: 252.01

Number of Fisher Scoring iterations: 8
```

```
Foxes
> summary(exp_foxBEST)

Call:
glm(formula = EXP_FOX ~ DEM_AGE + DEM_REGCAT + HOME_AGENUM2 +
    BEH_FEEDST + BEH_COMP + TRTFOX_DAM + TRTFOX_NUM + BEL_FOX +
    ATT_FOX2, family = binomial, data = cdata, na.action = na.fail)

Deviance Residuals:
    Min       1Q   Median       3Q      Max
-1.8050  -0.3555  -0.1911  -0.1260   3.1893

Coefficients:
                    Estimate Std. Error z value Pr(>|z|)
(Intercept)        -6.321830   0.496131 -12.742  < 2e-16 ***
DEM_AGE             0.016637   0.006016   2.766 0.005680 **
DEM_REGCATMEngland -0.690415   0.289457  -2.385 0.017070 *
DEM_REGCATNEngland -0.968478   0.293288  -3.302 0.000960 ***
DEM_REGCATNIreland  0.475874   0.869466   0.547 0.584161
DEM_REGCATScotland -0.555374   0.372421  -1.491 0.135895
DEM_REGCATWales     0.003315   0.423010   0.008 0.993747
HOME_AGENUM2        0.260500   0.094895   2.745 0.006048 **
BEH_FEEDSTYes       0.927849   0.315152   2.944 0.003239 **
BEH_COMPYes         0.715862   0.210899   3.394 0.000688 ***
TRTFOX_DAMYes       0.690200   0.217903   3.167 0.001538 **
TRTFOX_NUMYes       0.489329   0.240141   2.038 0.041582 *
BEL_FOXYes          1.982978   0.240031   8.261  < 2e-16 ***
ATT_FOX2            0.419084   0.086647   4.837 1.32e-06 ***
---
Signif. codes:  0 ‘***’ 0.001 ‘**’ 0.01 ‘*’ 0.05 ‘.’ 0.1 ‘ ’ 1

(Dispersion parameter for binomial family taken to be 1)
```

```
    Null deviance: 1131.24  on 1827  degrees of freedom
Residual deviance:  778.86  on 1814  degrees of freedom
AIC: 806.86

Number of Fisher Scoring iterations: 6
```

Moles

```
> summary(exp_molBEST)

Call:
glm(formula = EXP_MOL ~ DEM_ONSURBAN + BEH_FEEDST + BEH_COMP + 
    TRTMOL_DAM + BEL_MOL + ATT_MOL2, family = binomial, data = cdata, 
    na.action = na.fail)

Deviance Residuals: 
    Min       1Q   Median       3Q      Max  
-1.7567  -0.3241  -0.1796  -0.1179   3.2896  

Coefficients:
                     Estimate Std. Error z value Pr(>|z|)    
(Intercept)           -4.1132     0.4044 -10.171  < 2e-16 ***
DEM_ONSURBANTownfrin  -0.8856     0.3504  -2.528  0.01148 *  
DEM_ONSURBANUrban     -1.7330     0.2529  -6.852 7.26e-12 ***
BEH_FEEDSTYes          1.0021     0.3122   3.209  0.00133 ** 
BEH_COMPYes            0.6072     0.2261   2.685  0.00725 ** 
TRTMOL_DAMYes          0.5643     0.2425   2.327  0.01997 *  
BEL_MOLYes             1.4818     0.2718   5.451 5.00e-08 ***
ATT_MOL2               0.4401     0.1029   4.278 1.89e-05 ***
---
Signif. codes:  0 ‘***’ 0.001 ‘**’ 0.01 ‘*’ 0.05 ‘.’ 0.1 ‘ ’ 1

(Dispersion parameter for binomial family taken to be 1)

    Null deviance: 858.80  on 1827  degrees of freedom
Residual deviance: 629.94  on 1820  degrees of freedom
AIC: 645.94

Number of Fisher Scoring iterations: 7
```

Mice

```
> summary(exp_mouBEST)

Call:
glm(formula = EXP_MOU ~ DEM_AGE + DEM_ONSURBAN + DEM_REGCAT + 
    HOME_AGENUM2 + BEH_FEEDST + TRTMOU_HOM + TRTMOU_DSP + TRTMOU_DAM + 
    BEL_MOU + ATT_MOU2, family = binomial, data = cdata, na.action = na.fail)

Deviance Residuals: 
    Min       1Q   Median       3Q      Max  
-1.9374  -0.8261  -0.4070   0.8968   2.5739  

Coefficients:
                      Estimate Std. Error z value Pr(>|z|)    
(Intercept)          -3.728484   0.364488 -10.229  < 2e-16 ***
DEM_AGE               0.007793   0.003544   2.199 0.027887 *  
DEM_ONSURBANTownfrin -0.482453   0.253391  -1.904 0.056912 .  
DEM_ONSURBANUrban    -0.570019   0.194341  -2.933 0.003356 ** 
DEM_REGCATMEngland   -0.400950   0.170193  -2.356 0.018480 *  
DEM_REGCATNEngland   -0.415897   0.148391  -2.803 0.005068 ** 
DEM_REGCATNIreland    0.628880   0.472036   1.332 0.182771    
DEM_REGCATScotland   -0.007049   0.206592  -0.034 0.972782    
DEM_REGCATWales      -0.125991   0.280590  -0.449 0.653416    
HOME_AGENUM2          0.276753   0.059695   4.636 3.55e-06 ***
BEH_FEEDSTYes         0.600216   0.241912   2.481 0.013097 *  
TRTMOU_HOMYes         0.363136   0.128651   2.823 0.004763 ** 
TRTMOU_DSPYes         0.484829   0.131857   3.677 0.000236 ***
TRTMOU_DAMYes         0.516488   0.122093   4.230 2.33e-05 ***
```

```
BEL_MOUYes            1.516937   0.149789  10.127  < 2e-16 ***
ATT_MOU2              0.273653   0.050716   5.396 6.82e-08 ***
---
Signif. codes:  0 ‘***’ 0.001 ‘**’ 0.01 ‘*’ 0.05 ‘.’ 0.1 ‘ ’ 1

(Dispersion parameter for binomial family taken to be 1)

    Null deviance: 2265.5  on 1827  degrees of freedom
Residual deviance: 1813.3  on 1812  degrees of freedom
AIC: 1845.3

Number of Fisher Scoring iterations: 5
```

Pigeons

```
> summary(exp_pigBEST)

Call:
glm(formula = EXP_PIG ~ DEM_GENDER + DEM_AGE + BEH_COMP + TRTPIG_DAM +
    BEL_PIG + ATT_PIG2, family = binomial, data = cdata, na.action = na.fail)

Deviance Residuals:
    Min       1Q   Median       3Q      Max
-1.5606  -0.3841  -0.1892  -0.1247   3.3534

Coefficients:
                Estimate Std. Error z value Pr(>|z|)
(Intercept)    -7.203385   0.514412 -14.003  < 2e-16 ***
DEM_GENDERMale  0.446312   0.182125   2.451 0.014262 *
DEM_AGE         0.022097   0.005872   3.763 0.000168 ***
BEH_COMPYes     0.449757   0.195855   2.296 0.021654 *
TRTPIG_DAMYes   1.129994   0.183549   6.156 7.44e-10 ***
BEL_PIGYes      1.862090   0.245208   7.594 3.10e-14 ***
ATT_PIG2        0.504969   0.092936   5.434 5.53e-08 ***
---
Signif. codes:  0 ‘***’ 0.001 ‘**’ 0.01 ‘*’ 0.05 ‘.’ 0.1 ‘ ’ 1

(Dispersion parameter for binomial family taken to be 1)

    Null deviance: 1158.34  on 1827  degrees of freedom
Residual deviance:  845.06  on 1821  degrees of freedom
AIC: 859.06

Number of Fisher Scoring iterations: 6
```

Rabbits

```
> summary(exp_rabBEST)

Call:
glm(formula = EXP_RAB ~ DEM_GENDER + DEM_ONSURBAN + HOME_TYPE2 +
    HOME_AGENUM2 + BEL_RAB + ATT_RAB2, family = binomial, data = cdata,
    na.action = na.fail)

Deviance Residuals:
    Min       1Q   Median       3Q      Max
-1.9281  -0.1286  -0.0689  -0.0447   3.4645

Coefficients:
                     Estimate Std. Error z value Pr(>|z|)
(Intercept)           -6.4939     0.9037  -7.186 6.68e-13 ***
DEM_GENDERMale         0.8093     0.3984   2.031 0.042241 *
DEM_ONSURBANTownfrin  -0.1379     0.5677  -0.243 0.808085
DEM_ONSURBANUrban     -1.3116     0.4318  -3.037 0.002386 **
HOME_TYPE2Other        0.9887     0.8109   1.219 0.222697
HOME_TYPE2Detach      -0.6179     0.5345  -1.156 0.247594
HOME_TYPE2Flamai      -0.8668     0.6717  -1.290 0.196900
HOME_TYPE2Semidet     -1.9816     0.6585  -3.009 0.002620 **
HOME_TYPE2Terrace     -2.6447     0.8867  -2.983 0.002857 **
HOME_AGENUM2           0.5769     0.1657   3.481 0.000500 ***
```

```
BEL_RABYes              2.6739     0.3940   6.786 1.16e-11 ***
ATT_RAB2                0.6084     0.1713   3.550 0.000385 ***
---
Signif. codes:  0 ‘***’ 0.001 ‘**’ 0.01 ‘*’ 0.05 ‘.’ 0.1 ‘ ’ 1

(Dispersion parameter for binomial family taken to be 1)

    Null deviance: 429.61  on 1827  degrees of freedom
Residual deviance: 239.77  on 1816  degrees of freedom
AIC: 263.77

Number of Fisher Scoring iterations: 8
```

Rats

```
> summary(exp_ratTOP)

Call:
glm(formula = EXP_RAT ~ DEM_AGE + DEM_ONSURBAN + DEM_REGCAT +
    HOME_AGENUM2 + BEH_POUL + BEH_FEEDST + BEH_COMP + TRTRAT_DAM +
    BEL_RAT + ATT_RAT2, family = binomial, data = cdata, na.action = na.fail)

Deviance Residuals:
    Min       1Q   Median       3Q      Max
-1.7443  -0.7318  -0.4924  -0.2039   2.6755

Coefficients:
                      Estimate Std. Error z value Pr(>|z|)
(Intercept)          -4.666558   0.456596 -10.220  < 2e-16 ***
DEM_AGE               0.008547   0.003927   2.177  0.02951 *
DEM_ONSURBANTownfrin -0.734398   0.264409  -2.778  0.00548 **
DEM_ONSURBANUrban    -0.505116   0.194209  -2.601  0.00930 **
DEM_REGCATMEngland    0.060167   0.168850   0.356  0.72159
DEM_REGCATNEngland   -0.397334   0.158161  -2.512  0.01200 *
DEM_REGCATNIreland   -0.808453   0.698671  -1.157  0.24722
DEM_REGCATScotland   -0.769000   0.254798  -3.018  0.00254 **
DEM_REGCATWales       0.060199   0.283811   0.212  0.83202
HOME_AGENUM2          0.278742   0.061955   4.499 6.82e-06 ***
BEH_POULYes           0.730074   0.271377   2.690  0.00714 **
BEH_FEEDSTYes         0.531311   0.250259   2.123  0.03375 *
BEH_COMPYes           0.338864   0.138475   2.447  0.01440 *
TRTRAT_DAMYes         0.871403   0.127714   6.823 8.91e-12 ***
BEL_RATYes            1.256174   0.265688   4.728 2.27e-06 ***
ATT_RAT2              0.277558   0.065305   4.250 2.14e-05 ***
---
Signif. codes:  0 ‘***’ 0.001 ‘**’ 0.01 ‘*’ 0.05 ‘.’ 0.1 ‘ ’ 1

(Dispersion parameter for binomial family taken to be 1)

    Null deviance: 1948.5  on 1827  degrees of freedom
Residual deviance: 1668.9  on 1812  degrees of freedom
AIC: 1700.9

Number of Fisher Scoring iterations: 5
```

Gulls

```
> summary(exp_gulBEST)

Call:
glm(formula = EXP_GUL ~ DEM_GENDER + DEM_REGCAT + BEH_GARD +
    TRTGUL_DAM + BEL_GUL + ATT_GUL2, family = binomial, data = cdata,
    na.action = na.fail)

Deviance Residuals:
     Min        1Q    Median        3Q       Max
-1.38953  -0.33044  -0.13283  -0.08356   3.09504

Coefficients:
                   Estimate Std. Error z value Pr(>|z|)
```

```
(Intercept)         -6.8203     0.6370 -10.708  < 2e-16 ***
DEM_GENDERMale       0.4986     0.2221   2.245  0.02476 *
DEM_REGCATMEngland  -0.8746     0.4072  -2.148  0.03174 *
DEM_REGCATNEngland  -0.4199     0.2985  -1.407  0.15949
DEM_REGCATNireland   0.8452     0.8456   1.000  0.31755
DEM_REGCATScotland   0.3759     0.2965   1.268  0.20484
DEM_REGCATWales      0.4629     0.4255   1.088  0.27673
BEH_GARDYes         -0.6361     0.2401  -2.649  0.00807 **
TRTGUL_DAMYes        0.7240     0.2252   3.214  0.00131 **
BEL_GULYes           2.3639     0.3507   6.741 1.57e-11 ***
ATT_GUL2             0.6688     0.1328   5.035 4.78e-07 ***
---
Signif. codes:  0 ‘***’ 0.001 ‘**’ 0.01 ‘*’ 0.05 ‘.’ 0.1 ‘ ’ 1

(Dispersion parameter for binomial family taken to be 1)

    Null deviance: 847.96  on 1827  degrees of freedom
Residual deviance: 616.12  on 1817  degrees of freedom
AIC: 638.12

Number of Fisher Scoring iterations: 7
```

Document S4. Model summaries for models of belief that species causes problems for people in the UK generally.

Badgers

```
> summary(bel_badgerTOP)

Call:
glm(formula = BEL_BAD ~ DEM_REGCAT + TRTBAD_DSA + TRTBAD_DAM + 
    TRTBAD_NUM + EXP_BAD + ATT_BAD2, family = binomial, data = cdata, 
    na.action = na.fail)

Deviance Residuals: 
    Min       1Q   Median       3Q      Max  
-2.1386  -0.5044  -0.3553  -0.2946   2.7168  

Coefficients:
                     Estimate Std. Error z value Pr(>|z|)    
(Intercept)          -3.15239    0.20609 -15.297  < 2e-16 ***
DEM_REGCATMEngland   -0.08328    0.20589  -0.404   0.6859    
DEM_REGCATNEngland   -0.34845    0.19432  -1.793   0.0730 .  
DEM_REGCATNIreland    0.22450    0.54659   0.411   0.6813    
DEM_REGCATScotland   -0.73316    0.31314  -2.341   0.0192 *  
DEM_REGCATWales      -0.89759    0.41575  -2.159   0.0309 *  
TRTBAD_DSAYes         0.81674    0.15812   5.165 2.40e-07 ***
TRTBAD_DAMYes         1.26689    0.17619   7.190 6.46e-13 ***
TRTBAD_NUMYes         1.32379    0.24881   5.320 1.04e-07 ***
EXP_BADYes            2.24204    0.42676   5.254 1.49e-07 ***
ATT_BAD2              0.38491    0.07562   5.090 3.58e-07 ***
---
Signif. codes:  0 ‘***’ 0.001 ‘**’ 0.01 ‘*’ 0.05 ‘.’ 0.1 ‘ ’ 1

(Dispersion parameter for binomial family taken to be 1)

    Null deviance: 1544.7  on 1827  degrees of freedom
Residual deviance: 1234.3  on 1817  degrees of freedom
AIC: 1256.3

Number of Fisher Scoring iterations: 5
```

Foxes

```
> summary(bel_foxBEST)

Call:
glm(formula = BEL_FOX ~ DEM_ONSURBAN + BEH_FOODWA + TRTFOX_EAT + 
    TRTFOX_HOM + TRTFOX_DSA + TRTFOX_ATA + TRTFOX_DAM + TRTFOX_NUM + 
    EXP_FOX + ATT_FOX2, family = binomial, data = cdata, na.action = na.fail)

Deviance Residuals: 
    Min       1Q   Median       3Q      Max  
-2.9028  -0.7036  -0.4985   0.5890   2.2523  

Coefficients:
                        Estimate Std. Error z value Pr(>|z|)    
(Intercept)             -3.25792    0.26430 -12.326  < 2e-16 ***
DEM_ONSURBANTownfrin     0.33087    0.27977   1.183 0.236946    
DEM_ONSURBANUrban        0.48508    0.21760   2.229 0.025803 *  
BEH_FOODWAYes            0.24905    0.12366   2.014 0.044009 *  
TRTFOX_EATYes            0.47488    0.14156   3.355 0.000795 ***
TRTFOX_HOMYes            0.82231    0.18450   4.457 8.32e-06 ***
TRTFOX_DSAYes            0.28134    0.17328   1.624 0.104444    
TRTFOX_ATAYes            0.30293    0.12309   2.461 0.013850 *  
TRTFOX_DAMYes            1.30490    0.18615   7.010 2.38e-12 ***
TRTFOX_NUMYes            0.58094    0.21573   2.693 0.007083 ** 
EXP_FOXYes               1.95258    0.23946   8.154 3.52e-16 ***
ATT_FOX2                 0.50099    0.05783   8.662  < 2e-16 ***
```

```
---
Signif. codes:  0 ‘***’ 0.001 ‘**’ 0.01 ‘*’ 0.05 ‘.’ 0.1 ‘ ’ 1

(Dispersion parameter for binomial family taken to be 1)

    Null deviance: 2309.6  on 1827  degrees of freedom
Residual deviance: 1741.0  on 1816  degrees of freedom
AIC: 1765

Number of Fisher Scoring iterations: 5
```

Moles

```
> summary(bel_molTOP)

Call:
glm(formula = BEL_MOL ~ DEM_AGE + TRTMOL_DAM + EXP_MOL + ATT_MOL2, 
    family = binomial, data = cdata, na.action = na.fail)

Deviance Residuals: 
    Min       1Q   Median       3Q      Max  
-2.5045  -0.6901  -0.4974   0.8135   2.2584  

Coefficients:
               Estimate Std. Error z value Pr(>|z|)    
(Intercept)   -3.107658   0.227754 -13.645  < 2e-16 ***
DEM_AGE        0.009397   0.003456   2.719  0.00655 ** 
TRTMOL_DAMYes  1.706914   0.117203  14.564  < 2e-16 ***
EXP_MOLYes     1.580664   0.265155   5.961  2.5e-09 ***
ATT_MOL2       0.460233   0.054444   8.453  < 2e-16 ***
---
Signif. codes:  0 ‘***’ 0.001 ‘**’ 0.01 ‘*’ 0.05 ‘.’ 0.1 ‘ ’ 1

(Dispersion parameter for binomial family taken to be 1)

    Null deviance: 2330.8  on 1827  degrees of freedom
Residual deviance: 1826.1  on 1823  degrees of freedom
AIC: 1836.1

Number of Fisher Scoring iterations: 4
```

Mice

```
> summary(bel_mouBEST)

Call:
glm(formula = BEL_MOU ~ BEH_FOODWA + TRTMOU_EAT + TRTMOU_HOM + 
    TRTMOU_DAM + EXP_MOU + ATT_MOU2, family = binomial, data = cdata, 
    na.action = na.fail)

Deviance Residuals: 
    Min       1Q   Median       3Q      Max  
-2.6631  -0.9004   0.3687   0.8390   1.8694  

Coefficients:
              Estimate Std. Error z value Pr(>|z|)    
(Intercept)   -1.98700    0.15856 -12.532  < 2e-16 ***
BEH_FOODWAYes  0.24564    0.11833   2.076 0.037901 *  
TRTMOU_EATYes  0.44563    0.13536   3.292 0.000994 ***
TRTMOU_HOMYes  0.54216    0.12820   4.229 2.35e-05 ***
TRTMOU_DAMYes  0.87983    0.12809   6.869 6.47e-12 ***
EXP_MOUYes     1.48015    0.14594  10.142  < 2e-16 ***
ATT_MOU2       0.43118    0.04817   8.951  < 2e-16 ***
---
Signif. codes:  0 ‘***’ 0.001 ‘**’ 0.01 ‘*’ 0.05 ‘.’ 0.1 ‘ ’ 1

(Dispersion parameter for binomial family taken to be 1)

    Null deviance: 2460.4  on 1827  degrees of freedom
```

```
Residual deviance: 1906.0  on 1821  degrees of freedom
AIC: 1920

Number of Fisher Scoring iterations: 5
```

Pigeons

```
> summary(bel_pigBEST)

Call:
glm(formula = BEL_PIG ~ DEM_AGE + HOME_AGENUM2 + TRTPIG_DSP +
    TRTPIG_DAM + TRTPIG_NUM + EXP_PIG + ATT_PIG2, family = binomial,
    data = cdata, na.action = na.fail)

Deviance Residuals:
    Min       1Q   Median       3Q      Max
-2.6952  -0.7644  -0.5395   0.8552   2.2775

Coefficients:
               Estimate Std. Error z value Pr(>|z|)
(Intercept)   -3.516175   0.290061 -12.122  < 2e-16 ***
DEM_AGE        0.009567   0.003413   2.803  0.00506 **
HOME_AGENUM2   0.166398   0.058068   2.866  0.00416 **
TRTPIG_DSPYes  0.773899   0.128575   6.019 1.75e-09 ***
TRTPIG_DAMYes  1.076181   0.143854   7.481 7.37e-14 ***
TRTPIG_NUMYes  0.603964   0.125217   4.823 1.41e-06 ***
EXP_PIGYes     1.822655   0.247872   7.353 1.94e-13 ***
ATT_PIG2       0.409366   0.054369   7.529 5.10e-14 ***
---
Signif. codes:  0 ‘***’ 0.001 ‘**’ 0.01 ‘*’ 0.05 ‘.’ 0.1 ‘ ’ 1

(Dispersion parameter for binomial family taken to be 1)

    Null deviance: 2427.2  on 1827  degrees of freedom
Residual deviance: 1897.0  on 1820  degrees of freedom
AIC: 1913

Number of Fisher Scoring iterations: 4
```

Rabbits

```
> summary(bel_rabBEST)

Call:
glm(formula = BEL_RAB ~ HOME_AGENUM2 + BEH_FOODWA + TRTRAB_EAT +
    TRTRAB_ATP + TRTRAB_DAM + EXP_RAB + ATT_RAB2, family = binomial,
    data = cdata, na.action = na.fail)

Deviance Residuals:
    Min       1Q   Median       3Q      Max
-2.4427  -0.4252  -0.3265  -0.2604   2.7281

Coefficients:
              Estimate Std. Error z value Pr(>|z|)
(Intercept)   -4.36594    0.31841 -13.712  < 2e-16 ***
HOME_AGENUM2   0.16455    0.08035   2.048 0.040552 *
BEH_FOODWAYes  0.28697    0.16849   1.703 0.088525 .
TRTRAB_EATYes  0.71683    0.20544   3.489 0.000484 ***
TRTRAB_ATPYes  1.62108    0.70512   2.299 0.021505 *
TRTRAB_DAMYes  1.41526    0.17724   7.985 1.41e-15 ***
EXP_RABYes     2.24889    0.38556   5.833 5.45e-09 ***
ATT_RAB2       0.50471    0.08400   6.009 1.87e-09 ***
---
Signif. codes:  0 ‘***’ 0.001 ‘**’ 0.01 ‘*’ 0.05 ‘.’ 0.1 ‘ ’ 1

(Dispersion parameter for binomial family taken to be 1)

    Null deviance: 1320.0  on 1827  degrees of freedom
Residual deviance: 1049.8  on 1820  degrees of freedom
```

```
AIC: 1065.8

Number of Fisher Scoring iterations: 5
```

Rats

```
> summary(bel_ratBEST)

Call:
glm(formula = BEL_RAT ~ TRTRAT_HOM + TRTRAT_DSP + TRTRAT_DAM + 
    TRTRAT_NUM + EXP_RAT + ATT_RAT2, family = binomial, data = cdata, 
    na.action = na.fail)

Deviance Residuals: 
    Min       1Q   Median       3Q      Max  
-3.0777   0.1581   0.3364   0.5894   1.5650  

Coefficients:
              Estimate Std. Error z value Pr(>|z|)    
(Intercept)   -1.38669    0.19798  -7.004 2.49e-12 ***
TRTRAT_HOMYes  0.75264    0.16221   4.640 3.48e-06 ***
TRTRAT_DSPYes  0.57623    0.14622   3.941 8.12e-05 ***
TRTRAT_DAMYes  0.59153    0.18408   3.213  0.00131 ** 
TRTRAT_NUMYes  0.35165    0.18248   1.927  0.05398 .  
EXP_RATYes     1.29213    0.26297   4.914 8.94e-07 ***
ATT_RAT2       0.50994    0.05629   9.059  < 2e-16 ***
---
Signif. codes:  0 ‘***’ 0.001 ‘**’ 0.01 ‘*’ 0.05 ‘.’ 0.1 ‘ ’ 1

(Dispersion parameter for binomial family taken to be 1)

    Null deviance: 1720.3  on 1827  degrees of freedom
Residual deviance: 1345.1  on 1821  degrees of freedom
AIC: 1359.1

Number of Fisher Scoring iterations: 6
```

Gulls

```
> summary(bel_gulBEST)

Call:
glm(formula = BEL_GUL ~ DEM_AGE + DEM_REGCAT + TRTGUL_EAT + TRTGUL_ATP + 
    TRTGUL_DAM + TRTGUL_NUM + EXP_GUL + ATT_GUL2, family = binomial, 
    data = cdata, na.action = na.fail)

Deviance Residuals: 
    Min       1Q   Median       3Q      Max  
-2.3791  -0.7925  -0.5390   0.8745   2.4144  

Coefficients:
                    Estimate Std. Error z value Pr(>|z|)    
(Intercept)        -3.610484   0.282169 -12.795  < 2e-16 ***
DEM_AGE             0.012341   0.003437   3.590  0.00033 ***
DEM_REGCATMEngland -0.244241   0.166131  -1.470  0.14152    
DEM_REGCATNEngland  0.024512   0.140758   0.174  0.86175    
DEM_REGCATNIreland -0.171637   0.564405  -0.304  0.76105    
DEM_REGCATScotland  0.590534   0.203696   2.899  0.00374 ** 
DEM_REGCATWales    -0.176767   0.277350  -0.637  0.52390    
TRTGUL_EATYes       0.369928   0.130389   2.837  0.00455 ** 
TRTGUL_ATPYes       0.546067   0.127122   4.296 1.74e-05 ***
TRTGUL_DAMYes       1.288527   0.156408   8.238  < 2e-16 ***
TRTGUL_NUMYes       0.258546   0.130918   1.975  0.04828 *  
EXP_GULYes          2.313283   0.350898   6.592 4.33e-11 ***
ATT_GUL2            0.489747   0.058301   8.400  < 2e-16 ***
---
Signif. codes:  0 ‘***’ 0.001 ‘**’ 0.01 ‘*’ 0.05 ‘.’ 0.1 ‘ ’ 1

(Dispersion parameter for binomial family taken to be 1)
```

```
    Null deviance: 2400.1  on 1827  degrees of freedom
Residual deviance: 1899.7  on 1815  degrees of freedom
AIC: 1925.7

Number of Fisher Scoring iterations: 5
```

Squirrels

```
> summary(bel_squTOP)

Call:
glm(formula = BEL_SQU ~ DEM_AGE + BEH_GARD + TRTSQU_EAT + TRTSQU_HOM +
    TRTSQU_DAM + TRTSQU_NUM + EXP_SQU + ATT_SQU2, family = binomial,
    data = cdata, na.action = na.fail)

Deviance Residuals:
    Min       1Q   Median       3Q      Max
-2.6383  -0.5478  -0.3989  -0.2674   2.6348

Coefficients:
               Estimate Std. Error z value Pr(>|z|)
(Intercept)   -4.235832   0.307078 -13.794  < 2e-16 ***
DEM_AGE        0.007879   0.004307   1.829  0.06735 .
BEH_GARDYes    0.412229   0.186454   2.211  0.02704 *
TRTSQU_EATYes  0.675053   0.232498   2.903  0.00369 **
TRTSQU_HOMYes  0.873353   0.253118   3.450  0.00056 ***
TRTSQU_DAMYes  1.353685   0.171409   7.897 2.85e-15 ***
TRTSQU_NUMYes  0.765995   0.161343   4.748 2.06e-06 ***
EXP_SQUYes     1.567905   0.268187   5.846 5.03e-09 ***
ATT_SQU2       0.481159   0.067853   7.091 1.33e-12 ***
---
Signif. codes:  0 ‘***’ 0.001 ‘**’ 0.01 ‘*’ 0.05 ‘.’ 0.1 ‘ ’ 1

(Dispersion parameter for binomial family taken to be 1)

    Null deviance: 1941.1  on 1827  degrees of freedom
Residual deviance: 1398.4  on 1819  degrees of freedom
AIC: 1416.4

Number of Fisher Scoring iterations: 5
```

Wasps

```
> summary(bel_wasTOP)

Call:
glm(formula = BEL_WAS ~ TRTWAS_HOM + TRTWAS_ATP + TRTWAS_DSA +
    TRTWAS_ATA + TRTWAS_DAM + TRTWAS_NUM + TRTWAS_FEA + EXP_WAS +
    ATT_WAS2, family = binomial, data = cdata, na.action = na.fail)

Deviance Residuals:
    Min       1Q   Median       3Q      Max
-2.8692  -0.8571   0.3640   0.8373   2.0000

Coefficients:
              Estimate Std. Error z value Pr(>|z|)
(Intercept)   -2.37564    0.20340 -11.680  < 2e-16 ***
TRTWAS_HOMYes  0.70571    0.13679   5.159 2.48e-07 ***
TRTWAS_ATPYes  0.53477    0.12496   4.280 1.87e-05 ***
TRTWAS_DSAYes -1.32230    0.61897  -2.136  0.03266 *
TRTWAS_ATAYes  0.85470    0.34706   2.463  0.01379 *
TRTWAS_DAMYes  0.70533    0.21361   3.302  0.00096 ***
TRTWAS_NUMYes  0.52845    0.26715   1.978  0.04792 *
TRTWAS_FEAYes  0.43174    0.15139   2.852  0.00435 **
EXP_WASYes     1.48496    0.14564  10.196  < 2e-16 ***
ATT_WAS2       0.52109    0.05551   9.387  < 2e-16 ***
---
Signif. codes:  0 ‘***’ 0.001 ‘**’ 0.01 ‘*’ 0.05 ‘.’ 0.1 ‘ ’ 1
```

```
(Dispersion parameter for binomial family taken to be 1)

    Null deviance: 2392.2  on 1827  degrees of freedom
Residual deviance: 1802.0  on 1818  degrees of freedom
AIC: 1822

Number of Fisher Scoring iterations: 5
```

Document S5. Model summaries for models of use of negative language (pest or vermin) for species that they believe cause people problems generally in the UK.

```
Badgers
> summary(pesver_badgerBEST)
formula: PESVER_BAD ~ TRTBAD_DSP + TRTBAD_ATP + TRTBAD_DSA + TRTBAD_NUM
data:    cdata

 link  threshold nobs logLik  AIC    niter max.grad cond.H
 logit flexible  298  -309.43 632.86 6(0)  5.76e-07 2.2e+01

Coefficients:
              Estimate Std. Error z value Pr(>|z|)
TRTBAD_DSPYes   0.6391     0.2973   2.150 0.031555 *
TRTBAD_ATPYes   0.8057     0.3707   2.174 0.029728 *
TRTBAD_DSAYes   0.7826     0.2376   3.294 0.000987 ***
TRTBAD_NUMYes   0.8089     0.2755   2.936 0.003326 **
---
Signif. codes:  0 '***' 0.001 '**' 0.01 '*' 0.05 '.' 0.1 ' ' 1

Threshold coefficients:
           Estimate Std. Error z value
NoPV|Pest    0.3923     0.1653   2.373
Pest|Verm    2.6719     0.2376  11.247
Verm|PandV   3.2392     0.2676  12.105


Foxes
> summary(pesver_foxTOP)
formula: PESVER_FOX ~ TRTFOX_DSP + TRTFOX_ATP + TRTFOX_DSA + TRTFOX_DAM + TRTFOX_
NUM
data:    cdata

 link  threshold nobs logLik  AIC     niter max.grad cond.H
 logit flexible  655  -725.48 1466.95 5(0)  3.55e-07 1.8e+01

Coefficients:
              Estimate Std. Error z value Pr(>|z|)
TRTFOX_DSPYes   0.5448     0.2422   2.249 0.024483 *
TRTFOX_ATPYes   0.3957     0.1944   2.035 0.041821 *
TRTFOX_DSAYes   0.5242     0.1950   2.688 0.007185 **
TRTFOX_DAMYes   0.6600     0.1735   3.803 0.000143 ***
TRTFOX_NUMYes   1.2523     0.1930   6.489 8.66e-11 ***
---
Signif. codes:  0 '***' 0.001 '**' 0.01 '*' 0.05 '.' 0.1 ' ' 1

Threshold coefficients:
           Estimate Std. Error z value
NoPV|Pest   -0.1391     0.1028  -1.353
Pest|Verm    2.2224     0.1427  15.579
Verm|PandV   3.0491     0.1693  18.015


Moles
> summary(pesver_molBEST)
formula: PESVER_MOL ~ TRTMOL_EAT + TRTMOL_DAM + TRTMOL_NUM + TRTMOL_FEA
data:    cdata

 link  threshold nobs logLik  AIC     niter max.grad cond.H
 logit flexible  645  -627.03 1268.05 6(0)  1.24e-10 6.7e+01

Coefficients:
              Estimate Std. Error z value Pr(>|z|)
TRTMOL_EATYes   2.5545     0.5509   4.637 3.53e-06 ***
TRTMOL_DAMYes   0.9391     0.1707   5.501 3.78e-08 ***
TRTMOL_NUMYes   1.0807     0.3727   2.899  0.00374 **
TRTMOL_FEAYes   2.6499     0.6063   4.371 1.24e-05 ***
---
```

```
Signif. codes:  0 ‘***’ 0.001 ‘**’ 0.01 ‘*’ 0.05 ‘.’ 0.1 ‘ ’ 1

Threshold coefficients:
           Estimate Std. Error z value
NoPV|Pest   -0.3441     0.1358  -2.535
Pest|Verm    2.7631     0.1842  15.005
Verm|PandV   3.7769     0.2276  16.593
```

Mice

```
> summary(pesver_mouTOP)
formula: PESVER_MOU ~ TRTMOU_DSP + TRTMOU_DSA + TRTMOU_DAM + TRTMOU_NUM + TRTMOU_
FEA
data:    cdata

 link  threshold nobs logLik   AIC     niter max.grad cond.H
 logit flexible  1195 -1475.32 2966.65 5(0)  2.25e-09 2.4e+01

Coefficients:
              Estimate Std. Error z value Pr(>|z|)
TRTMOU_DSPYes   0.7248     0.1264   5.736 9.70e-09 ***
TRTMOU_DSAYes   0.3608     0.1453   2.483  0.01304 *
TRTMOU_DAMYes   0.3397     0.1121   3.029  0.00245 **
TRTMOU_NUMYes   0.6943     0.1490   4.660 3.16e-06 ***
TRTMOU_FEAYes   0.5374     0.2054   2.616  0.00889 **
---
Signif. codes:  0 ‘***’ 0.001 ‘**’ 0.01 ‘*’ 0.05 ‘.’ 0.1 ‘ ’ 1

Threshold coefficients:
           Estimate Std. Error z value
NoPV|Pest  -1.80577    0.11383 -15.863
Pest|Verm   0.12798    0.08585   1.491
Verm|PandV  1.42531    0.09565  14.901
```

Pigeons

```
> summary(pesver_pigBEST)
formula: PESVER_PIG ~ TRTPIG_DSP + TRTPIG_DSA + TRTPIG_DAM + TRTPIG_NUM + TRTPIG_
FEA
data:    cdata

 link  threshold nobs logLik  AIC     niter max.grad cond.H
 logit flexible  741  -904.28 1824.56 5(0)  8.61e-08 7.8e+01

Coefficients:
              Estimate Std. Error z value Pr(>|z|)
TRTPIG_DSPYes   0.6781     0.1486   4.564 5.01e-06 ***
TRTPIG_DSAYes   0.4831     0.1644   2.939  0.00329 **
TRTPIG_DAMYes   0.2864     0.1447   1.980  0.04772 *
TRTPIG_NUMYes   0.6069     0.1409   4.308 1.65e-05 ***
TRTPIG_FEAYes   0.6937     0.4041   1.717  0.08602 .
---
Signif. codes:  0 ‘***’ 0.001 ‘**’ 0.01 ‘*’ 0.05 ‘.’ 0.1 ‘ ’ 1

Threshold coefficients:
           Estimate Std. Error z value
NoPV|Pest   -1.5097     0.1446 -10.443
Pest|Verm    0.7299     0.1228   5.943
Verm|PandV   1.5783     0.1324  11.925
```

Rabbits

```
> summary(pesver_rabBEST)
formula: PESVER_RAB ~ TRTRAB_DSP + TRTRAB_DAM + TRTRAB_NUM
data:    cdata

 link  threshold nobs logLik  AIC    niter max.grad cond.H
 logit flexible  226  -218.18 448.35 6(0)  1.45e-10 2.6e+01
```

```
Coefficients:
                Estimate Std. Error z value Pr(>|z|)
TRTRAB_DSPYes   1.8726     0.5884   3.182  0.00146 **
TRTRAB_DAMYes   0.8916     0.2862   3.115  0.00184 **
TRTRAB_NUMYes   0.7873     0.3356   2.346  0.01897 *
---
Signif. codes:  0 ‘***’ 0.001 ‘**’ 0.01 ‘*’ 0.05 ‘.’ 0.1 ‘ ’ 1

Threshold coefficients:
           Estimate Std. Error z value
NoPV|Pest   -0.2678     0.1939  -1.381
Pest|Verm    2.8480     0.2955   9.639
Verm|PandV   3.6041     0.3490  10.326
```

Rats

```
> summary(pesver_ratBEST)
formula: PESVER_RAT ~ TRTRAT_DSP + TRTRAT_DSA + TRTRAT_DAM + TRTRAT_NUM + TRTRAT_
FEA
data:    cdata

 link  threshold nobs logLik   AIC     niter max.grad cond.H
 logit flexible  1622 -1696.63 3409.26 5(0)  7.13e-09 2.9e+01

Coefficients:
                Estimate Std. Error z value Pr(>|z|)
TRTRAT_DSPYes   0.3761     0.1101   3.415 0.000638 ***
TRTRAT_DSAYes   0.2471     0.1061   2.328 0.019916 *
TRTRAT_DAMYes   0.3021     0.1090   2.773 0.005562 **
TRTRAT_NUMYes   0.7878     0.1089   7.234 4.68e-13 ***
TRTRAT_FEAYes   0.6220     0.1315   4.730 2.24e-06 ***
---
Signif. codes:  0 ‘***’ 0.001 ‘**’ 0.01 ‘*’ 0.05 ‘.’ 0.1 ‘ ’ 1

Threshold coefficients:
           Estimate Std. Error z value
NoPV|Pest  -2.01173    0.12690 -15.853
Pest|Verm  -0.84790    0.09980  -8.496
Verm|PandV  1.06764    0.09984  10.694
```

Gulls

```
> summary(pesver_gulBEST)
formula: PESVER_GUL ~ TRTGUL_EAT + TRTGUL_DSP + TRTGUL_DSA + TRTGUL_NUM
data:    cdata

 link  threshold nobs logLik  AIC     niter max.grad cond.H
 logit flexible  713  -754.08 1522.16 6(1)  3.97e-08 6.4e+01

Coefficients:
                Estimate Std. Error z value Pr(>|z|)
TRTGUL_EATYes   0.2973     0.1532   1.941  0.05232 .
TRTGUL_DSPYes   0.6818     0.2383   2.861  0.00422 **
TRTGUL_DSAYes   0.4638     0.2425   1.913  0.05581 .
TRTGUL_NUMYes   0.9480     0.1557   6.089 1.14e-09 ***
---
Signif. codes:  0 ‘***’ 0.001 ‘**’ 0.01 ‘*’ 0.05 ‘.’ 0.1 ‘ ’ 1

Threshold coefficients:
           Estimate Std. Error z value
NoPV|Pest   -1.5179     0.1361  -11.15
Pest|Verm    1.4577     0.1296   11.25
Verm|PandV   1.8414     0.1364   13.50
```

Squirrels

```
> summary(pesver_squTOP)
formula: PESVER_SQU ~ TRTSQU_DSA + TRTSQU_DAM + TRTSQU_NUM
data:    cdata
```

```
 link  threshold nobs logLik  AIC     niter max.grad cond.H
 logit flexible  430  -520.40 1052.79 5(0)  1.12e-08 2.3e+01

Coefficients:
              Estimate Std. Error z value Pr(>|z|)
TRTSQU_DSAYes   1.3354     0.2773   4.816 1.47e-06 ***
TRTSQU_DAMYes   0.5518     0.1840   3.000   0.0027 **
TRTSQU_NUMYes   1.1070     0.1895   5.843 5.12e-09 ***
---
Signif. codes:  0 ‘***’ 0.001 ‘**’ 0.01 ‘*’ 0.05 ‘.’ 0.1 ‘ ’ 1

Threshold coefficients:
           Estimate Std. Error z value
NoPV|Pest   -1.0603     0.1641  -6.461
Pest|Verm    1.1720     0.1609   7.286
Verm|PandV   2.0535     0.1805  11.374


Wasps
> summary(pesver_wasBEST)
formula: PESVER_WAS ~ TRTWAS_DSP + TRTWAS_ATA + TRTWAS_NUM + TRTWAS_FEA
data:    cdata

 link  threshold nobs logLik   AIC     niter max.grad cond.H
 logit flexible  1253 -1037.60 2089.20 7(1)  3.63e-12 1.9e+02

Coefficients:
              Estimate Std. Error z value Pr(>|z|)
TRTWAS_DSPYes   1.1605     0.4045   2.869  0.00412 **
TRTWAS_ATAYes   0.4110     0.2096   1.961  0.04989 *
TRTWAS_NUMYes   1.1064     0.1702   6.502 7.92e-11 ***
TRTWAS_FEAYes   0.4055     0.1387   2.925  0.00345 **
---
Signif. codes:  0 ‘***’ 0.001 ‘**’ 0.01 ‘*’ 0.05 ‘.’ 0.1 ‘ ’ 1

Threshold coefficients:
           Estimate Std. Error z value
NoPV|Pest  -1.65679    0.09246  -17.92
Pest|Verm   2.18559    0.10600   20.62
Verm|PandV  2.44128    0.11273   21.66
```

Document S6. Model summaries for models of past control by people that have experienced problems with species personally. Past control could not be modelled for badgers, foxes, pigeons, rabbits, gulls or squirrels.

Moles

```
> summary(pascon_molTOP)

Call:
glm(formula = PASCON_MOL ~ DEM_SOCGRADE2 + HOME_TYPE2 + ATT_MOL2,
    family = binomial, data = cdata, na.action = na.fail)

Deviance Residuals:
    Min       1Q   Median       3Q      Max
-2.1576  -0.9862   0.4530   0.9491   2.1516

Coefficients:
                  Estimate Std. Error z value Pr(>|z|)
(Intercept)        -2.0487     0.9231  -2.219  0.02646 *
DEM_SOCGRADE2      -0.5206     0.2298  -2.265  0.02349 *
HOME_TYPE2Other     2.7040     1.4787   1.829  0.06745 .
HOME_TYPE2Detach    1.8255     0.6214   2.938  0.00331 **
HOME_TYPE2Flamai    1.4783     0.9092   1.626  0.10398
HOME_TYPE2Semidet   0.8061     0.6678   1.207  0.22740
HOME_TYPE2Terrace   2.0622     0.8917   2.313  0.02075 *
ATT_MOL2            0.5937     0.1916   3.099  0.00194 **
---
Signif. codes:  0 ‘***’ 0.001 ‘**’ 0.01 ‘*’ 0.05 ‘.’ 0.1 ‘ ’ 1

(Dispersion parameter for binomial family taken to be 1)

    Null deviance: 159.35  on 114  degrees of freedom
Residual deviance: 130.87  on 107  degrees of freedom
AIC: 146.87

Number of Fisher Scoring iterations: 4
```

Mice

```
> summary(pascon_mouBEST)

Call:
glm(formula = PASCON_MOU ~ HOME_TENU2 + TRTMOU_HOM + ATT_MOU2,
    family = binomial, data = cdata, na.action = na.fail)

Deviance Residuals:
    Min       1Q   Median       3Q      Max
-2.4832   0.3158   0.4320   0.5628   1.2553

Coefficients:
                   Estimate Std. Error z value Pr(>|z|)
(Intercept)         -0.6197     0.4990  -1.242 0.214314
HOME_TENU2Other     -0.8355     0.7066  -1.182 0.237041
HOME_TENU2Own        0.9594     0.3954   2.426 0.015258 *
HOME_TENU2Social     0.8964     0.5729   1.565 0.117629
HOME_TENU2Rentpriv   0.4123     0.4643   0.888 0.374562
TRTMOU_HOMYes        0.9183     0.2632   3.489 0.000484 ***
ATT_MOU2             0.3557     0.1002   3.551 0.000384 ***
---
Signif. codes:  0 ‘***’ 0.001 ‘**’ 0.01 ‘*’ 0.05 ‘.’ 0.1 ‘ ’ 1

(Dispersion parameter for binomial family taken to be 1)

    Null deviance: 461.78  on 567  degrees of freedom
Residual deviance: 419.73  on 561  degrees of freedom
AIC: 433.73

Number of Fisher Scoring iterations: 5
```

Rats

```
> summary(pascon_ratBEST)

Call:
glm(formula = PASCON_RAT ~ DEM_AGE + BEH_PETS + TRTRAT_DSA +
    TRTRAT_FEA, family = binomial, data = cdata, na.action = na.fail)

Deviance Residuals:
    Min       1Q   Median       3Q      Max
-2.6165   0.3528   0.4622   0.6002   1.0715

Coefficients:
               Estimate Std. Error z value Pr(>|z|)
(Intercept)    0.565825   0.490899   1.153  0.24906
DEM_AGE        0.026932   0.008698   3.096  0.00196 **
BEH_PETSYes   -0.722149   0.291706  -2.476  0.01330 *
TRTRAT_DSAYes  0.696362   0.291461   2.389  0.01688 *
TRTRAT_FEAYes -0.801274   0.316888  -2.529  0.01145 *
---
Signif. codes:  0 ‘***’ 0.001 ‘**’ 0.01 ‘*’ 0.05 ‘.’ 0.1 ‘ ’ 1

(Dispersion parameter for binomial family taken to be 1)

    Null deviance: 345.20  on 410  degrees of freedom
Residual deviance: 321.32  on 406  degrees of freedom
AIC: 331.32

Number of Fisher Scoring iterations: 5
```

Wasps

```
> summary(pascon_wasTOP)

Call:
glm(formula = PASCON_WAS ~ HOME_TYPE2 + BEH_GARD + TRTWAS_EAT +
    TRTWAS_HOM + TRTWAS_DAM, family = binomial, data = cdata,
    na.action = na.fail)

Deviance Residuals:
    Min       1Q   Median       3Q      Max
-2.6051   0.3326   0.5238   0.7391   1.1599

Coefficients:
                  Estimate Std. Error z value Pr(>|z|)
(Intercept)        0.07527    0.44149   0.171  0.86462
HOME_TYPE2Other    1.65069    1.14740   1.439  0.15025
HOME_TYPE2Detach   1.04128    0.39708   2.622  0.00873 **
HOME_TYPE2Flamai   0.09663    0.43015   0.225  0.82226
HOME_TYPE2Semidet  0.28213    0.35646   0.791  0.42867
HOME_TYPE2Terrace -0.03387    0.36982  -0.092  0.92702
BEH_GARDYes        0.80062    0.31647   2.530  0.01141 *
TRTWAS_EATYes     -1.18193    0.38331  -3.083  0.00205 **
TRTWAS_HOMYes      0.49213    0.21813   2.256  0.02406 *
TRTWAS_DAMYes      0.94988    0.30354   3.129  0.00175 **
---
Signif. codes:  0 ‘***’ 0.001 ‘**’ 0.01 ‘*’ 0.05 ‘.’ 0.1 ‘ ’ 1

(Dispersion parameter for binomial family taken to be 1)

    Null deviance: 652.84  on 648  degrees of freedom
Residual deviance: 600.36  on 639  degrees of freedom
AIC: 620.36

Number of Fisher Scoring iterations: 5
```

Table S1. The twelve UK Government Office Regions (GORs), percentage of respondents living in each GOR and the geographical areas the GORs were allocated to for analytical purposes.

Government Office Region (GOR)	Percentage of respondents (%)	Geographical area
London	12.8	Southern England
South East England	13.7	Southern England
South West England	8.7	Southern England
East of England	9.5	Southern England
West Midlands	8.9	Middle England
East Midlands	7.3	Middle England
North East England	4.1	Northern England
North West England	11.4	Northern England
Yorkshire and the Humber	8.5	Northern England
Wales	4.9	Wales
Scotland	8.8	Scotland
Northern Ireland	1.4	Northern Ireland

Table S2. Abbreviations used in Tables 4-8 for factors potentially associated with measures of pest status and demand for pest control.

DATA TYPE	DATA	DESCRIPTION
DEMOGRAPHY	GENDER (M)	RESPONDENT IS MALE (RATHER THAN FEMALE)
	INCREASING AGE	RESPONDENT'S INCREASING AGE
	SOCIAL GRADE (WORKING CLASS)	RESPONDENT'S DECREASING SOCIAL GRADE (FROM 'AB' TO 'DE')
	URBAN OR FRINGE	RESPONDENT LIVES IN URBAN OR FRINGE (RATHER THAN RURAL) AREA
	REGION	RESPONDENT'S HOME REGION (ONE OF SIX)
HOME	HOME TYPE	RESPONDENT'S HOME TYPE (ONE OF SIX GROUPS)
	OLDER HOME	RESPONDENT'S HOME AGE (ONE OF FIVE PERIODS)
	HOME TENURE	RESPONDENT'S HOME TENURE (ONE OF FIVE GROUPS)
HUMAN BEHAVIOUR	CHILDREN	RESPONDENT HAS CHILDREN (0-16 YEARS) LIVING WITH THEM
	GARDEN	RESPONDENT HAS GARDEN
	CATS/DOGS	RESPONDENT KEEPS CAT(S) AND/OR DOG(S)
	POULTRY/OD PETS	RESPONDENT KEEPS PETS OR DOMESTIC POULTRY IN OUTDOOR PENS
	FEEDSTORE	RESPONDENT USES AN OUTDOOR STORE FOR ANIMAL FEED
	FEED BIRDS/ANIMS	RESPONDENT FEEDS WILD BIRDS OR ANIMALS IN THEIR GARDEN
	COMPOST	RESPONDENT MAKES THEIR OWN COMPOST
	FOOD WASTE	RESPONDENT STORES FOOD WASTE IN AN OUTDOOR BIN FOR COUNCIL
PERCEIVED SPECIES TRAITS	EAT OUR FOOD	RESPONDENT BELIEVES SPECIES EATS PEOPLE'S FOOD
	ENTER HOMES	RESPONDENT BELIEVES SPECIES ENTERS PEOPLE'S HOMES
	DISEASE TO PEOPLE	RESPONDENT BELIEVES SPECIES SPREADS DISEASE TO PEOPLE
	ATTACK PEOPLE	RESPONDENT BELIEVES SPECIES ATTACKS PEOPLE
	DISEASE TO PETS / POUL	RESPONDENT BELIEVES SPECIES SPREADS DISEASE TO PETS OR DOMESTIC POULTRY
	ATTACK PETS / POUL	RESPONDENT BELIEVES SPECIES ATTACKS PETS OR DOMESTIC POULTRY
	DAMAGE	RESPONDENT BELIEVES SPECIES DAMAGES PEOPLE'S PROPERTY
	NUMBERS	RESPONDENT BELIEVES SPECIES NUMBERS ARE OUT OF CONTROL
	FEAR	RESPONDENT IS AFRAID OF SPECIES
PERCEPTIONS ABOUT SPECIES	EXPERIENCE	RESPONDENT HAS EXPERIENCED PERSONAL PROBLEMS WITH SPECIES
	BELIEF	RESPONDENT BELIEVES SPECIES CAUSES PROBLEMS FOR PEOPLE GENERALLY IN UK
	NEG ATTITUDE	RESPONDENT HAS A NEGATIVE ATTITUDE TOWARDS SPECIES

Table S3: Mean net loss per UK council for 2013/14 (n=258), 2014/15 (n=264), 2015/16 (n=267) and 2016/17 (n=263), estimated total net loss for all UK councils reporting to have provided a public pest control service in 2013/14 (n=299), 2014/15 (n=300), 2015/16 (n=299) and 2016/17 (n=299), and mean net loss per 1,000 households per UK council for 2013/14 (n=258), 2014/15 (n=264), 2015/16 (n=267) and 2016/17 (n=263).

	2013/14	2014/15	2015/16	2016/17
**Mean net loss per UK council**	£73.7k	£61.8k	£58.5k	£48.3k
**Estimated total net loss for all UK councils reporting to have provided a public service**	£22.0M	£18.5M	£17.5M	£14.4M
**Mean net loss per 1,000 households per UK council**	£1.28k	£1.10k	£1.06k	£0.817k

Table S4: Total number of treatment requests per species per annum (bracketed figures show proportion per species out of the total number of treatment requests received per annum).

Species	2013/14	2014/15	2015/16	2016/17
**Rats**	197k (47.5%)	249k (50.1%)	231k (52.0%)	224k (48.3%)
**Mice**	110k (26.5%)	126k (25.3%)	125k (28.1%)	126k (27.1%)
**Wasps**	101k (24.3%)	114k (22.9%)	80.0k (18.0%)	107k (23.1%)
**Squirrels**	2.75k (0.7%)	3.07k (0.6%)	3.30k (0.7%)	2.90k (0.6%)
**Gulls**	2.04k (0.5%)	3.14k (0.6%)	2.32k (0.5%)	2.01k (0.4%)
**Pigeons**	1.68k (0.4%)	1.73k (0.3%)	1.75k (0.4%)	1.57k (0.3%)
**Moles**	410 (0.1%)	540 (0.1%)	546 (0.1%)	621 (0.1%)
**Foxes**	135 (0.0%)	103 (0.0%)	90 (0.0%)	137 (0.0%)

Table S5: Mean annual number of treatment requests per species per UK council treating for that species (bracketed figures show mean annual number of treatment requests per species per 1,000 households per UK council treating for that species; square-bracketed figures below show sample sizes).

Species	2013/14	2014/15	2015/16	2016/17
**Rats**	784 (10.1)<br>[n=251]	939 (12.3)<br>[n=265]	848 (11.2)<br>[n=272]	816 (10.9)<br>[n=275]
**Mice**	438 (5.0)<br>[n=250]	476 (5.6)<br>[n=263]	463 (5.2)<br>[n=269]	462 (5.0)<br>[n=272]
**Wasps**	409 (5.8)<br>[n=246]	437 (6.1)<br>[n=260]	300 (4.6)<br>[n=267]	398 (6.0)<br>[n=270]
**Gulls**	70 (1.0)<br>[n=29]	105 (1.6)<br>[n=30]	77 (1.2)<br>[n=30]	65 (1.3)<br>[n=31]
**Squirrels**	21 (0.3)<br>[n=130]	23 (0.3)<br>[n=136]	24 (0.3)<br>[n=140]	19 (0.2)<br>[n=152]
**Pigeons**	22 (0.2)<br>[n=76]	22 (0.2)<br>[n=77]	22 (0.2)<br>[n=78]	20 (0.2)<br>[n=80]
**Moles**	9 (0.1)<br>[n=45]	11 (0.2)<br>[n=49]	11 (0.2)<br>[n=52]	11 (0.2)<br>[n=57]
**Foxes**	6 (0.1)<br>[n=21]	5 (0.1)<br>[n=20]	5 (0.0)<br>[n=20]	6 (0.1)<br>[n=22]

Table S6. Infographic showing the seven principles for ethical wildlife management (adapted from [38]).

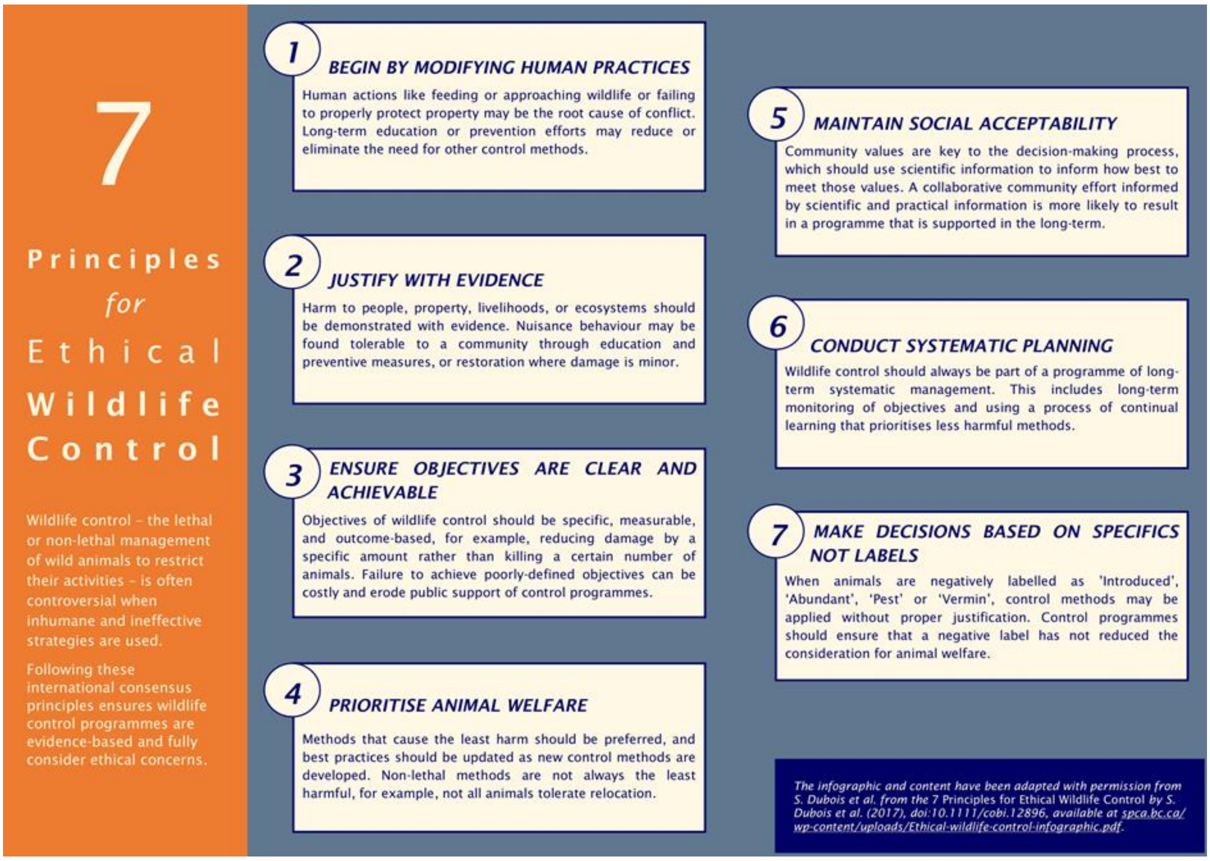

Figure S1. Respondent home type (percentage of respondents). Types are: Detached = Detached house, Semi-detached = Semi-detached house, Terraced = Terraced house, Flat/Maisonette = Studio, flat or maisonette, Bungalow = Bungalow, Other = Static caravan, any other type, don't know or prefer not to say.

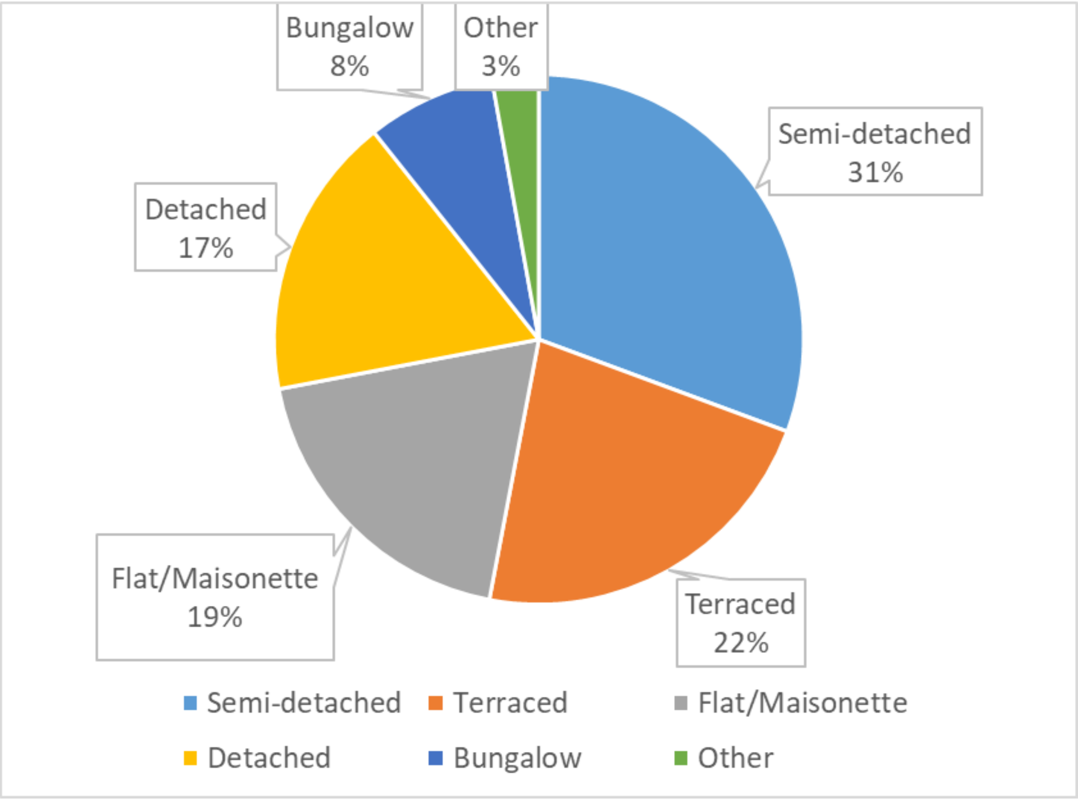

Figure S2. Respondent home age (percentage of respondents). Age categories are: PreVic = Pre-Victorian (before 1836), Victorian = 1837-1901, PreWW2 = Pre-World War II (1902-1945), PostWW2 = Post-World War II (1946-2000), 21stC = 21st Century (since 2001), DK = Don't know.

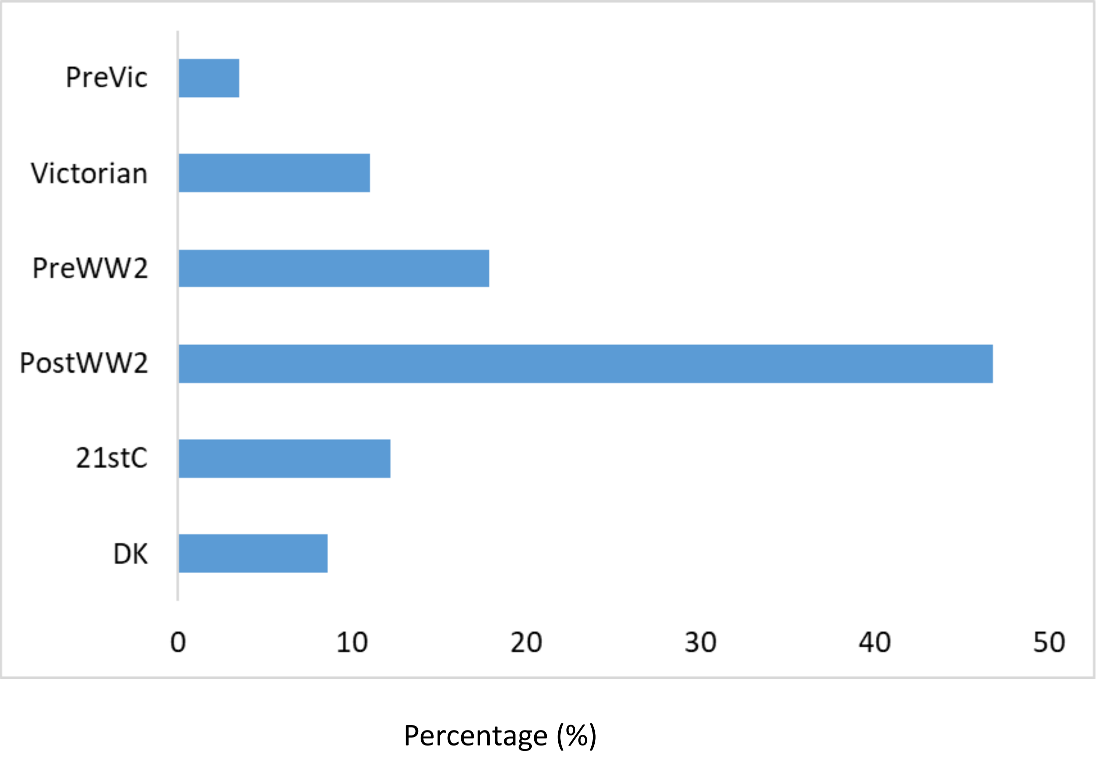

Figure S3. Respondent home tenure (percentage of respondents). Tenure categories are: Own or part own = Owns home outright or part owns with mortgage or shared ownership scheme, Rented private = Rents home from private landlord, Social housing = rents home from local authority or housing association, Live with family/friends = lives with parents, family or friends either rent-free or paying some rent, Other = Any other arrangement.

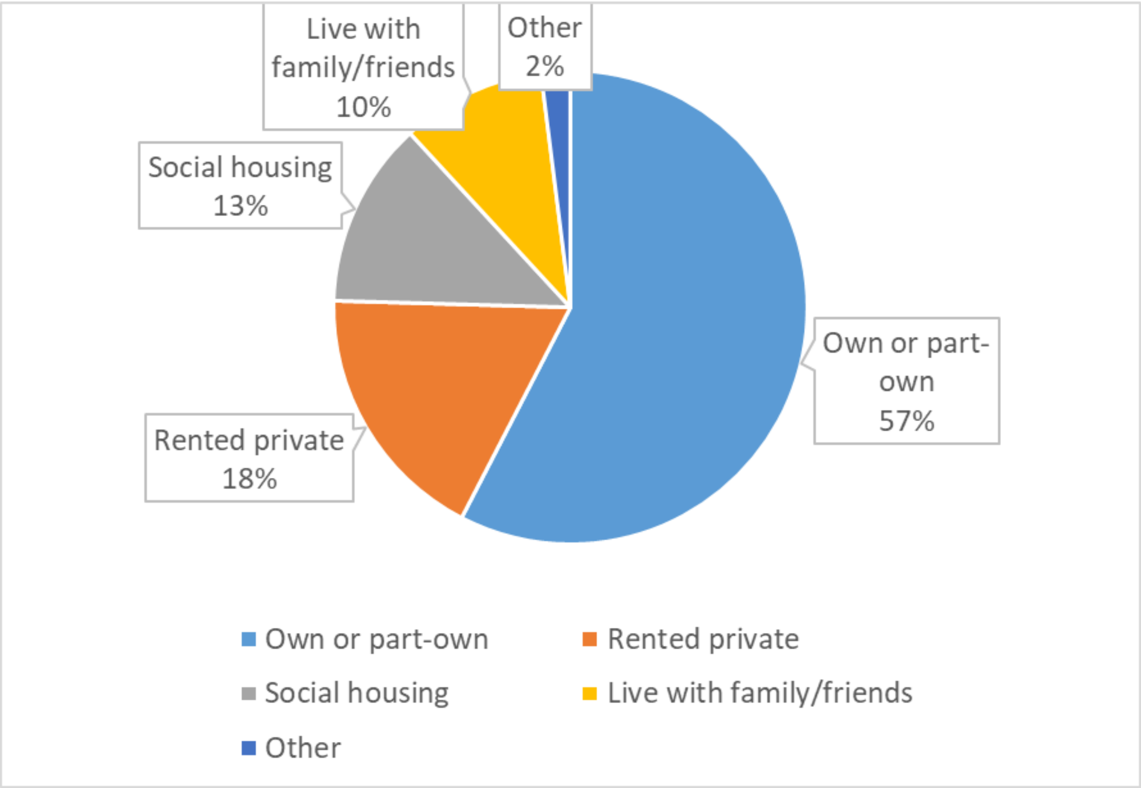

Figure S4. Respondent behaviours (percentage of respondents). Behaviours are: Children = Has any children (0-16 years) living with them, Garden = Has a garden, Cats/dogs = Keeps any cats or dogs, Outdoor poultry/pets = Keeps any pets or domestic poultry in outdoor pens/aviaries in their garden (e.g. rabbits/guinea pigs/chickens/ducks / other birds), Feed store = Uses an outdoor store for animal feed, Feed birds/animals = Feeds wild birds or animals in their garden, Make compost = Makes their own compost, Store food waste = Stores food waste in an outdoor bin for collection by the council, None = None of the above.

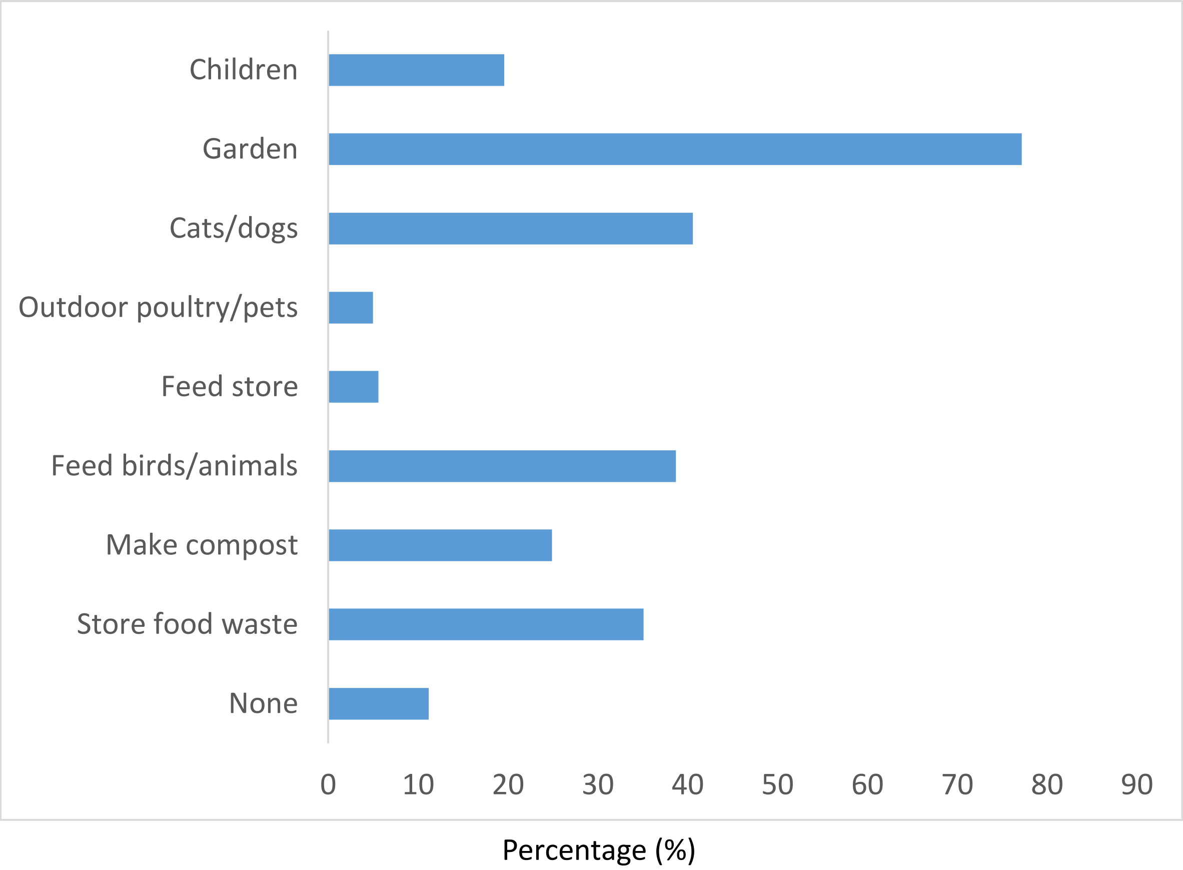

Figure S5. Respondent attitudes towards each species (percentage of respondents): Vneg = 'Very negative', Sneg = 'Somewhat negative', Neut = 'Neutral', Spos = 'Somewhat positive', Vpos = 'Very positive'.

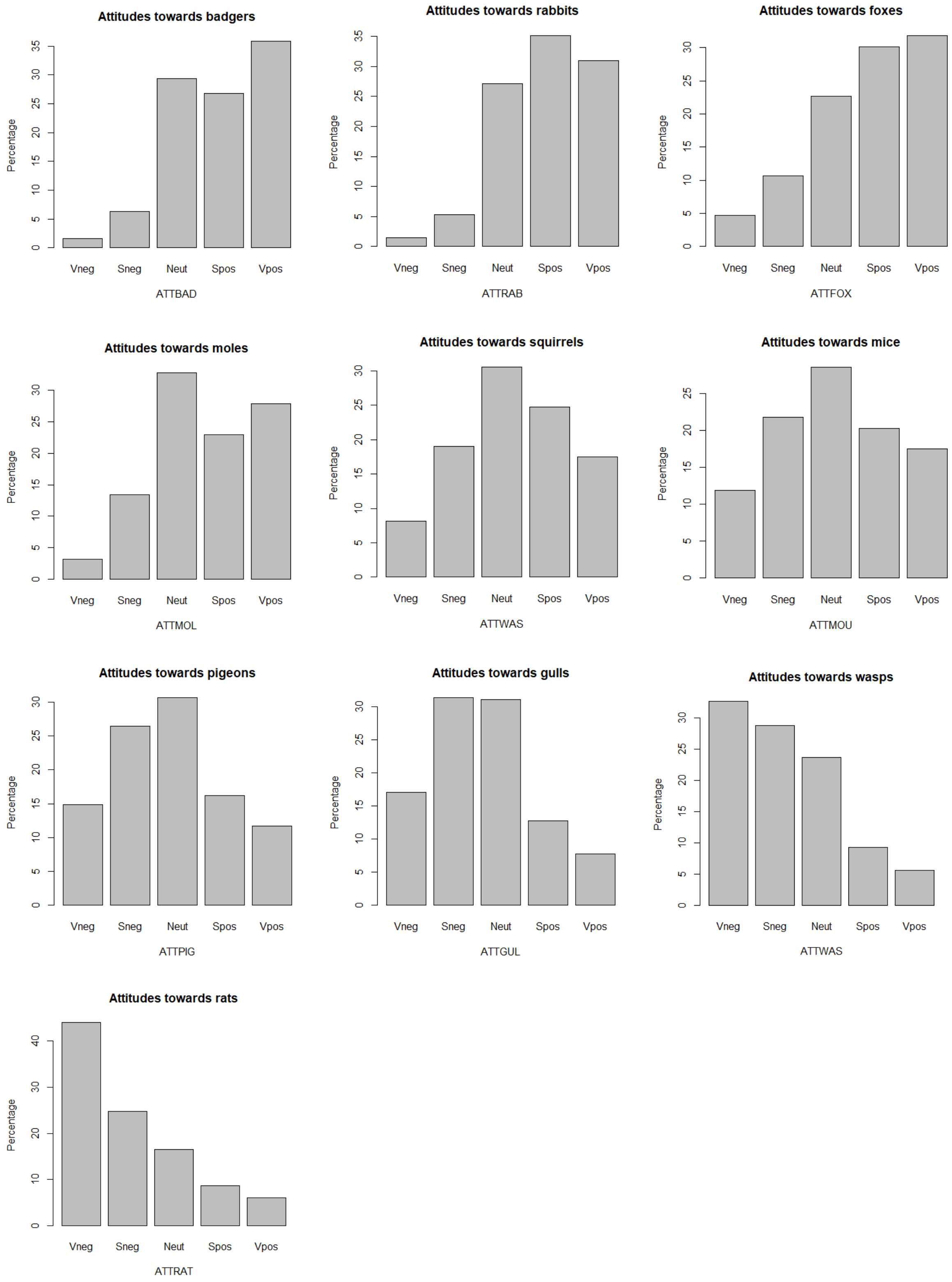

Figure S6. Use of negative language towards each species by people believing that a particular species causes problems for people generally in the UK (percentage of respondents believing): Verm = 'vermin', PandV = 'pest and vermin', Pest = pest, NoPV = 'neither pest not vermin'.

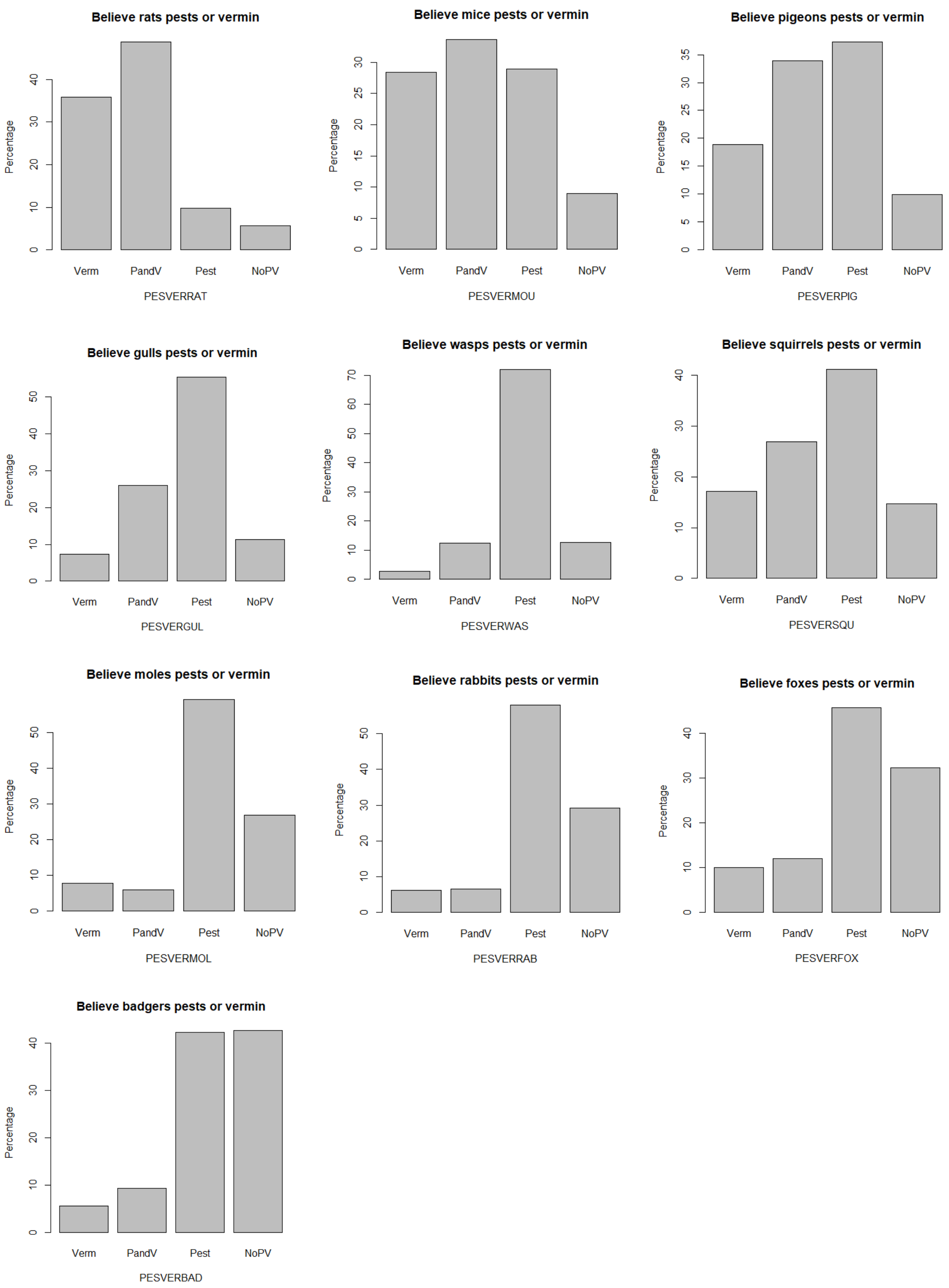

Figure S7. Traits attributed to the ten species by species (percentage of people). The traits are: F = 'They eat our food', H = 'They come into our homes', DP = 'They spread disease to people', AP = 'They attack people', DA = 'They spread disease to pets / domestic poultry etc.', AA = 'They attack pets / domestic poultry etc.', D = 'They damage our property', Nu = 'Their numbers are out of control', A = 'I'm afraid of them', No='None of the above'.

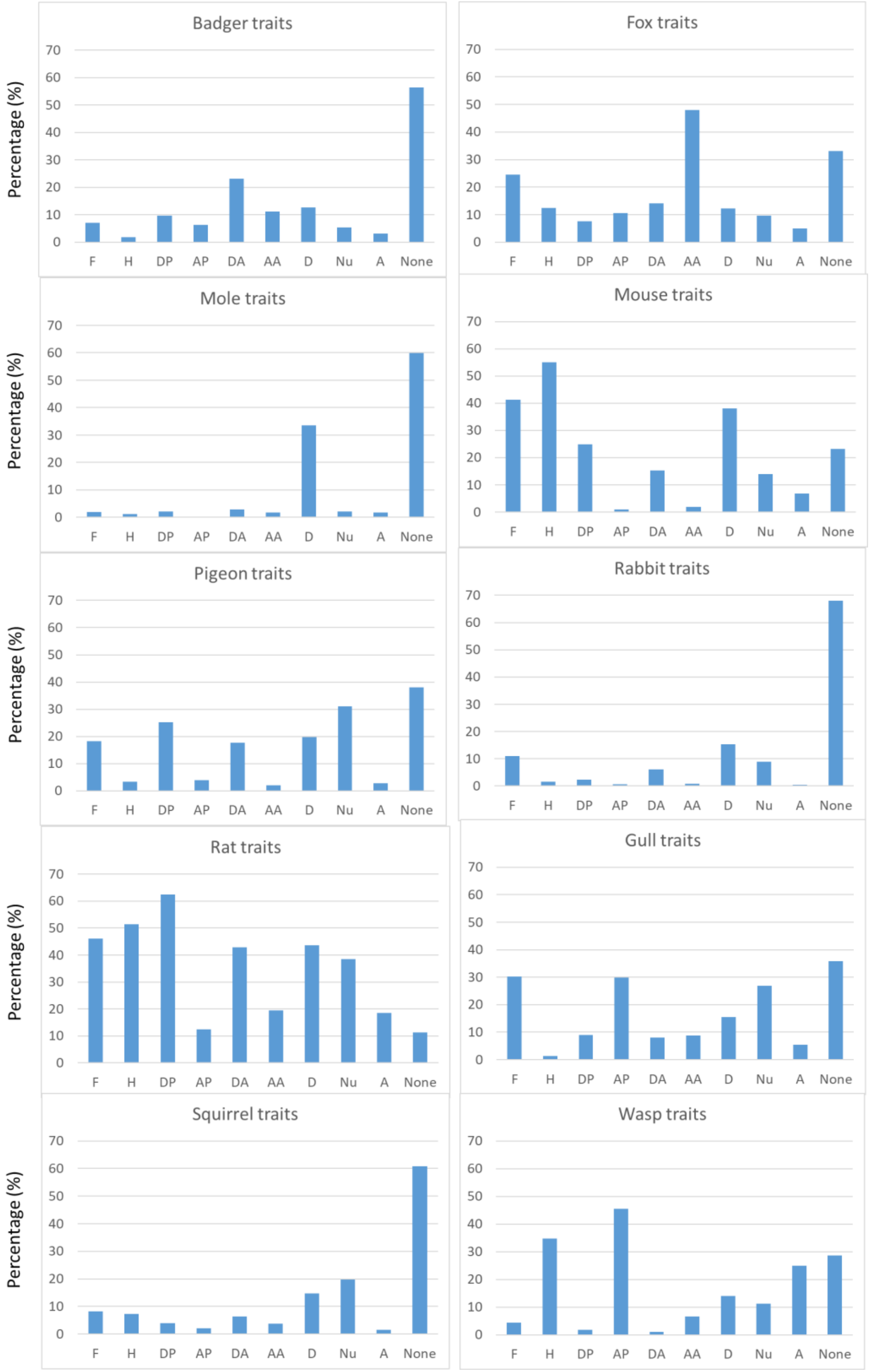

Figure S8. Traits attributed to the ten species by trait (percentage of people). The species are: B = Badger, F = Fox, Ml = Mole, Mo = Mouse, P = Pigeon, Rb = Rabbit, Rt = Rat, G = Gull, S = Squirrel, W = Wasp.

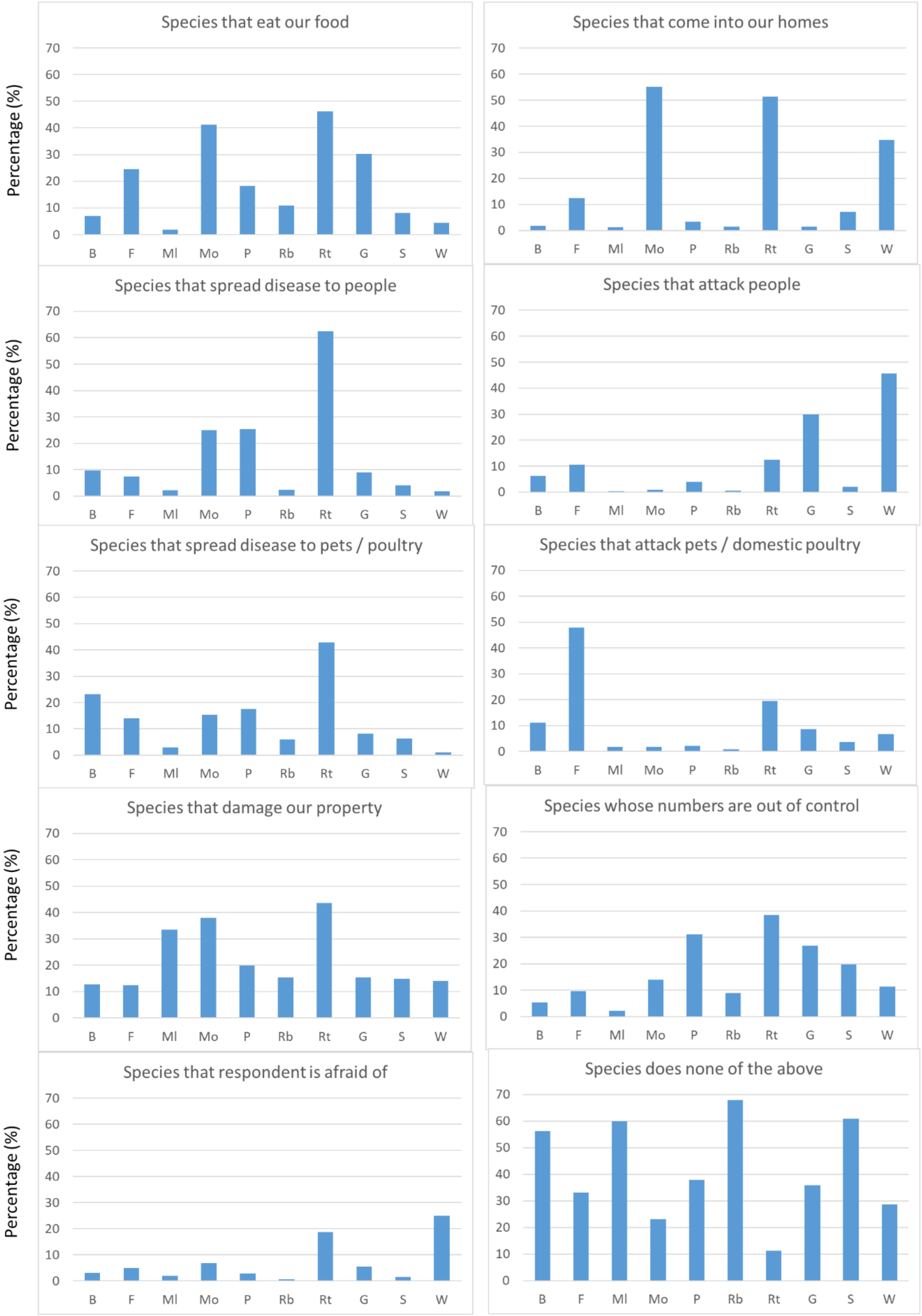

Figure S9. Drivers of respondents' attitudes towards each species (marginal effects in logistic regression models): (a) badgers; (b) moles; (c) mice; (d) pigeons; (e) rabbits; (f) rats; (g) gulls; (h) wasps. Blue = very positive, pink = somewhat positive, green = neutral, red = somewhat negative, yellow = very negative. 'Believe numbers' = Believe numbers are out of control. Home age categories are: 1 = 21st Century (≥2001), 2 = Post World War II (1946-2000), 3 = Pre-World War II (1902-1945), 4 = Victorian (1837-1901), 5 = Pre-Victorian (≤1836). Home type categories are: Semi-detached; Terraced; Flat/maisonette; Detached; Bungalow; Other. Home tenure categories are: Own/part own; Rented private; Social housing; Live with family or friends; Other. Social grade categories are: AB (higher and intermediate managerial, administrative, professional occupations) =1; C1 (supervisory, clerical and junior managerial, administrative, professional occupations) =2; C2 (skilled manual occupations) =3; DE (semi-skilled and unskilled manual occupations, unemployed and lowest grade occupations) =4. Geographical areas are: Southern England, Middle England, Northern England, Northern Ireland, Scotland and Wales.

(a) ATTITUDE TOWARDS BADGERS

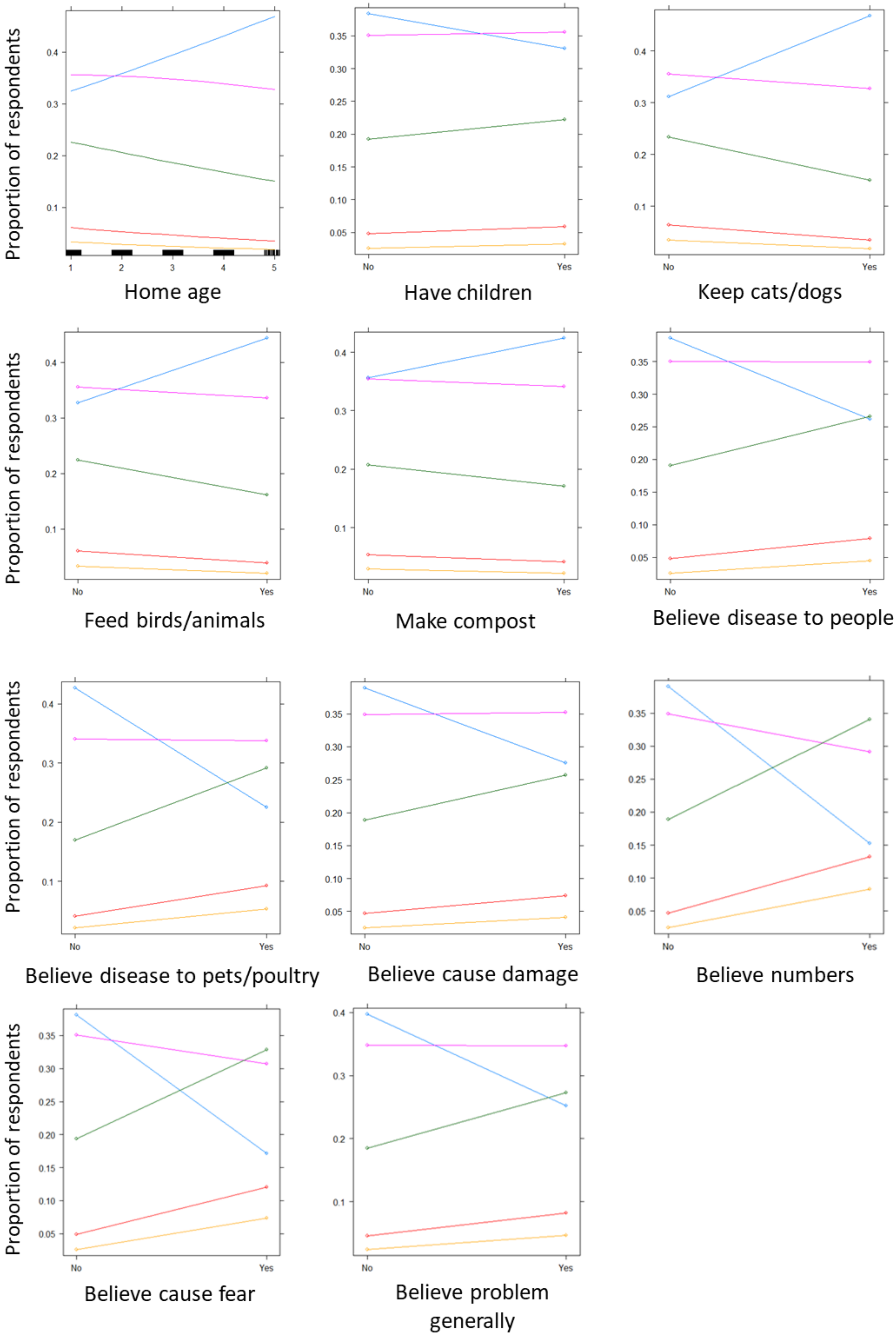

(b) ATTITUDE TOWARDS MOLES

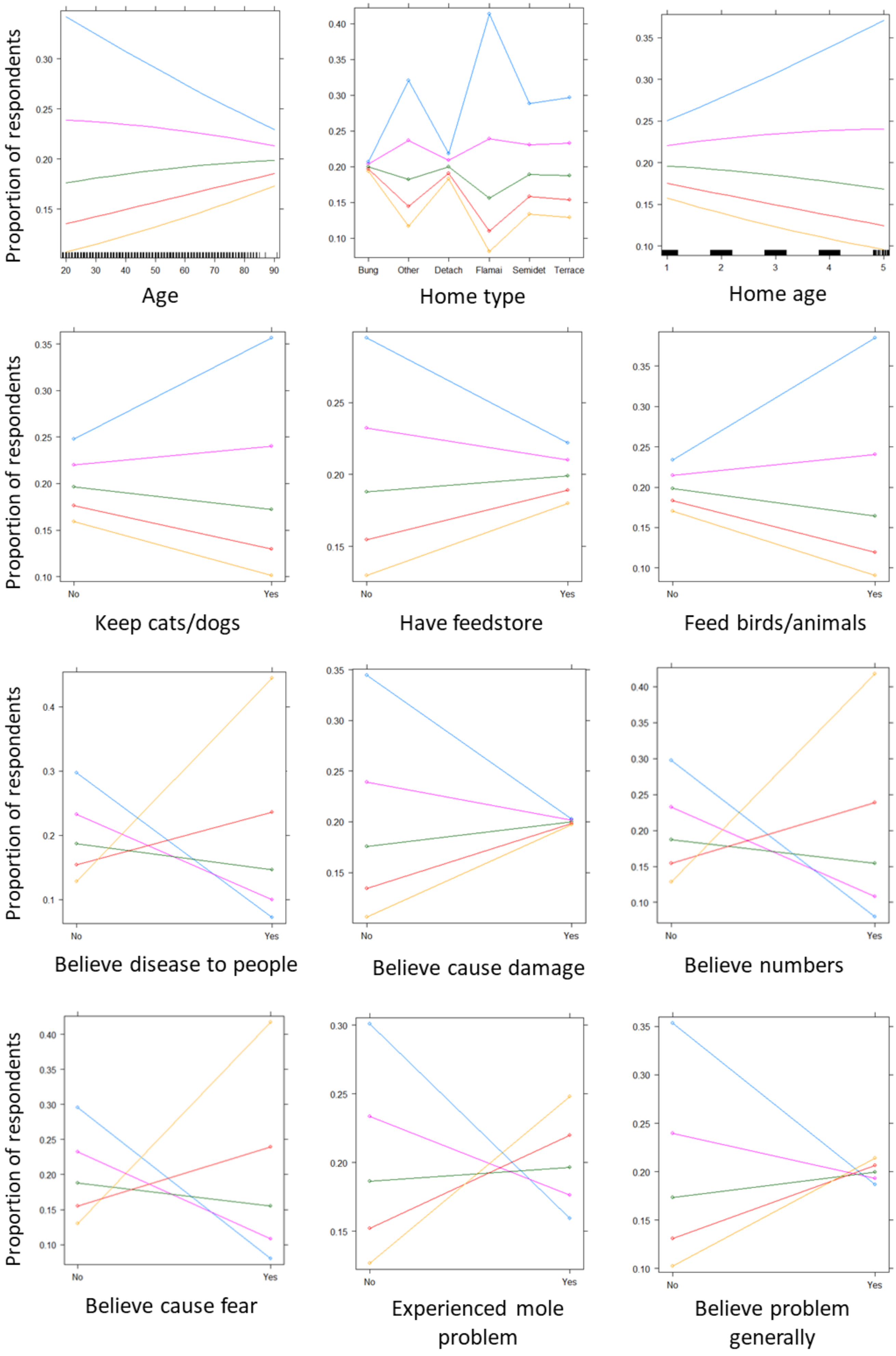

(c) ATTITUDE TOWARDS MICE

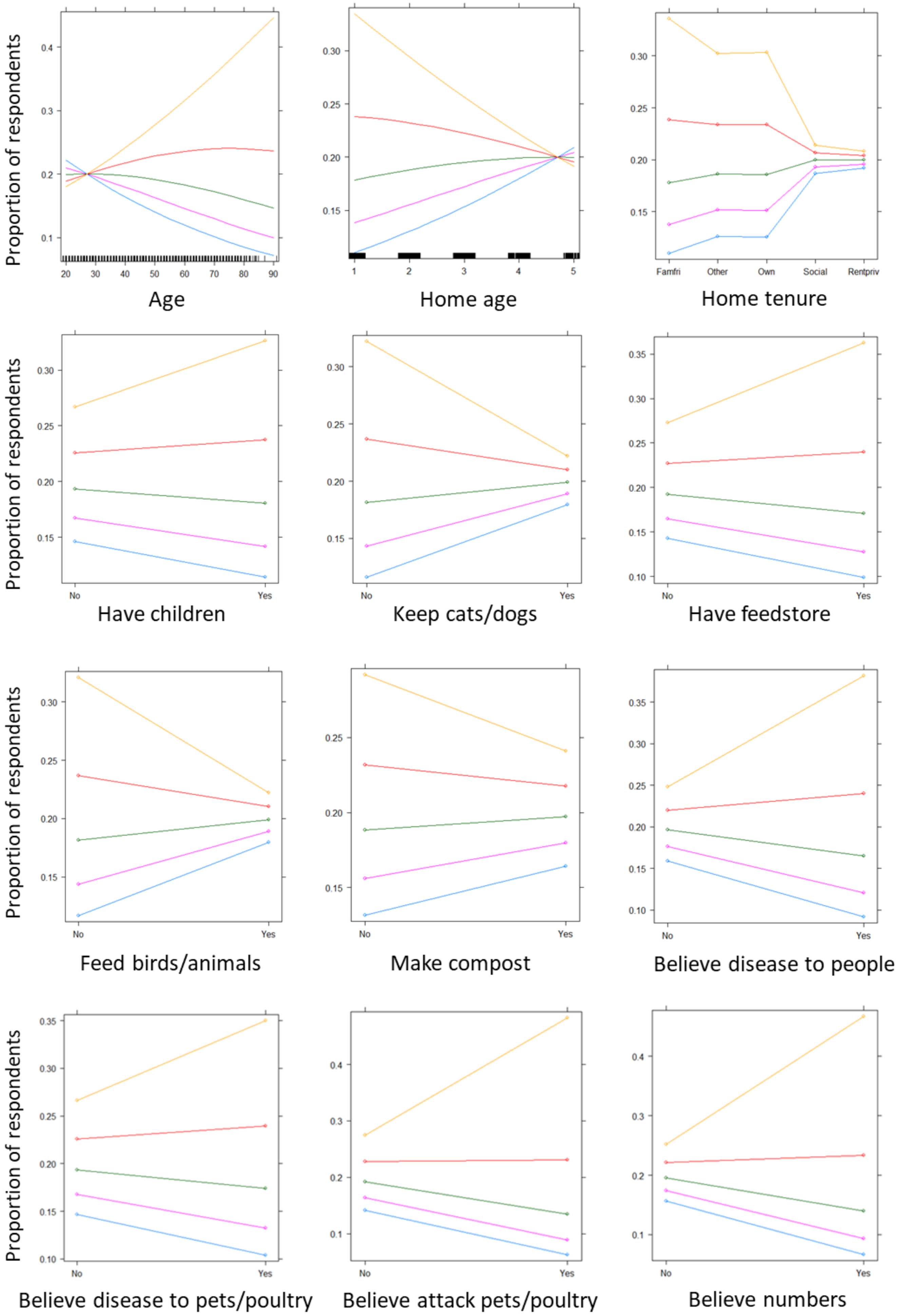

(c) ATTITUDE TOWARDS MICE (CONTINUED)

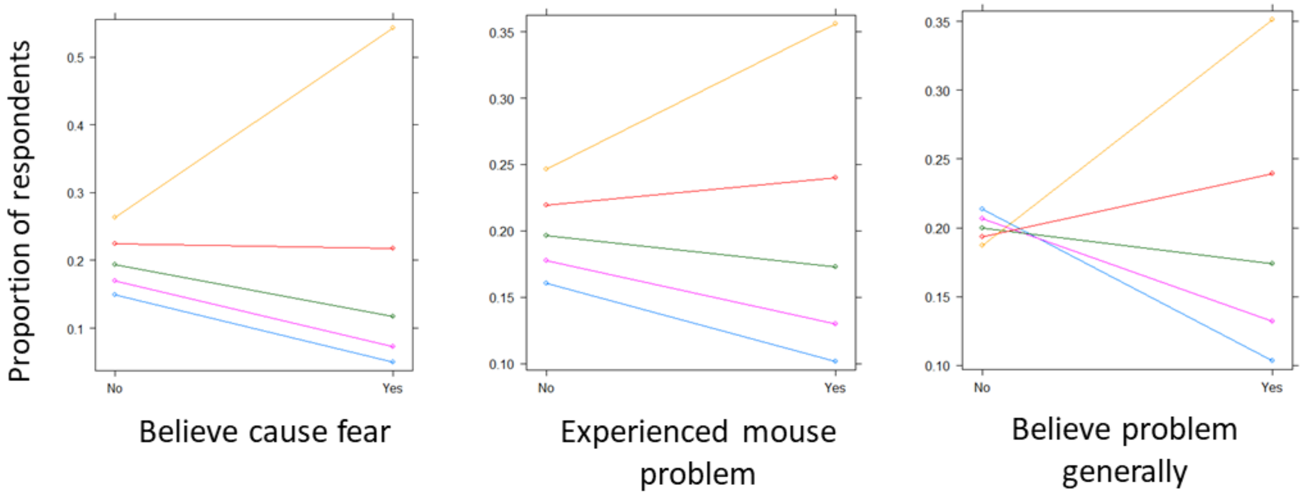

(d) ATTITUDE TOWARDS PIGEONS

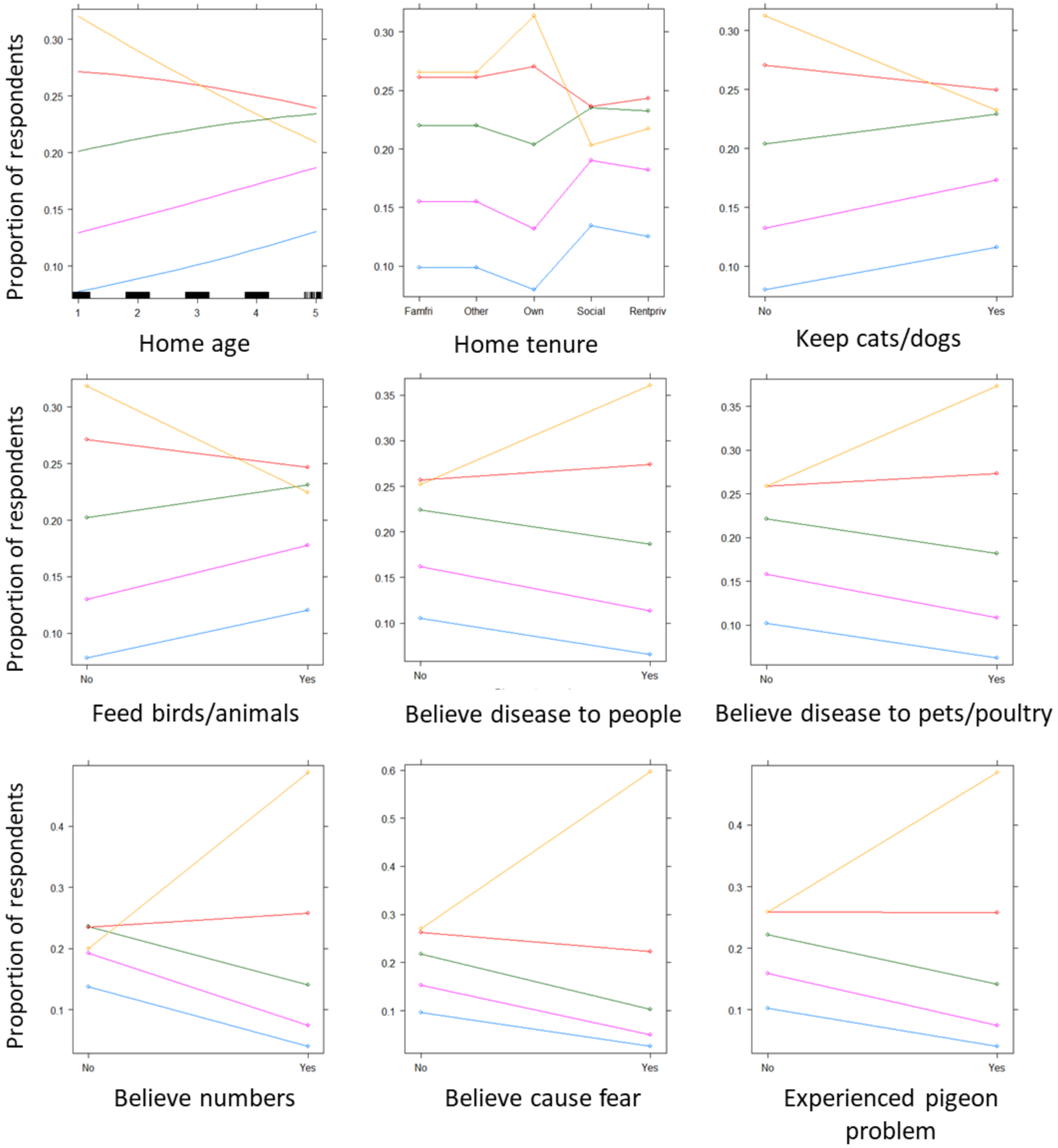

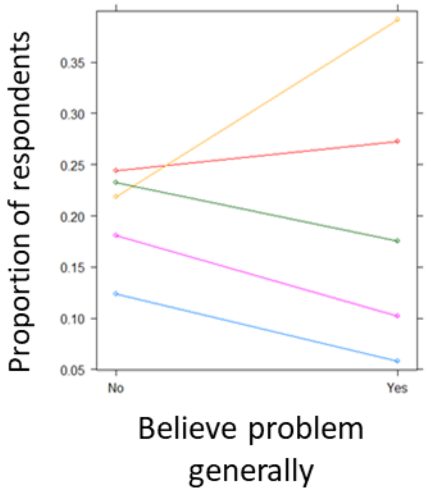

(e) ATTITUDE TOWARDS RABBITS

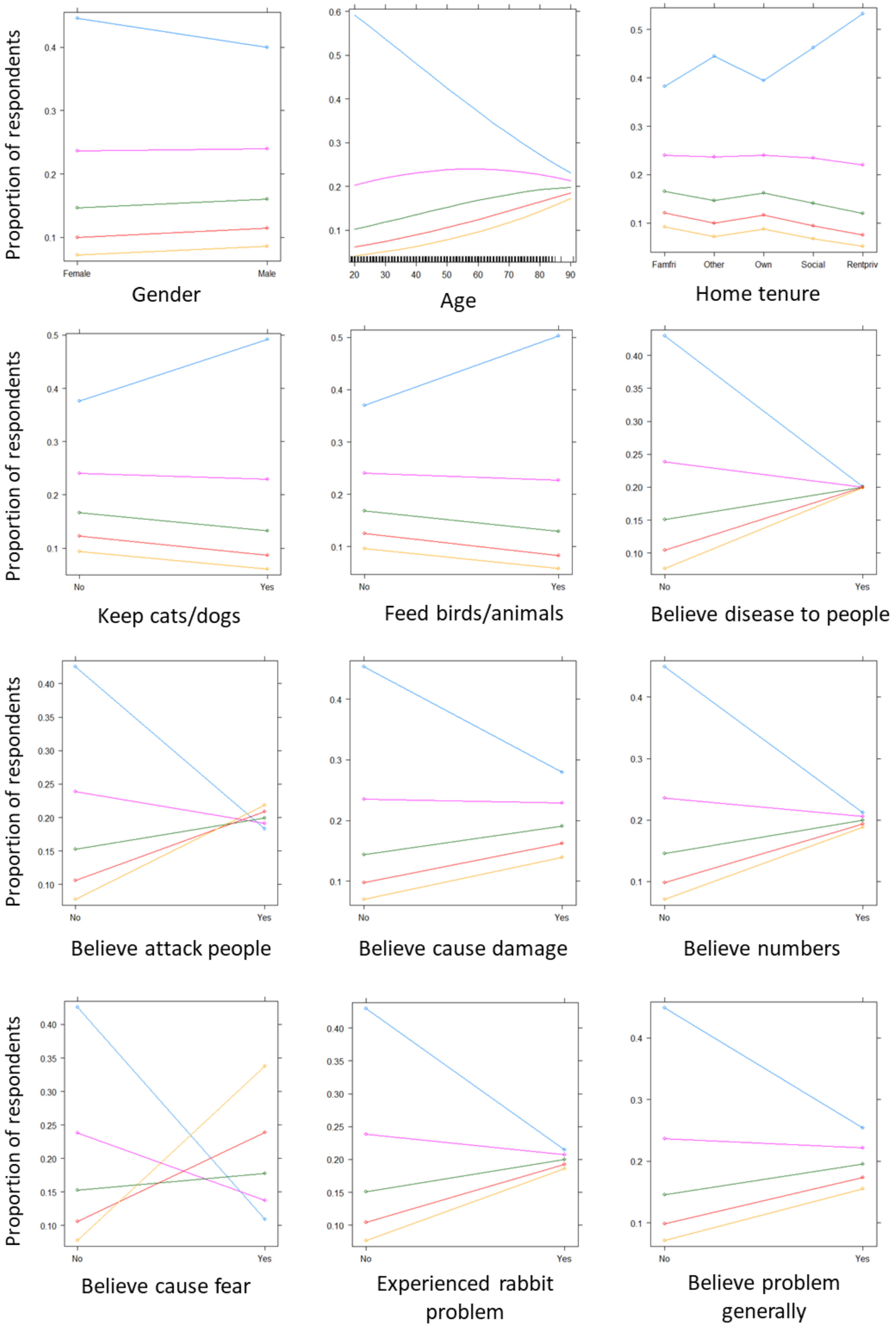

(f) ATTITUDE TOWARDS RATS

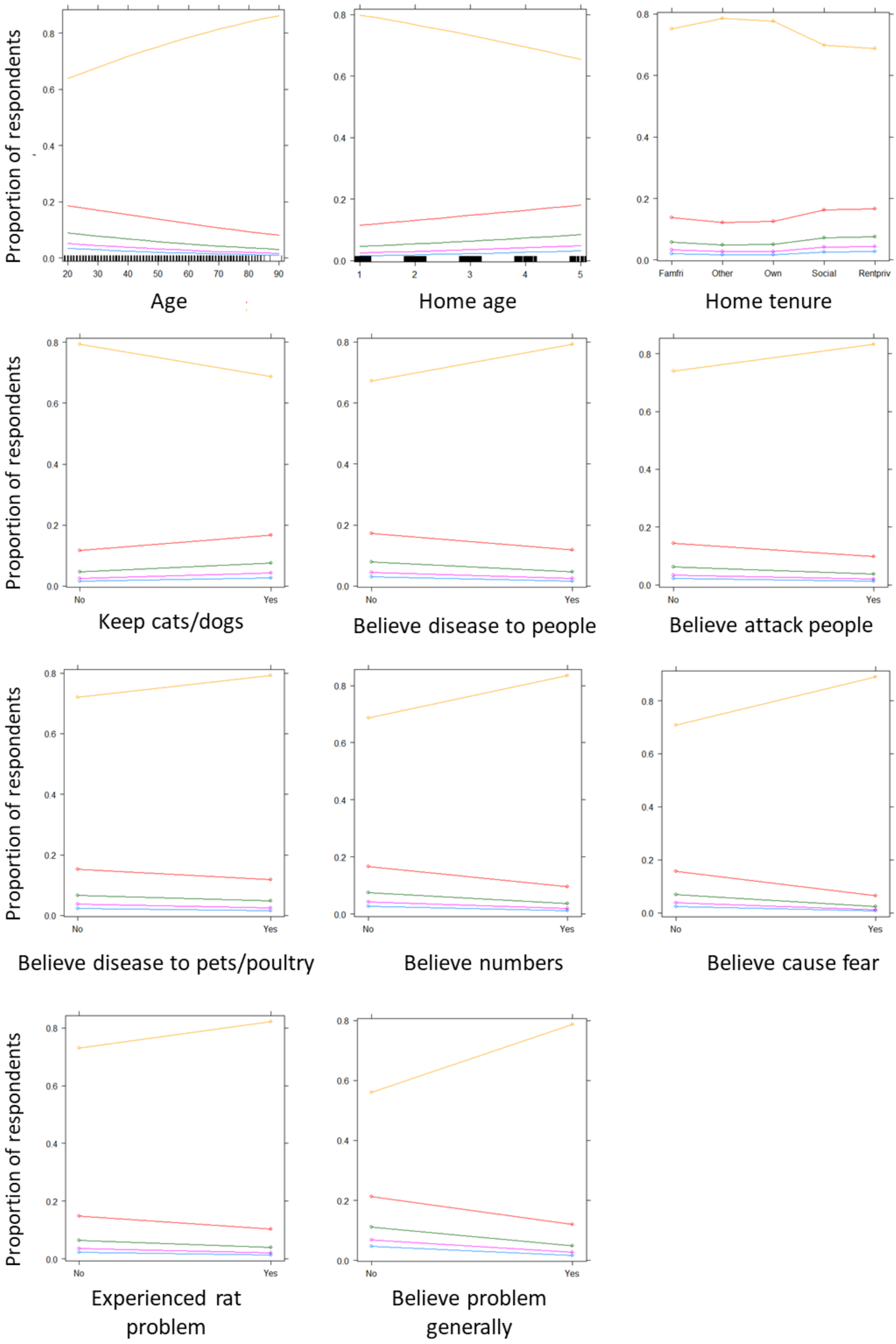

(g) ATTITUDE TOWARDS GULLS

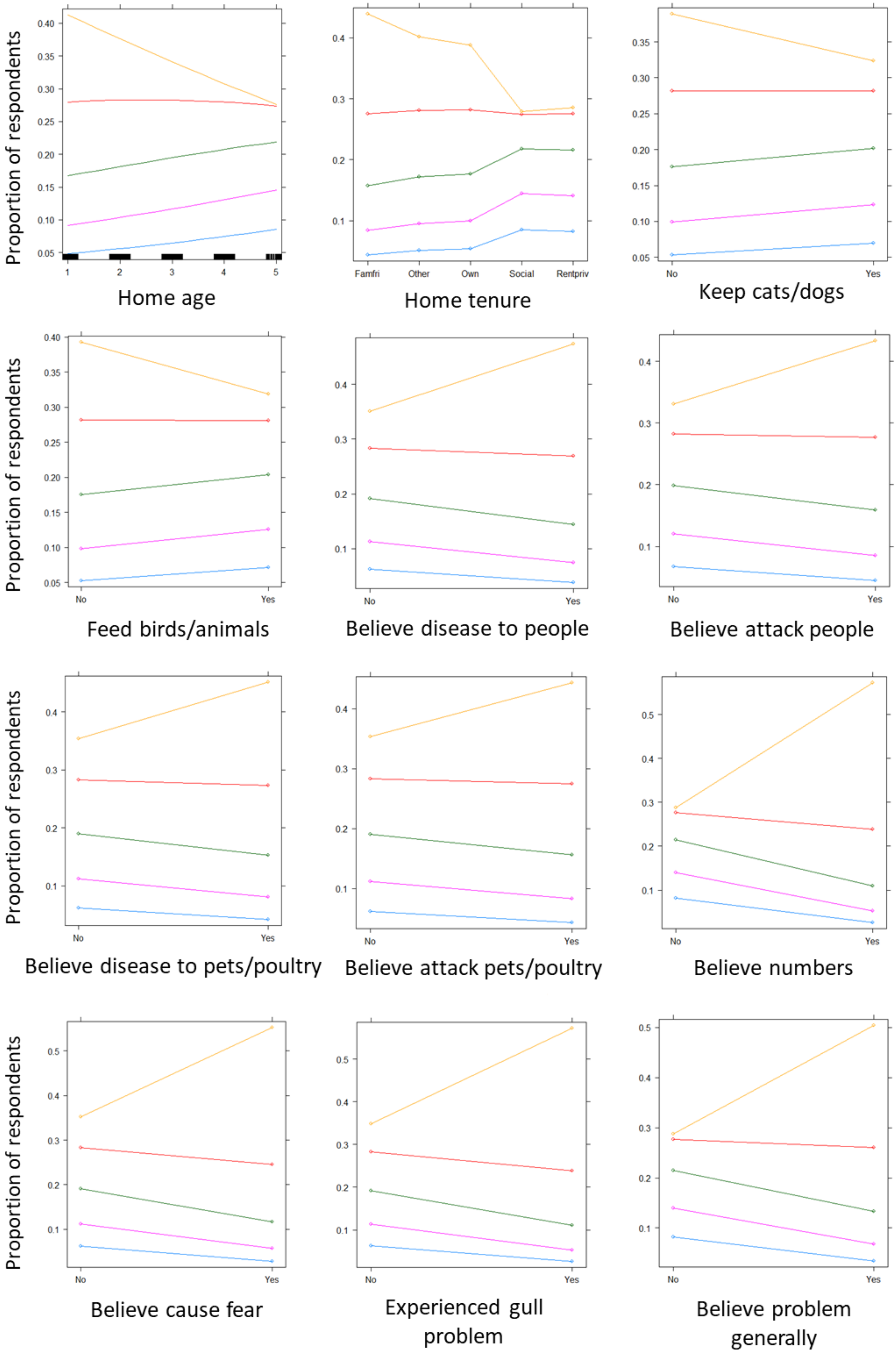

(h) ATTITUDE TOWARDS WASPS

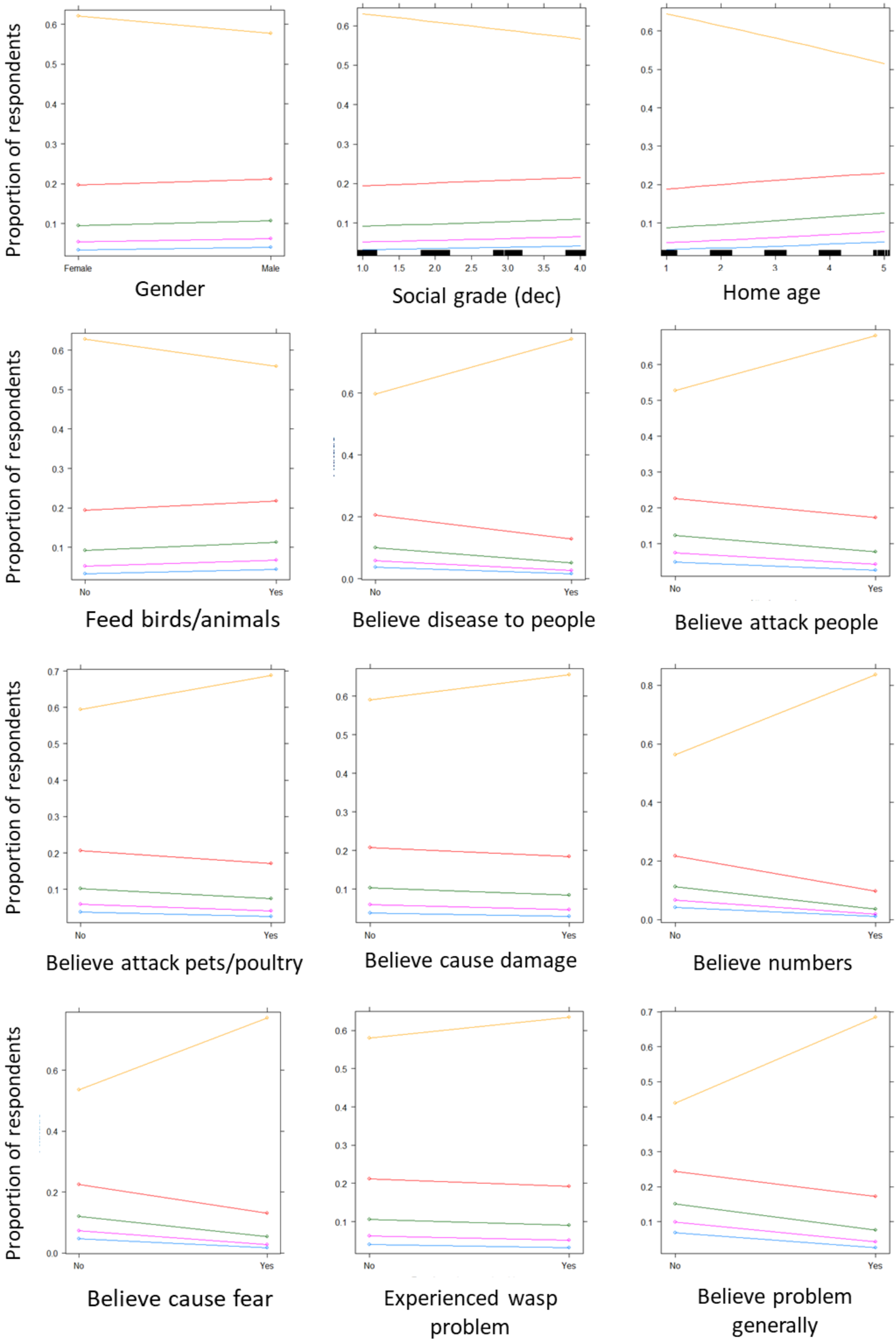

Figure S10. Drivers of respondents' experience of problems with each species (marginal effects in logistic regression models): (a) badgers; (b) foxes; (c) moles; (d) mice; (e) pigeons; (f) rabbits; (g) rats; (h) gulls. Confidence Intervals are shown in grey. Home age categories are: 1 = 21st Century (≥2001), 2 = Post World War II (1946-2000), 3 = Pre-World War II (1902-1945), 4 = Victorian (1837-1901), 5 = Pre-Victorian (≤1836). Home type categories are: Semi-detached; Terraced; Flat/maisonette; Detached; Bungalow; Other. Home tenure categories are: Own/part own; Rented private; Social housing; Live with family or friends; Other. Social grade categories are: AB (higher and intermediate managerial, administrative, professional occupations) =1; C1 (supervisory, clerical and junior managerial, administrative, professional occupations) =2; C2 (skilled manual occupations) =3; DE (semi-skilled and unskilled manual occupations, unemployed and lowest grade occupations) =4. Geographical areas are: Southern England, Middle England, Northern England, Northern Ireland, Scotland and Wales.

(a) EXPERIENCE OF BADGER PROBLEMS

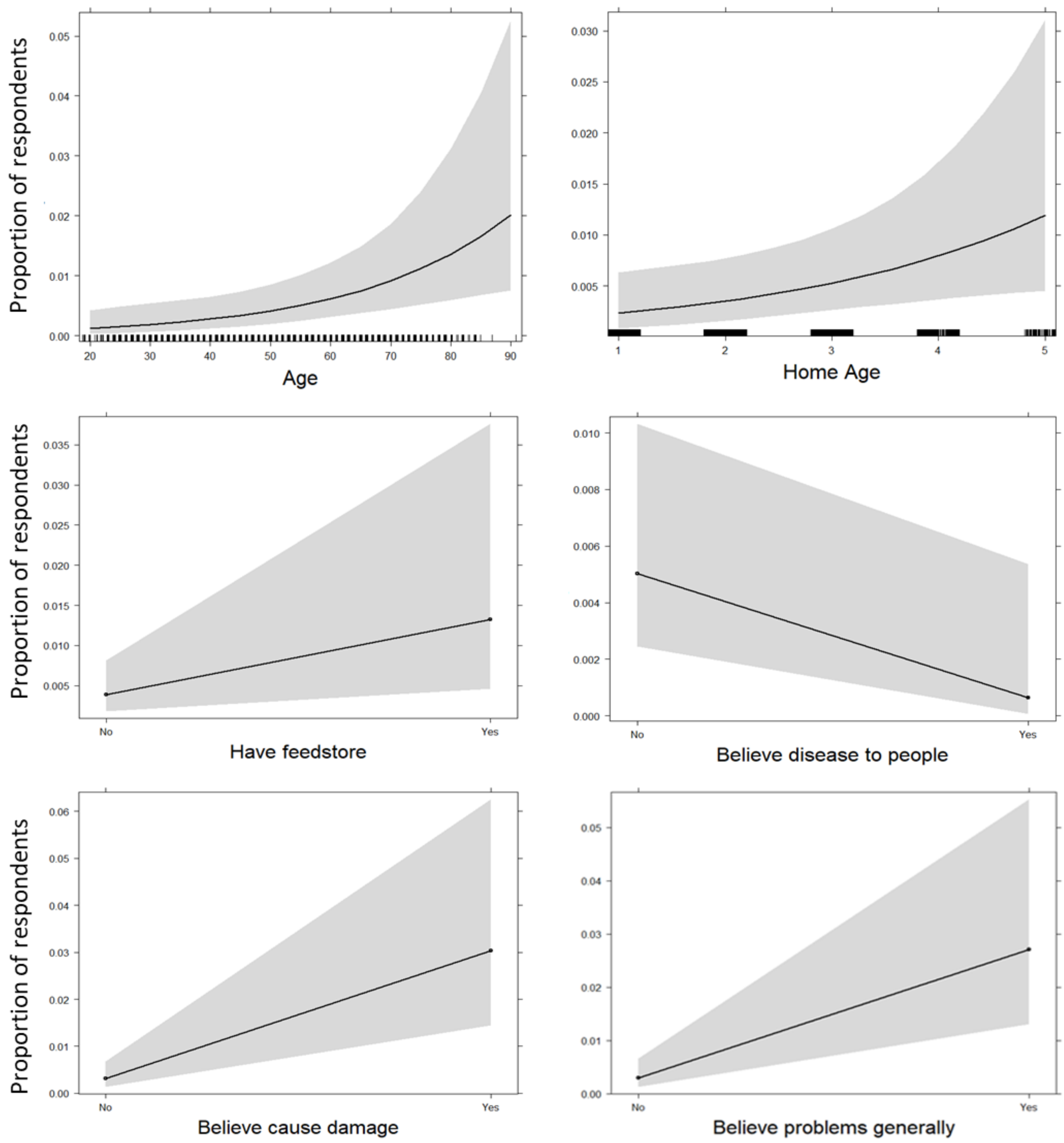

(b) EXPERIENCE OF FOX PROBLEMS

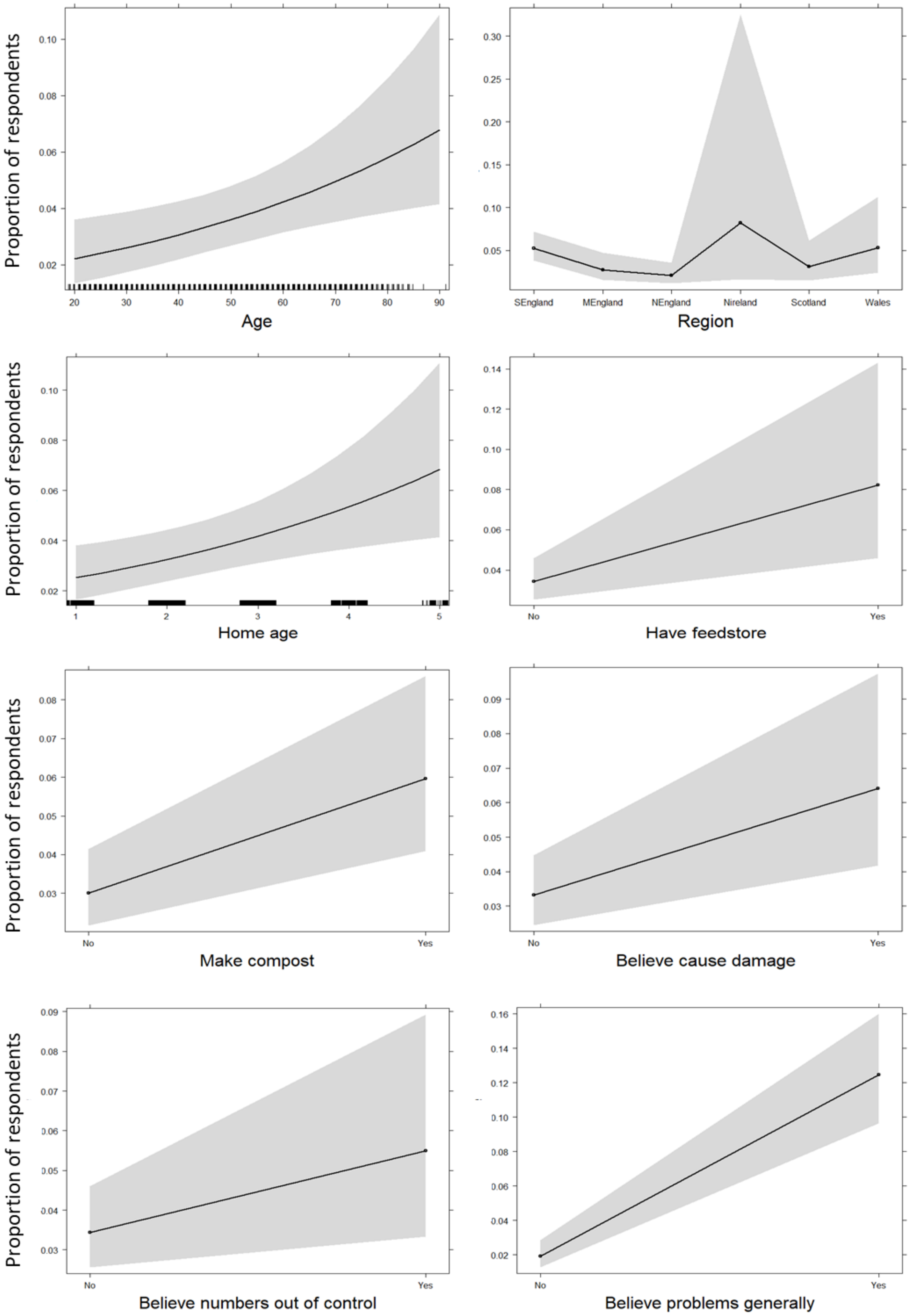

(b) EXPERIENCE OF FOX PROBLEMS (CONTINUED)

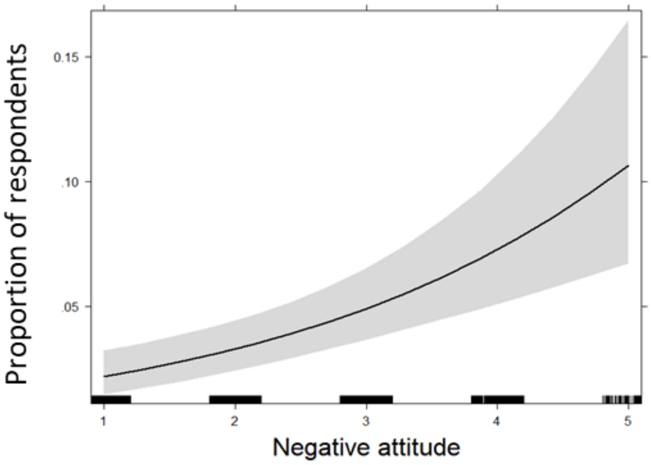

(c) EXPERIENCE OF MOLE PROBLEMS

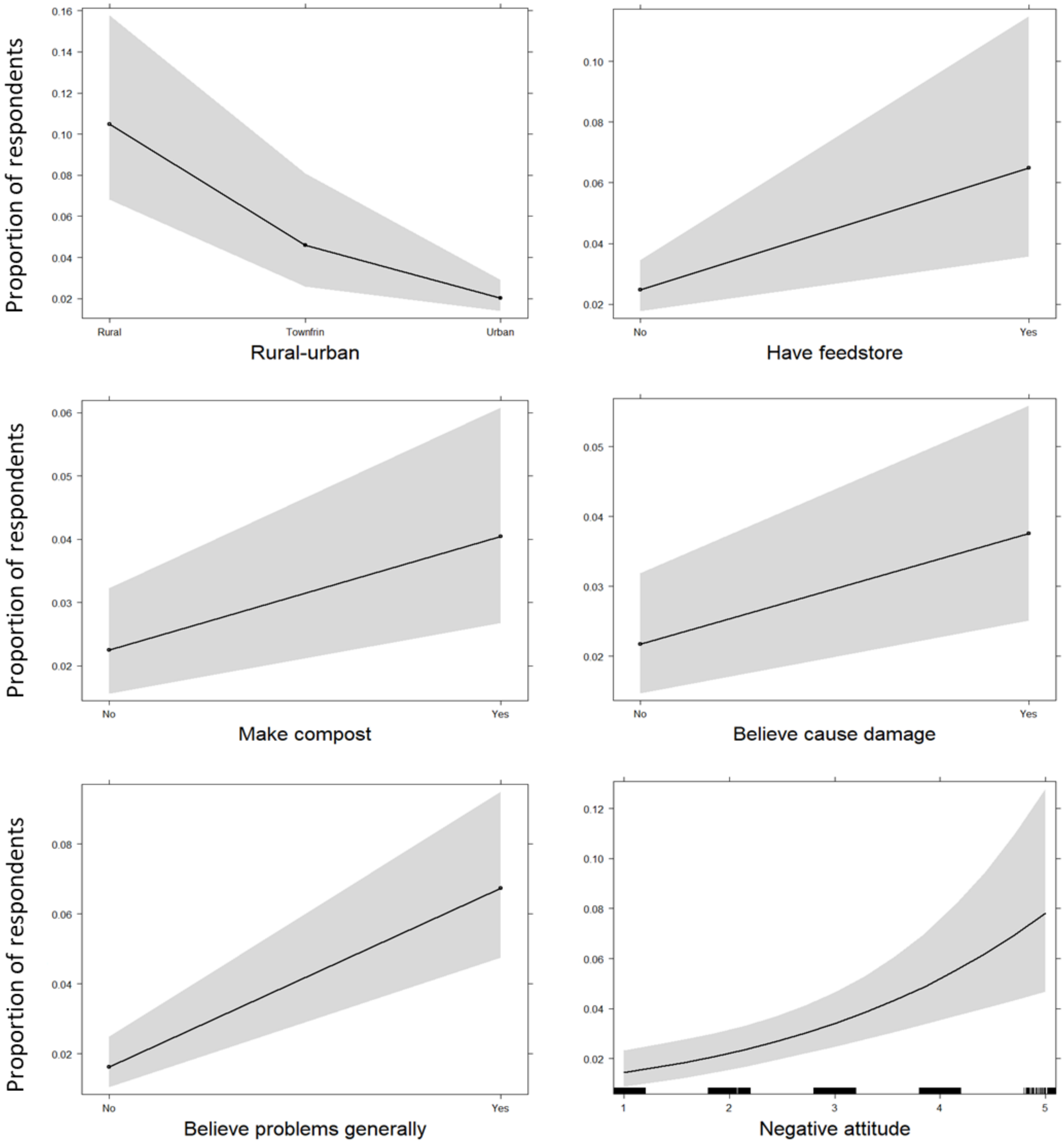

(d) EXPERIENCE OF MOUSE PROBLEMS

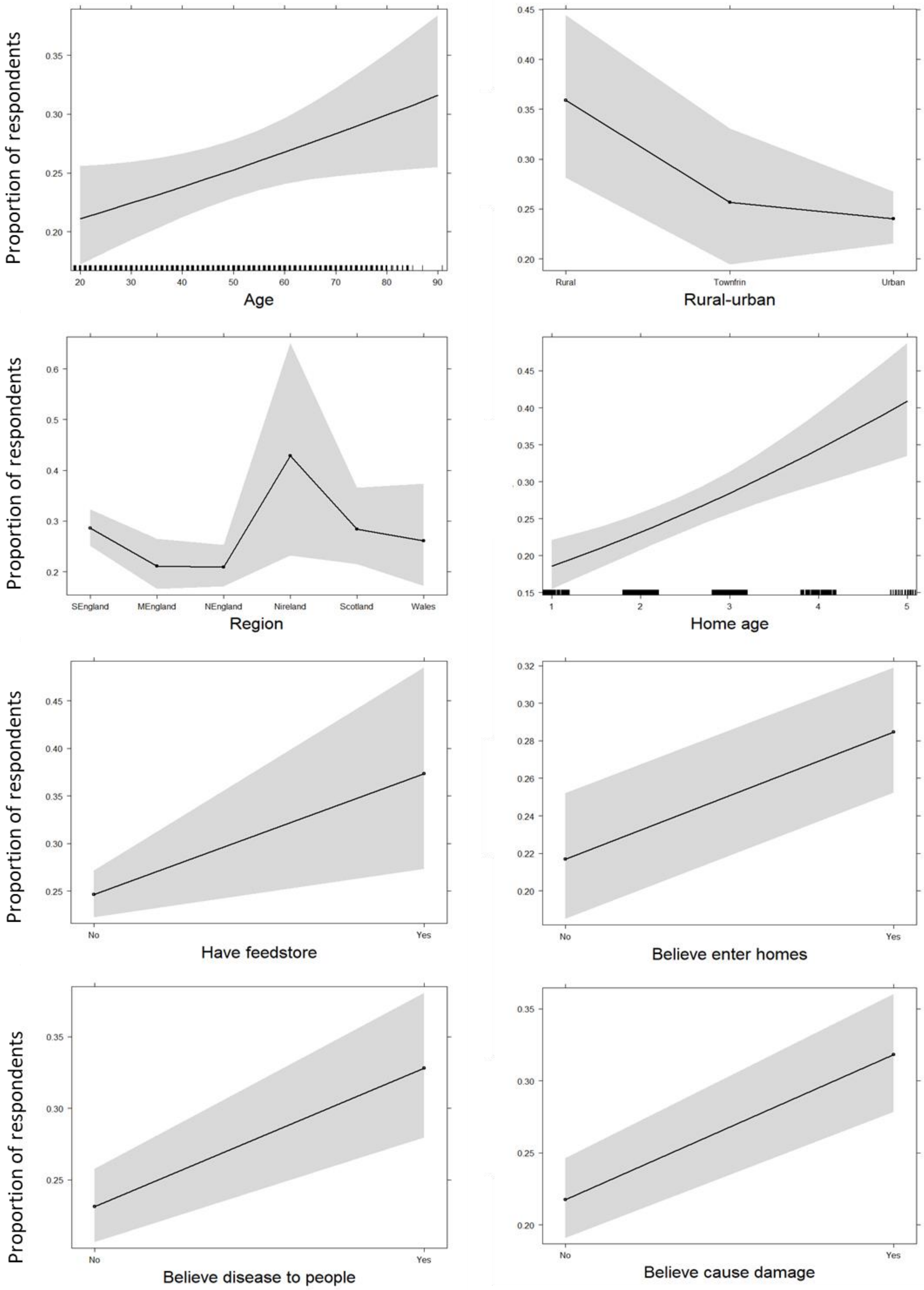

(d) EXPERIENCE OF MOUSE PROBLEMS (CONTINUED)

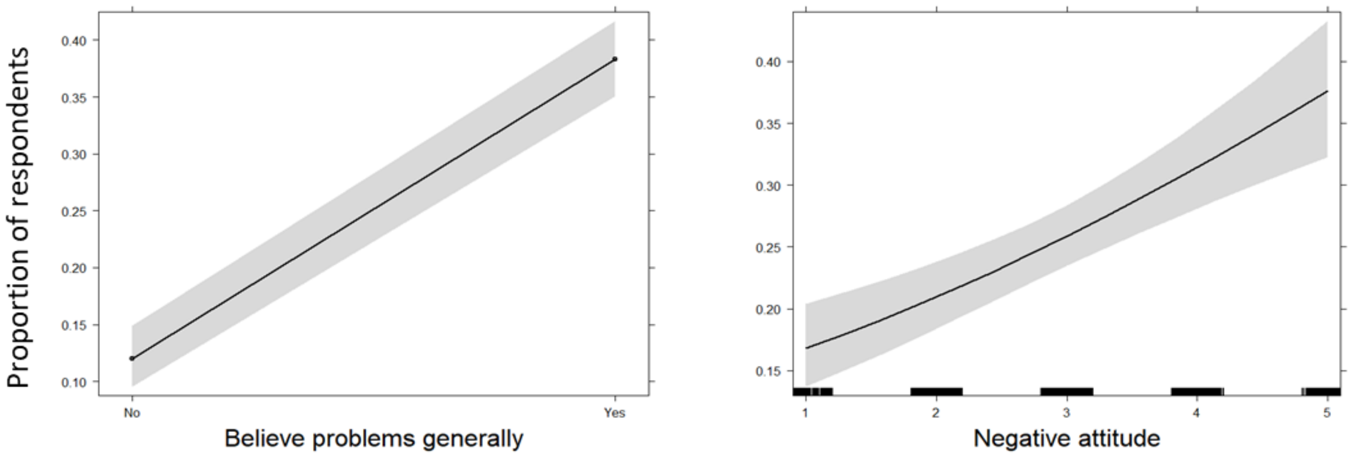

(e) EXPERIENCE OF PIGEON PROBLEMS

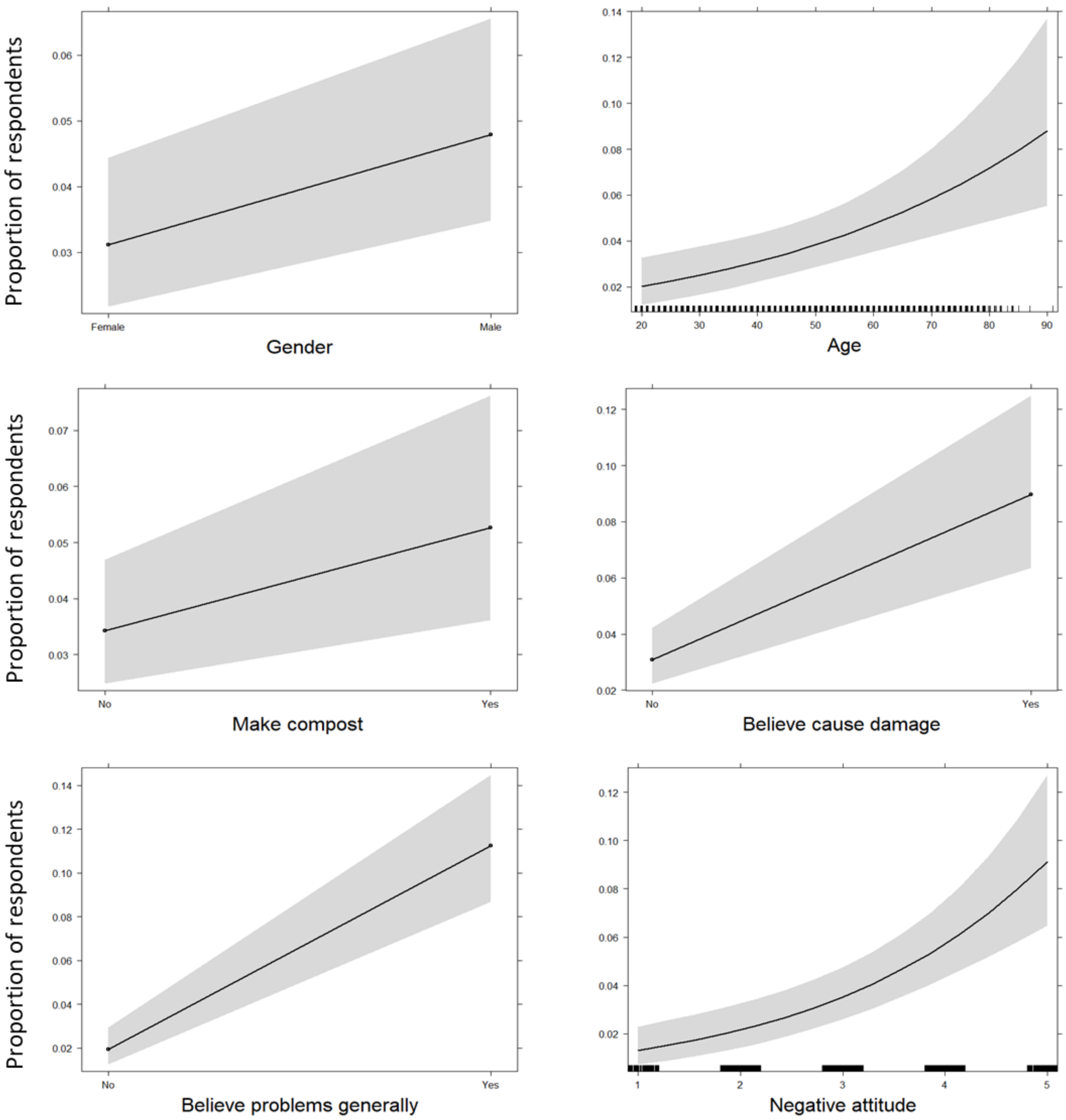

(f) EXPERIENCE OF RABBIT PROBLEMS

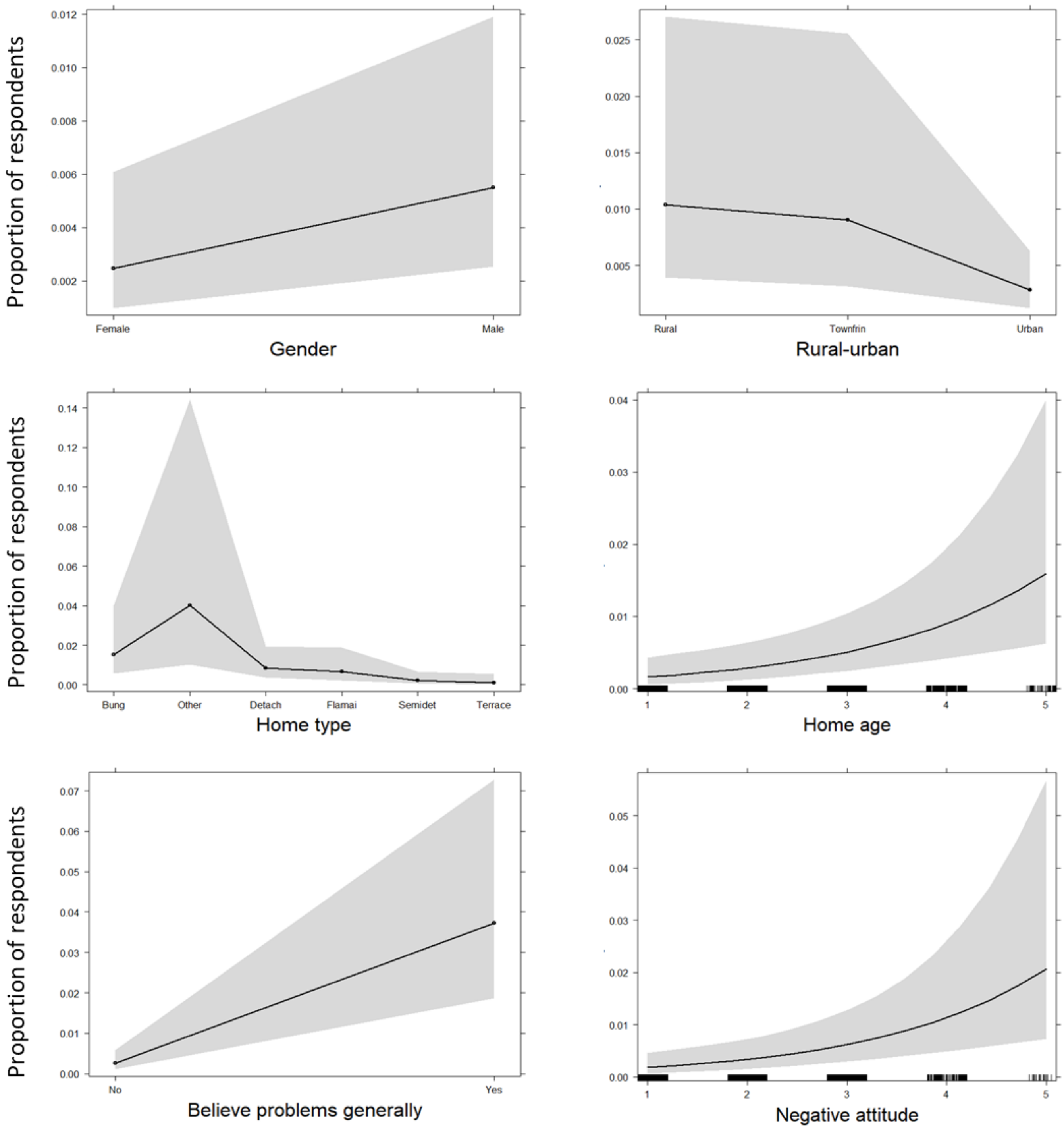

(g) EXPERIENCE OF RAT PROBLEMS

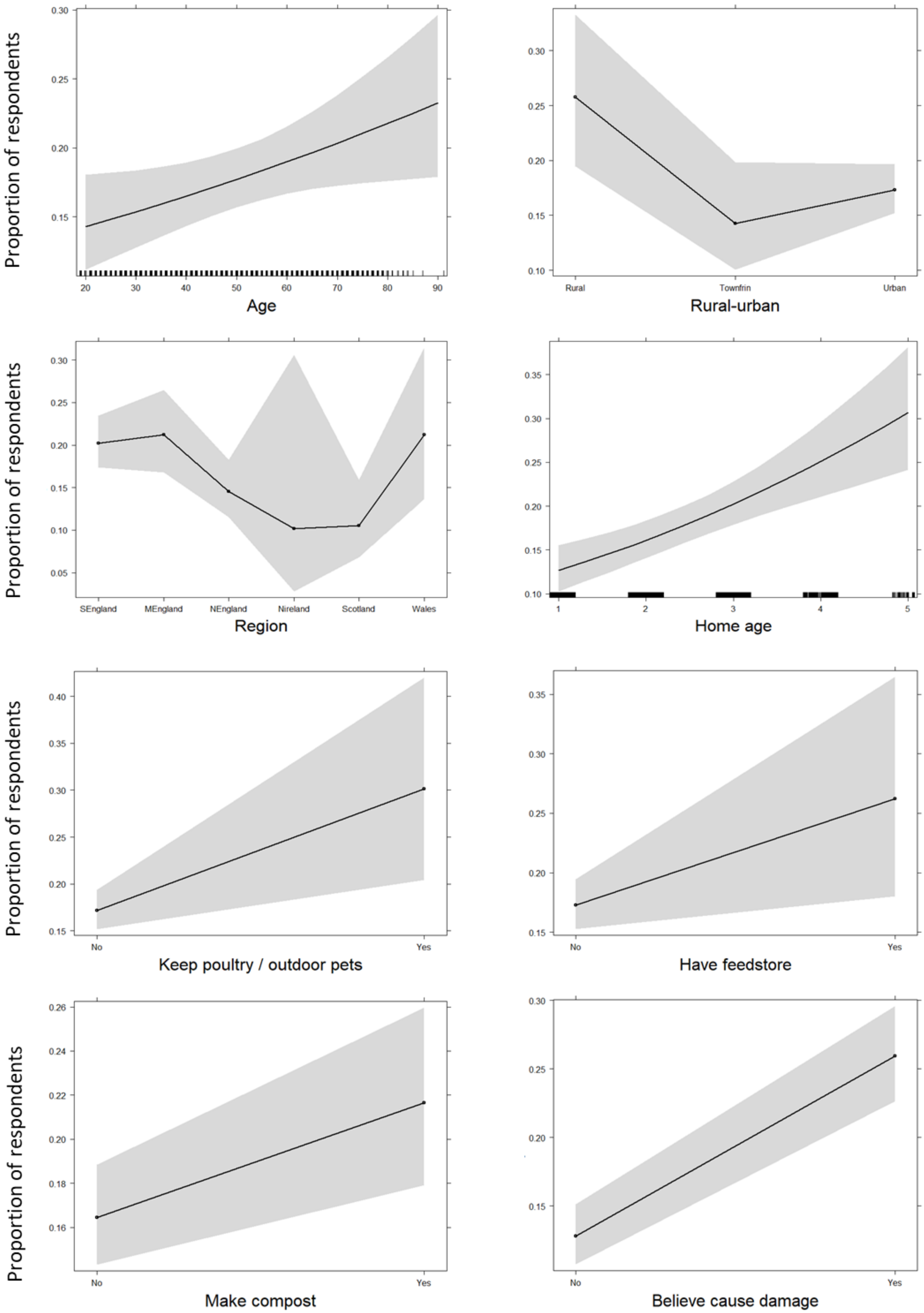

(g) EXPERIENCE OF RAT PROBLEMS (CONTINUED)

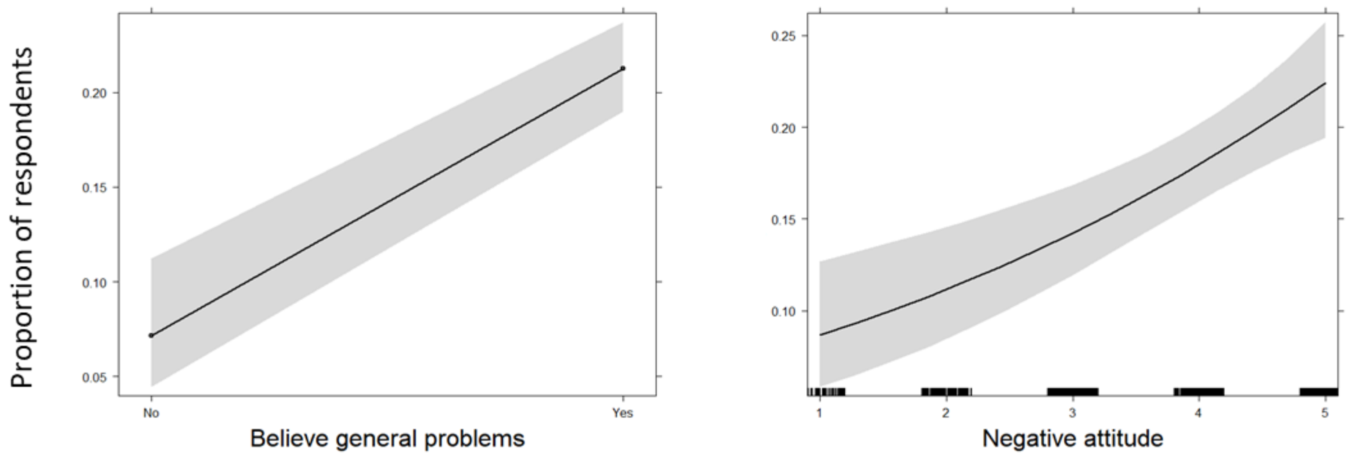

(h) EXPERIENCE OF GULL PROBLEMS

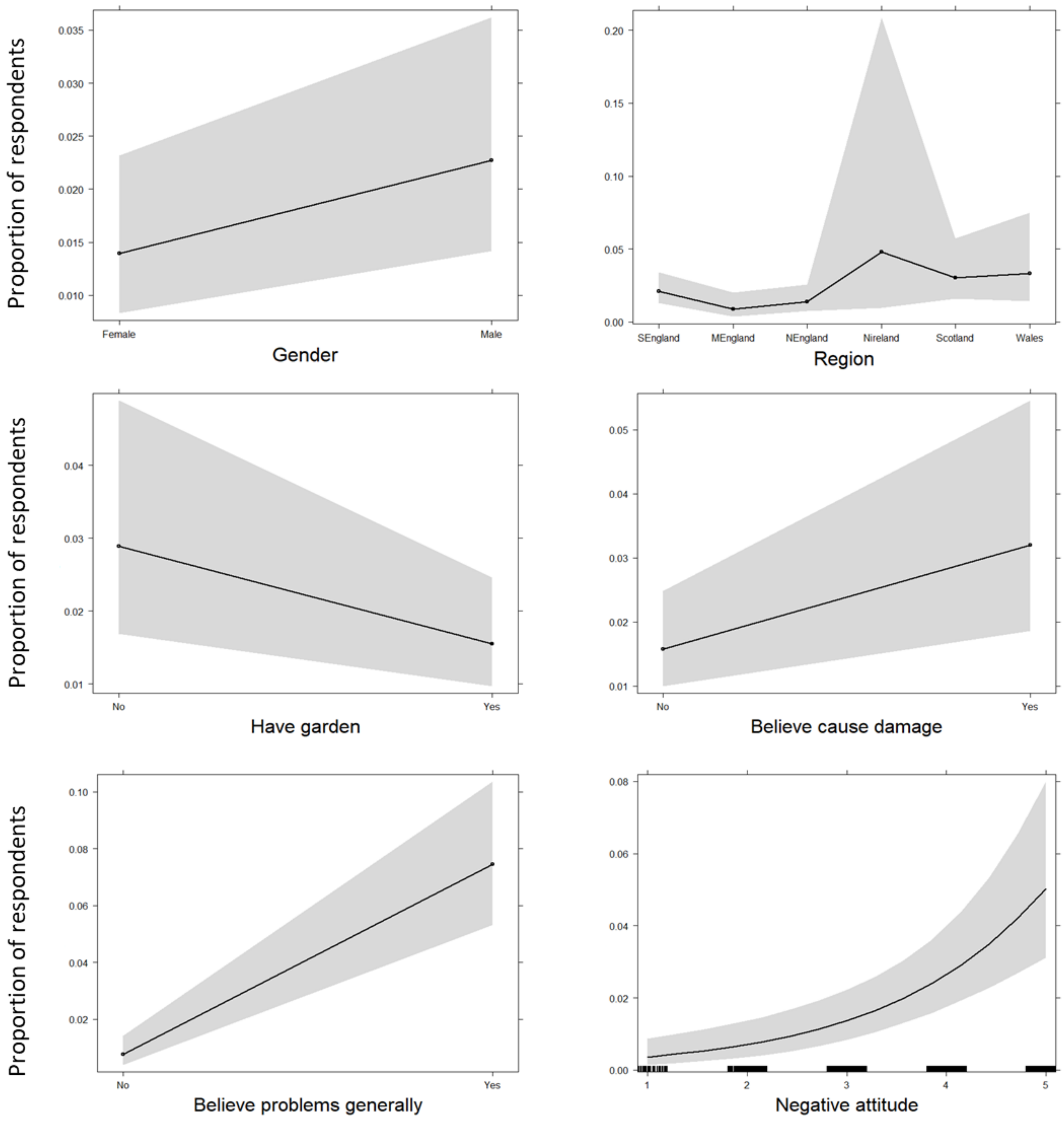

Figure S11. Drivers of respondents' beliefs that each species causes problems generally (marginal effects in logistic regression models): (a) badgers; (b) foxes; (c) moles; (d) mice; (e) pigeons; (f) rabbits; (g) rats; (h) gulls; (i) squirrels; (j) wasps. Confidence Intervals are shown in grey. 'Believe numbers' = Believe numbers are out of control. Home age categories are: 1 = 21st Century (≥2001), 2 = Post World War II (1946-2000), 3 = Pre-World War II (1902-1945), 4 = Victorian (1837-1901), 5 = Pre-Victorian (≤1836). Home type categories are: Semi-detached; Terraced; Flat/maisonette; Detached; Bungalow; Other. Home tenure categories are: Own/part own; Rented private; Social housing; Live with family or friends; Other. Social grade categories are: AB (higher and intermediate managerial, administrative, professional occupations) =1; C1 (supervisory, clerical and junior managerial, administrative, professional occupations) =2; C2 (skilled manual occupations) =3; DE (semi-skilled and unskilled manual occupations, unemployed and lowest grade occupations) =4. Geographical areas are: Southern England, Middle England, Northern England, Northern Ireland, Scotland and Wales.

(a) BELIEVE BADGERS CAUSE PROBLEMS GENERALLY

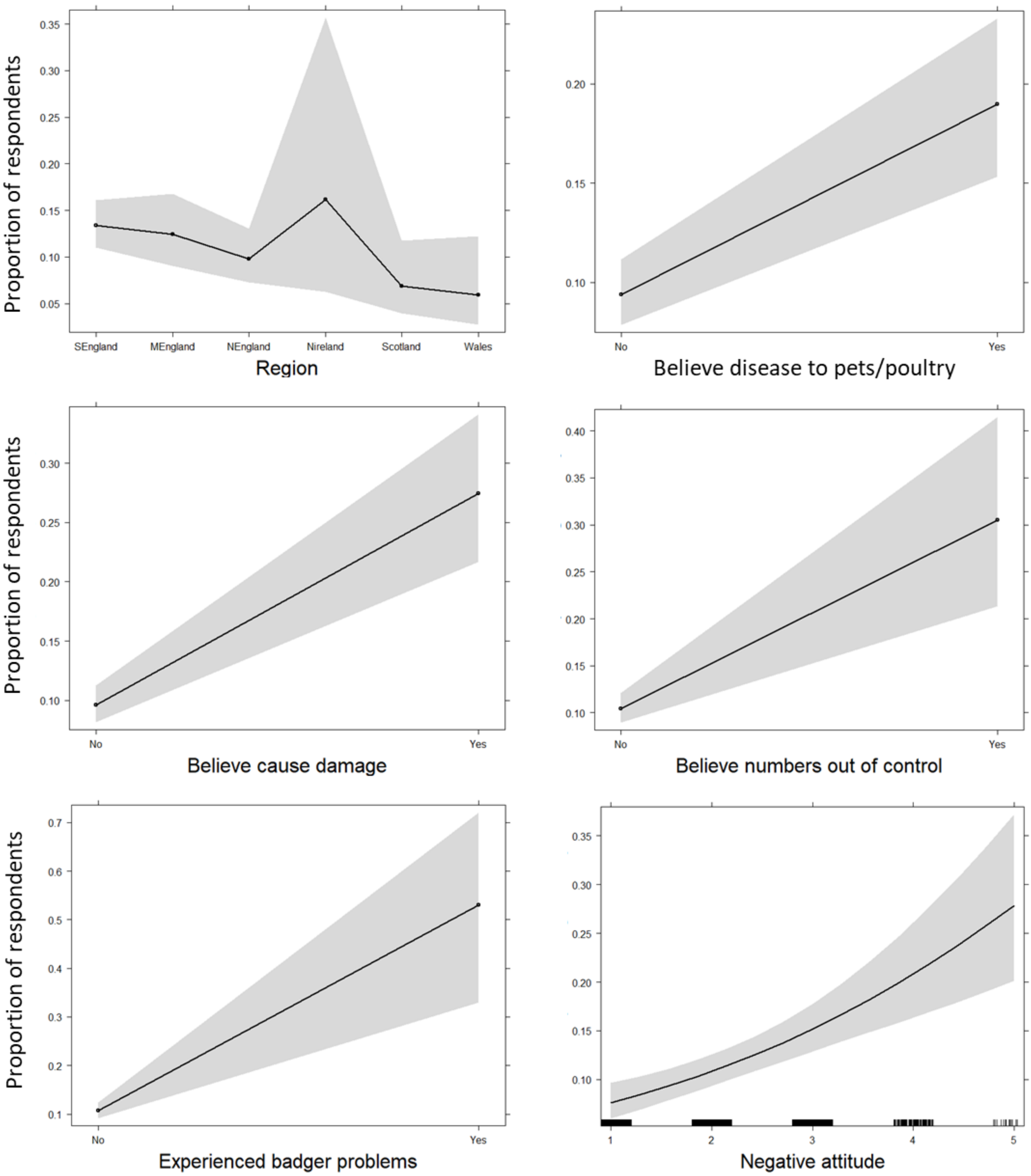

(b) BELIEVE FOXES CAUSE PROBLEMS GENERALLY

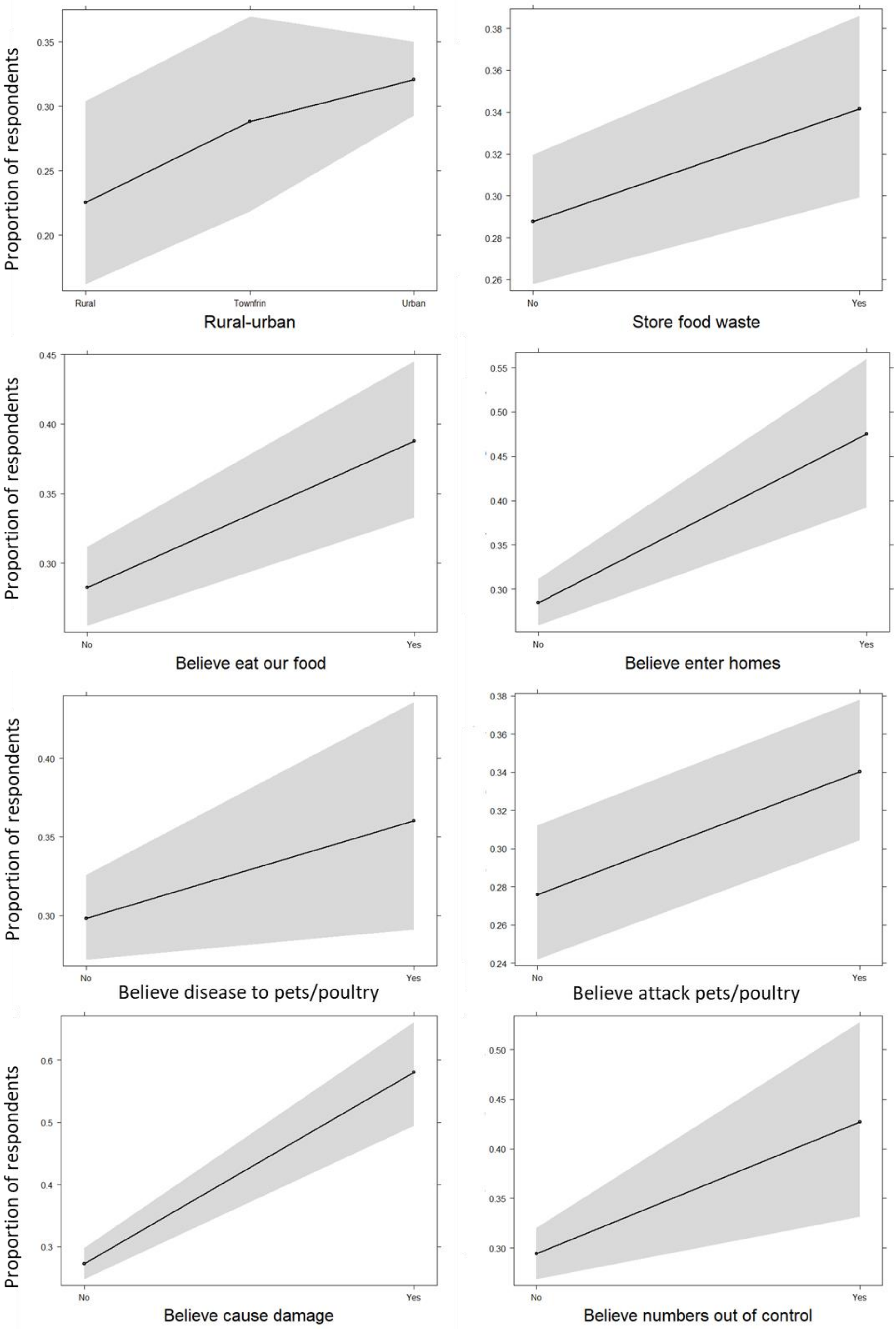

(b) BELIEVE FOXES CAUSE PROBLEMS GENERALLY (CONTINUED)

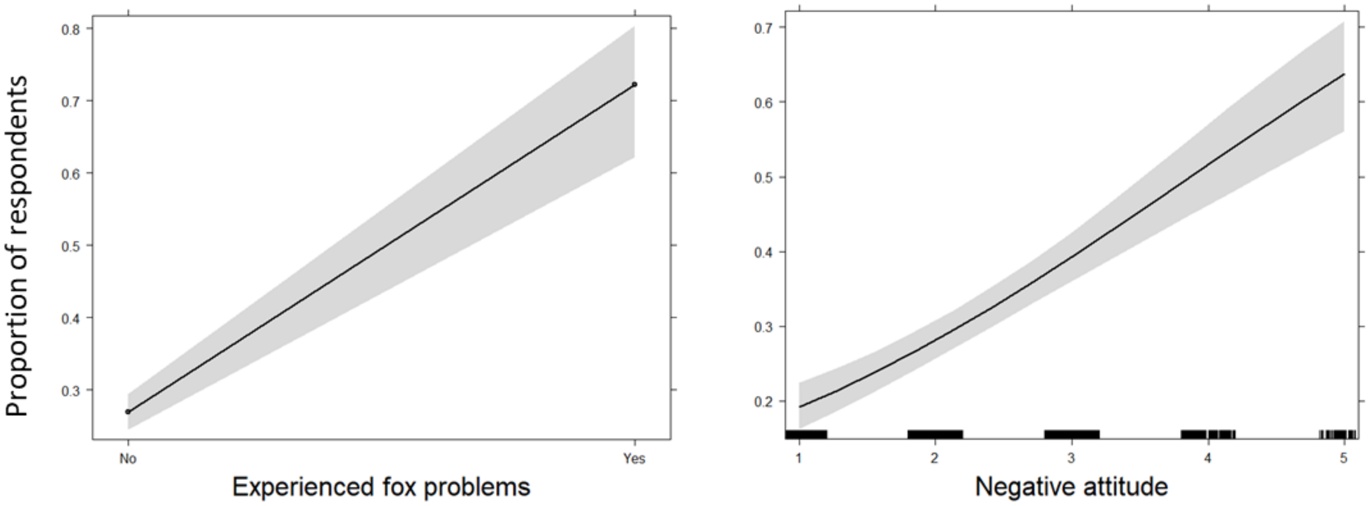

(c) BELIEVE MOLES CAUSE PROBLEMS GENERALLY

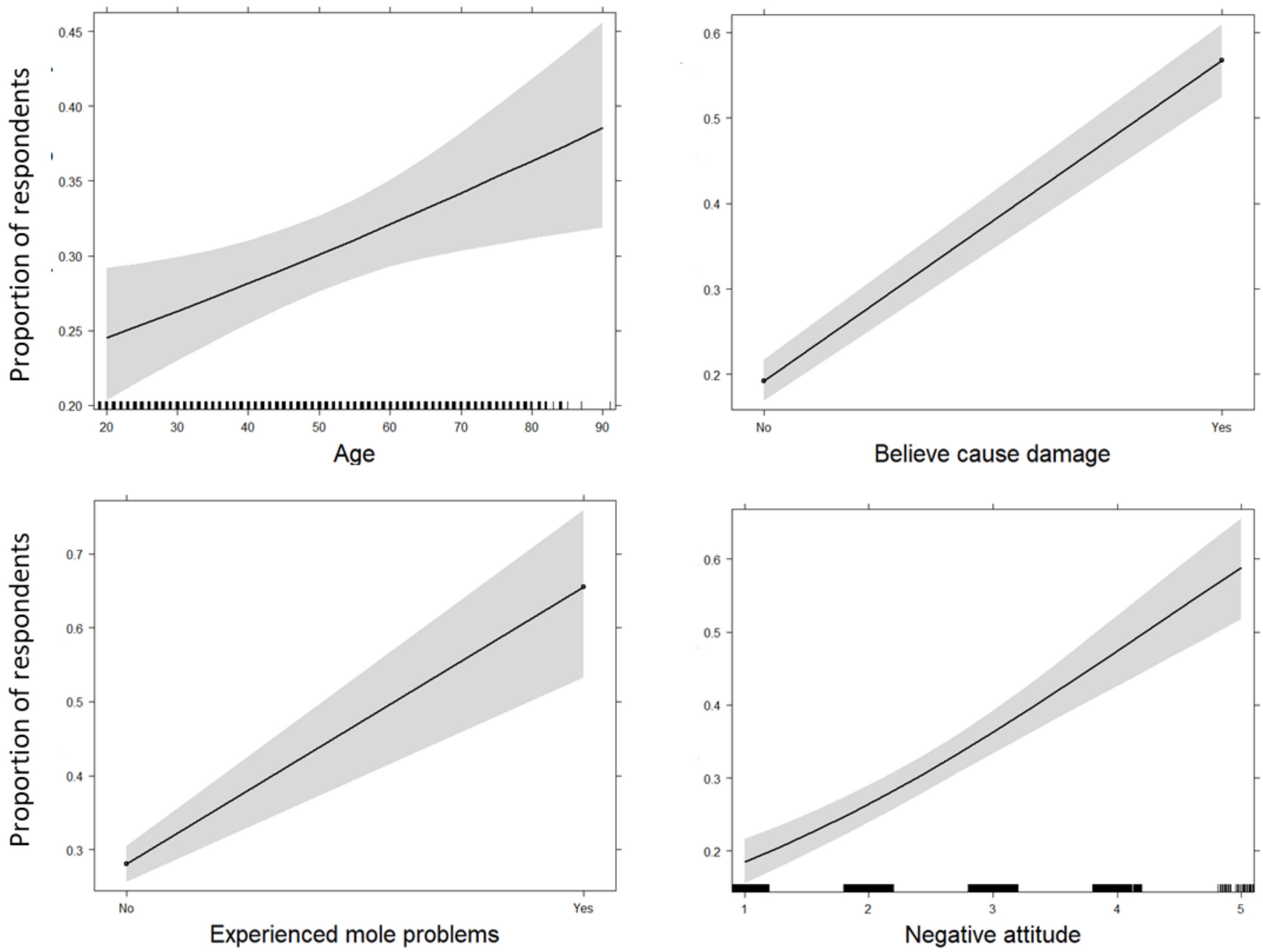

(d) BELIEVE MICE CAUSE PROBLEMS GENERALLY

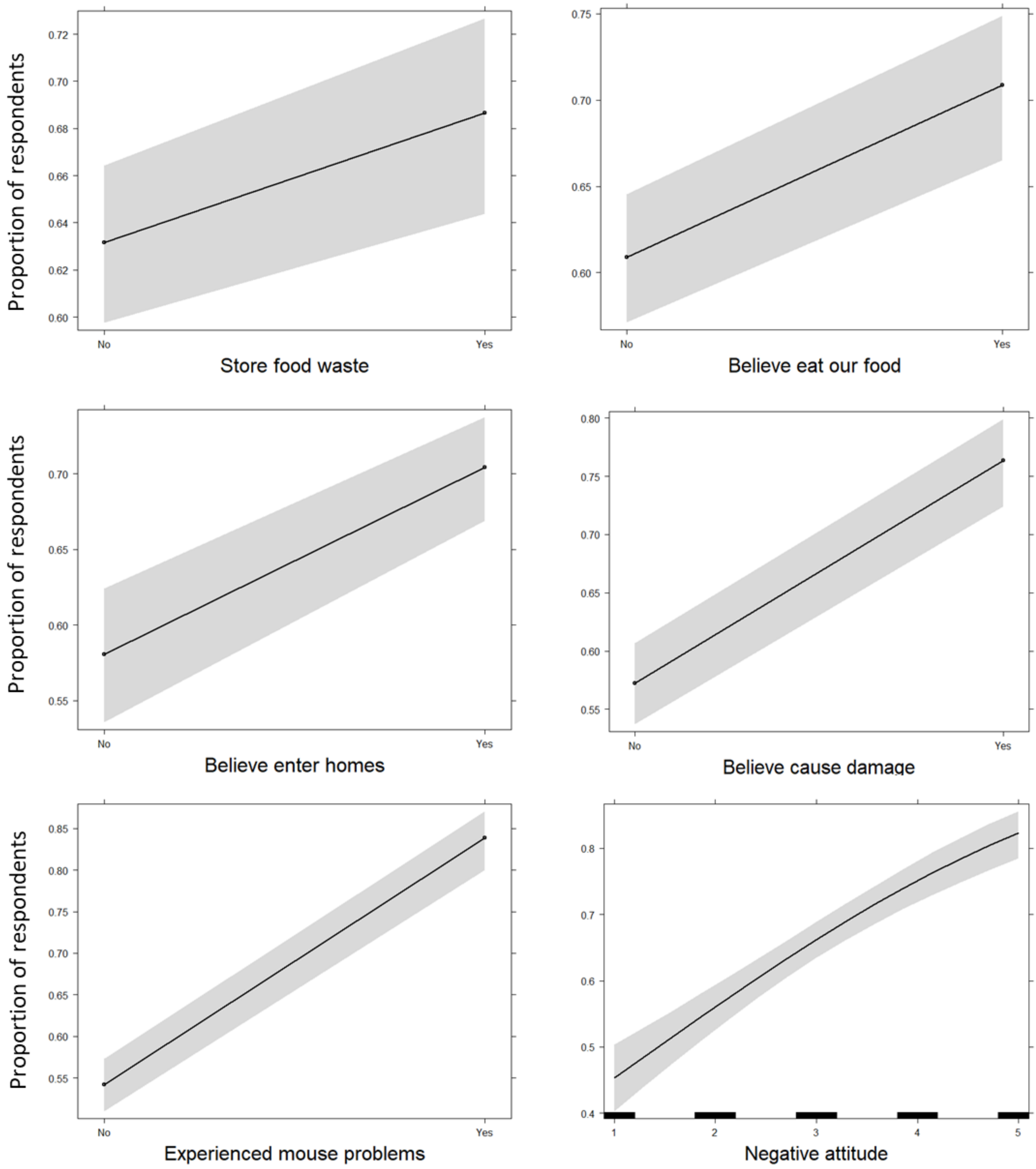

(e) BELIEVE PIGEONS CAUSE PROBLEMS GENERALLY

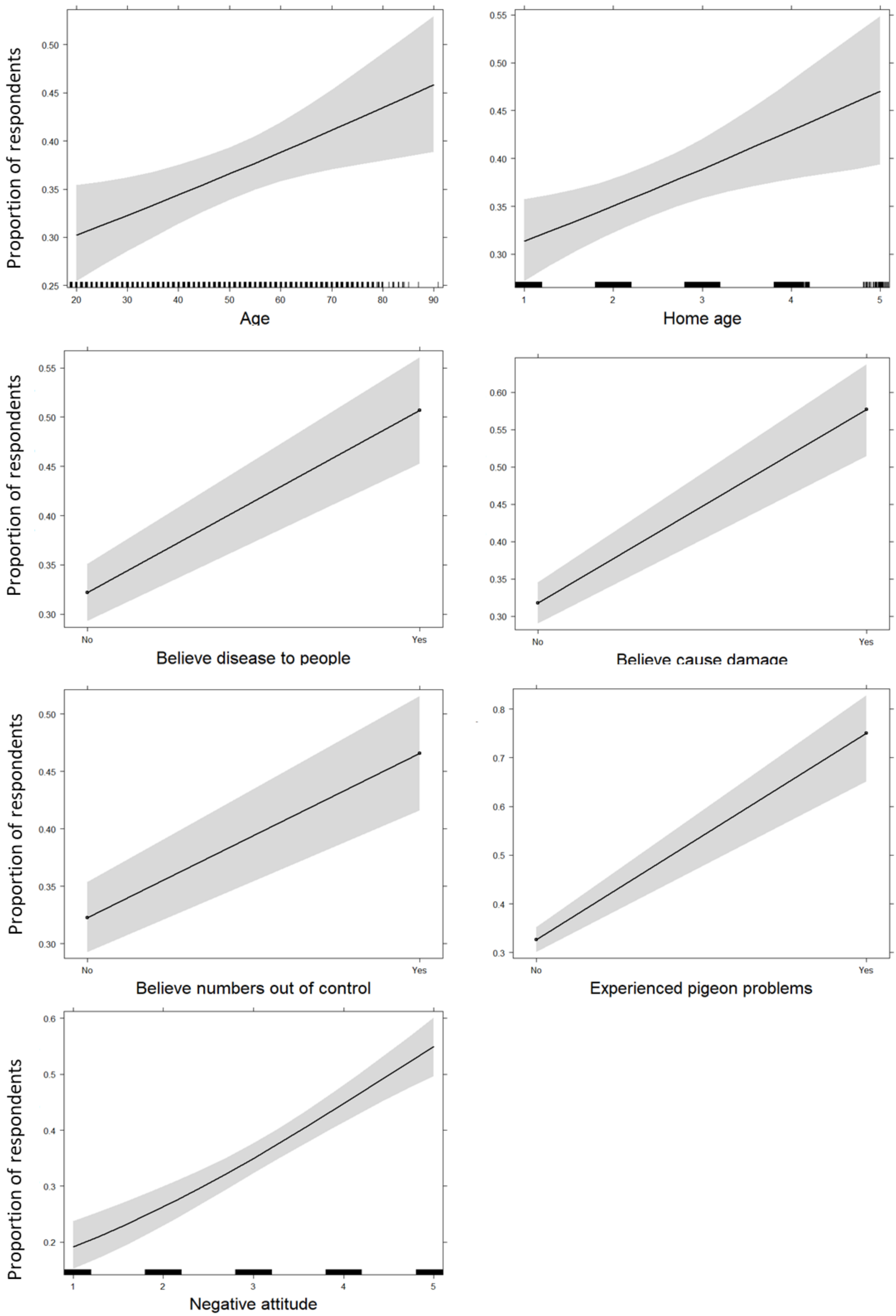

(f) BELIEVE RABBITS CAUSE PROBLEMS GENERALLY

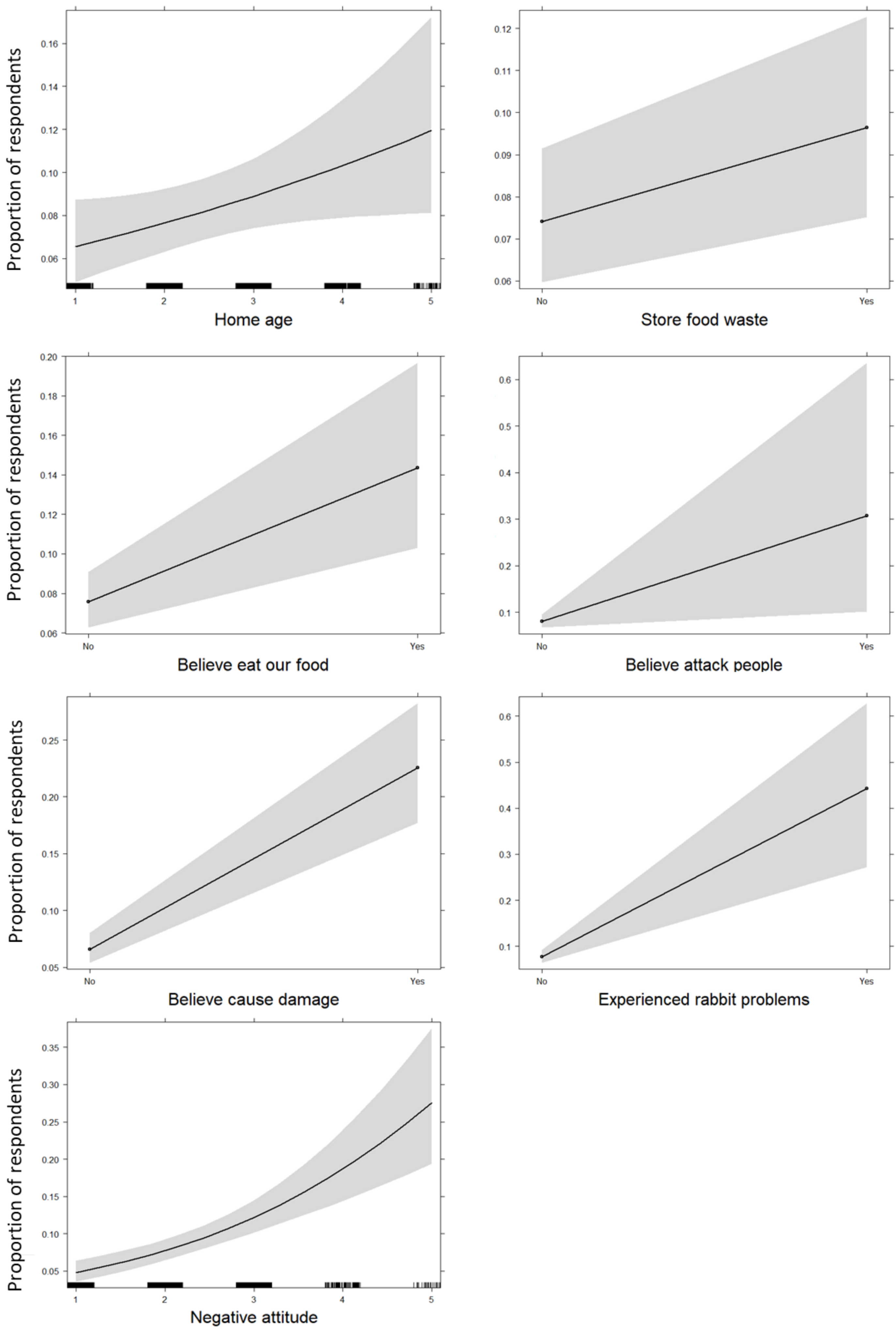

(g) BELIEVE RATS CAUSE PROBLEMS GENERALLY

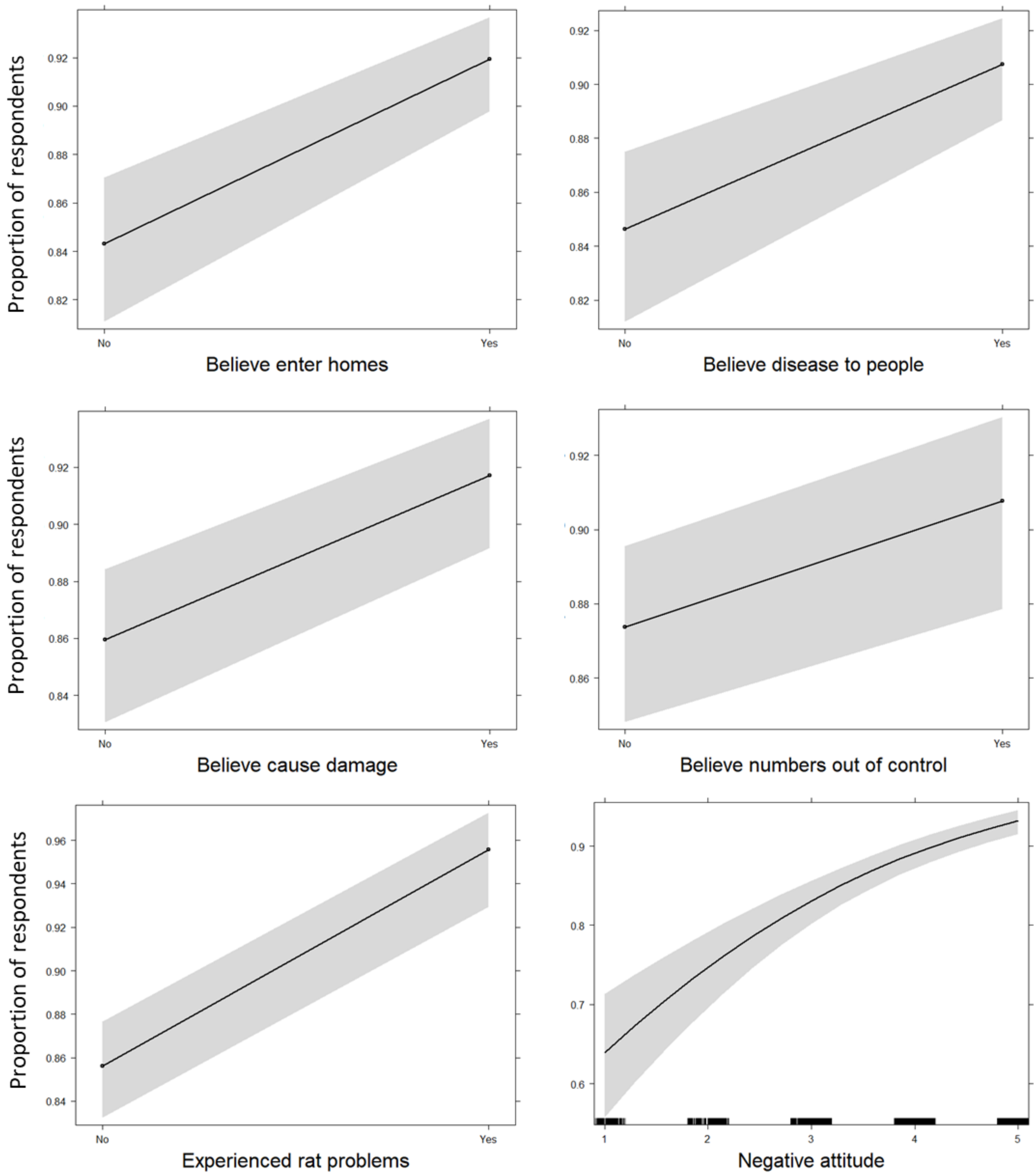

(h) BELIEVE GULLS CAUSE PROBLEMS GENERALLY

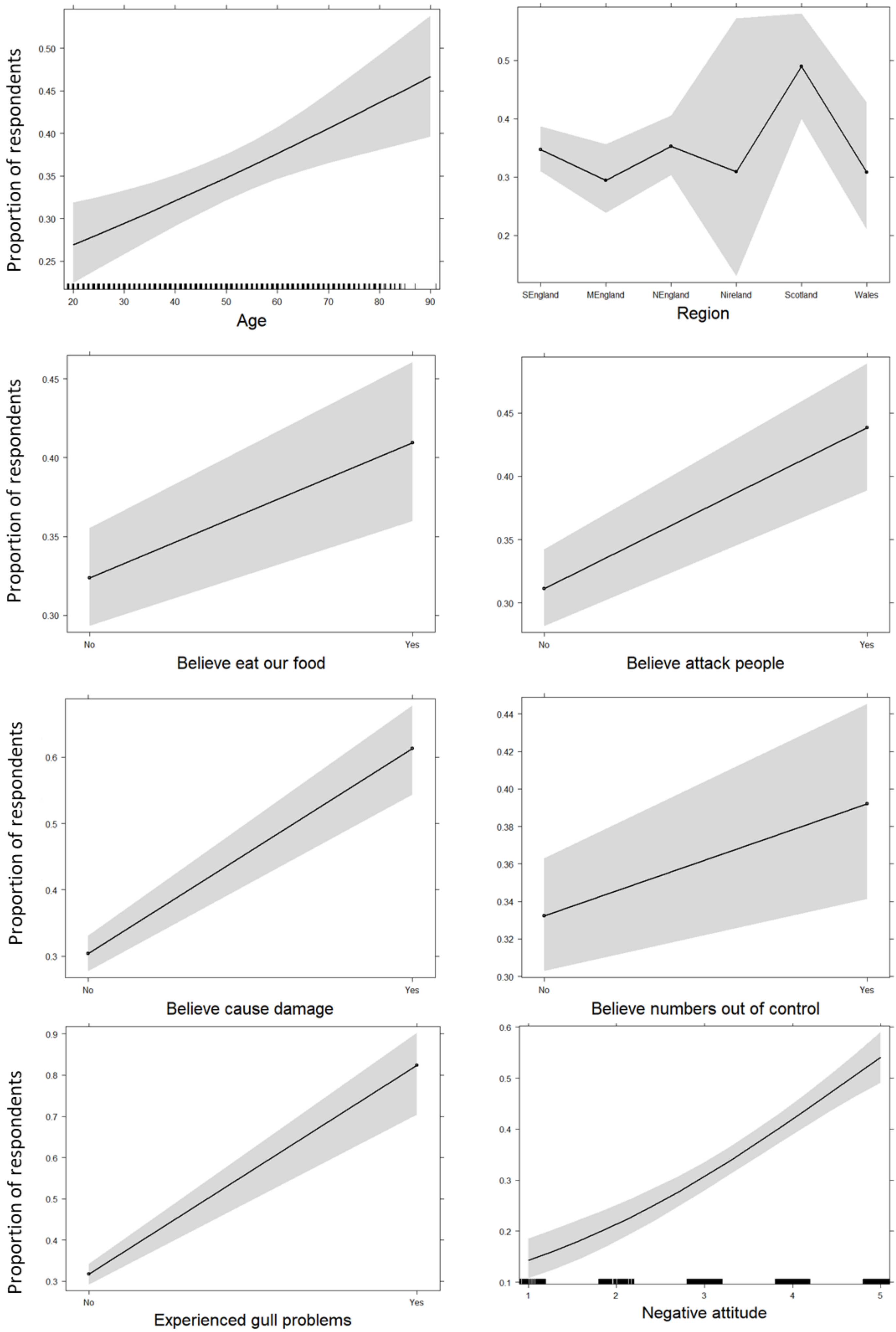

(i) BELIEVE SQUIRRELS CAUSE PROBLEMS GENERALLY

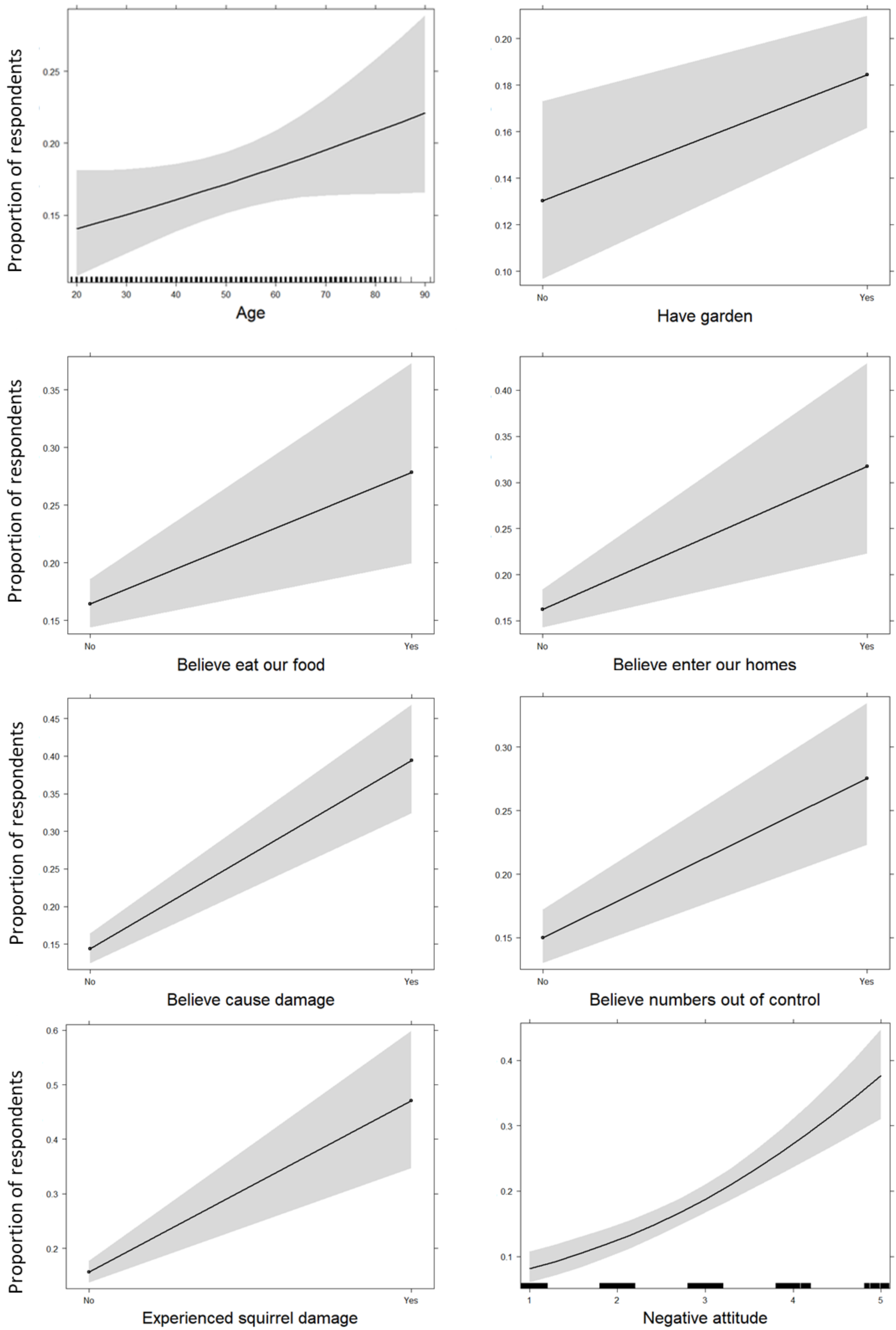

(j) BELIEVE WASPS CAUSE PROBLEMS GENERALLY

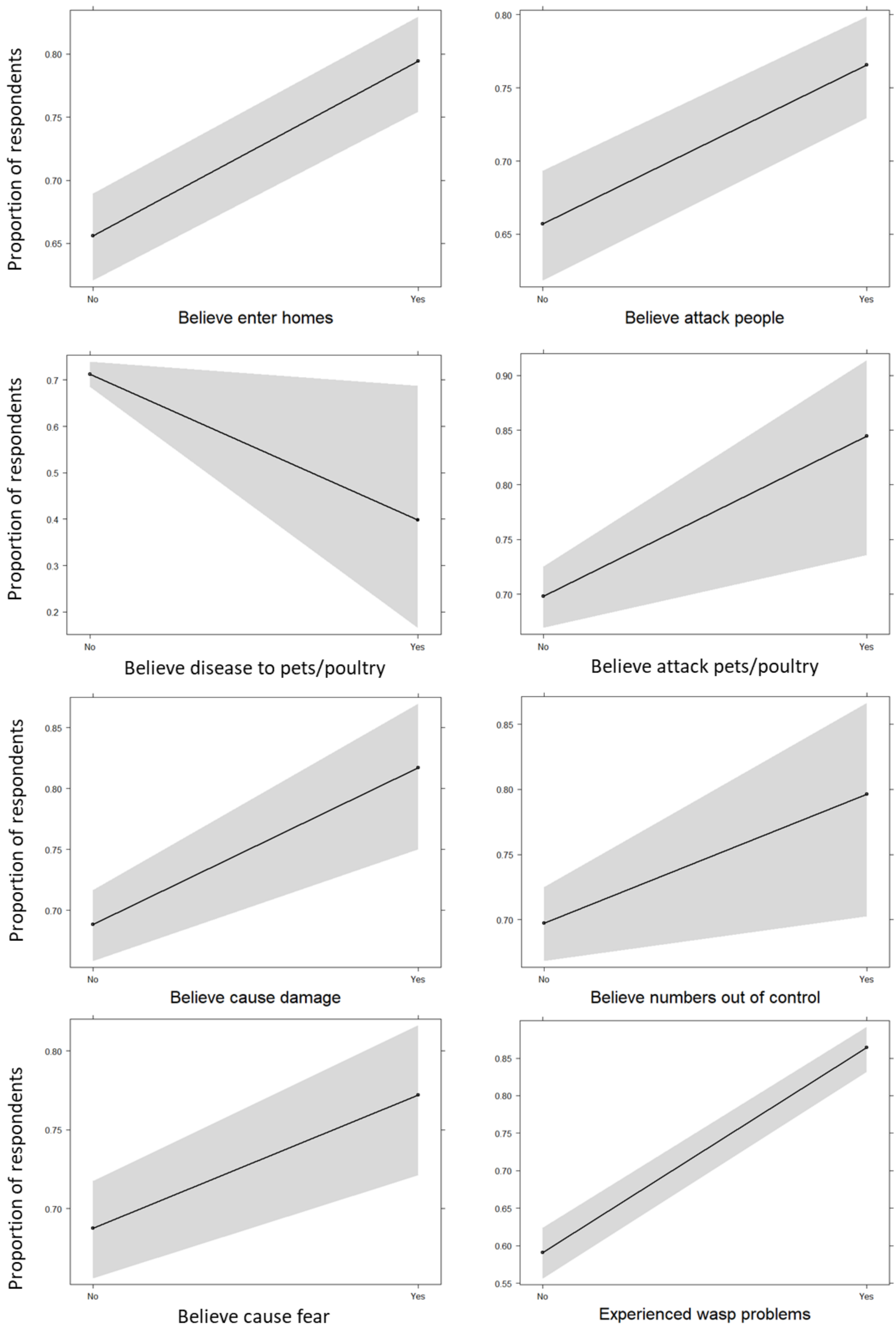

(j) BELIEVE WASPS CAUSE PROBLEMS GENERALLY (CONTINUED)

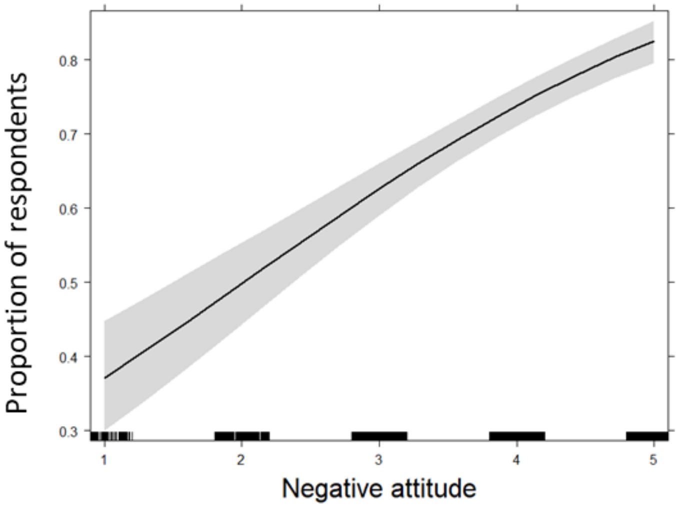

Figure S12. Drivers of respondents' use of negative language ('pest' and/or 'vermin') for each species (marginal effects in logistic regression models), among respondents believing that a particular species causes problems generally: (a) badgers; (b) foxes; (c) moles; (d) mice; (e) pigeons; (f) rabbits; (g) rats; (h) gulls; (i) squirrels; (j) wasps. Blue is not 'pest' or 'vermin', pink = 'pest', green = 'vermin', red = 'pest' and 'vermin'. Confidence Intervals are shown in grey. 'Believe numbers' = Believe numbers are out of control. Home age categories are: 1 = 21st Century (≥2001), 2 = Post World War II (1946-2000), 3 = Pre-World War II (1902-1945), 4 = Victorian (1837-1901), 5 = Pre-Victorian (≤1836). Home type categories are: Semi-detached; Terraced; Flat/maisonette; Detached; Bungalow; Other. Home tenure categories are: Own/part own; Rented private; Social housing; Live with family or friends; Other. Social grade categories are: AB (higher and intermediate managerial, administrative, professional occupations) =1; C1 (supervisory, clerical and junior managerial, administrative, professional occupations) =2; C2 (skilled manual occupations) =3; DE (semi-skilled and unskilled manual occupations, unemployed and lowest grade occupations) =4. Geographical areas are: Southern England, Middle England, Northern England, Northern Ireland, Scotland and Wales.

(a) NEGATIVE LANGUAGE ABOUT BADGERS

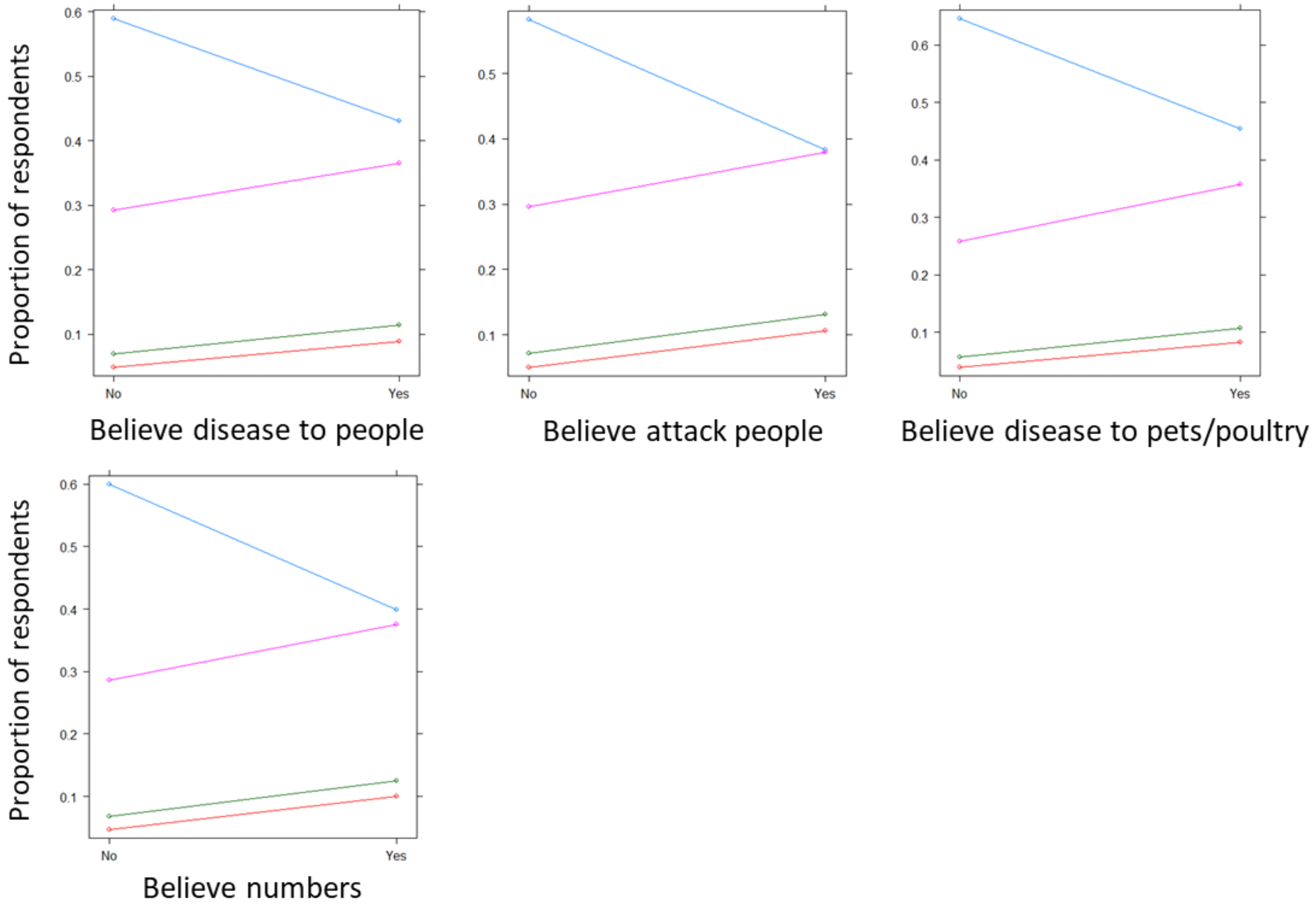

(b) NEGATIVE LANGUAGE ABOUT FOXES

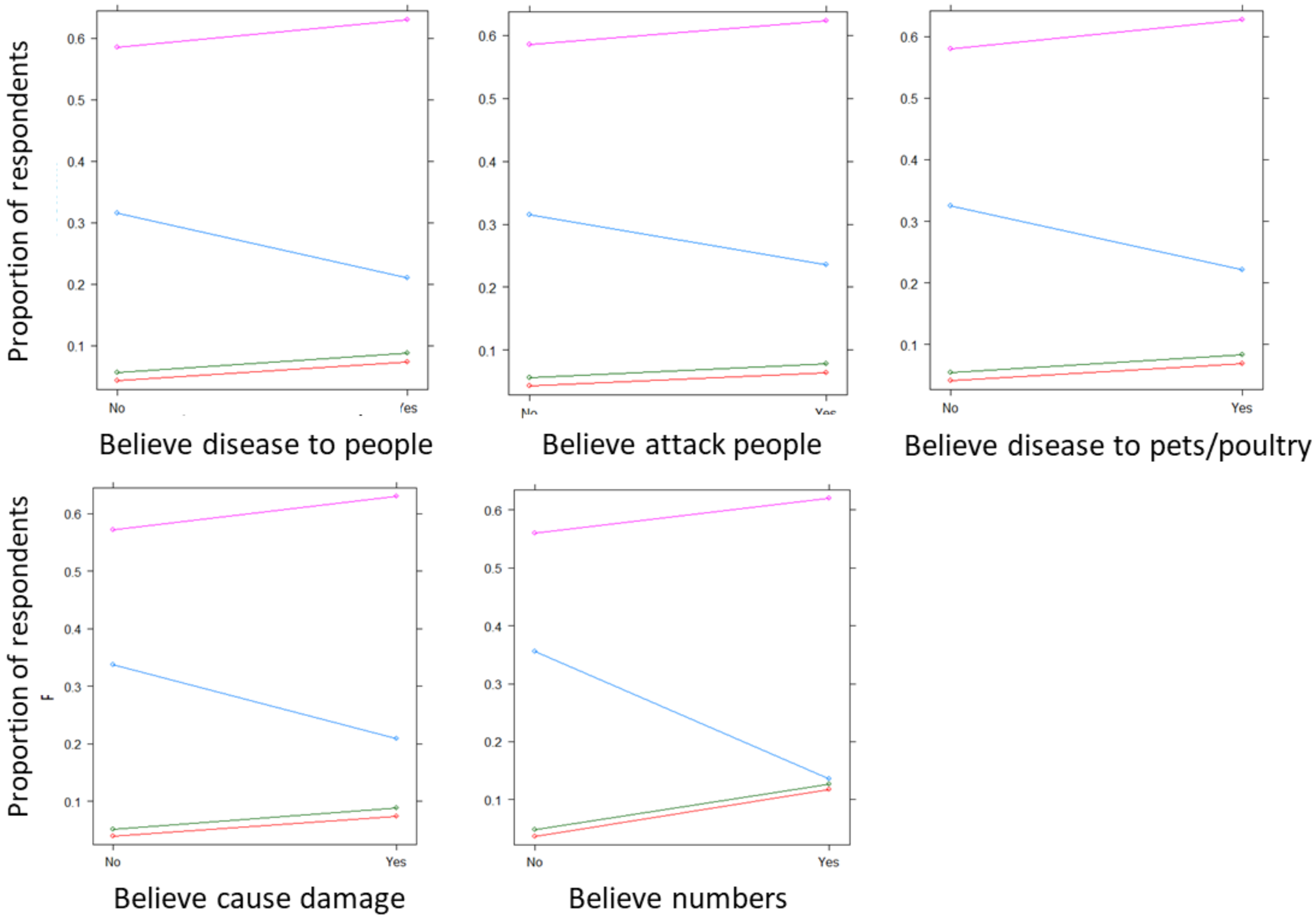

(c) NEGATIVE LANGUAGE ABOUT MOLES

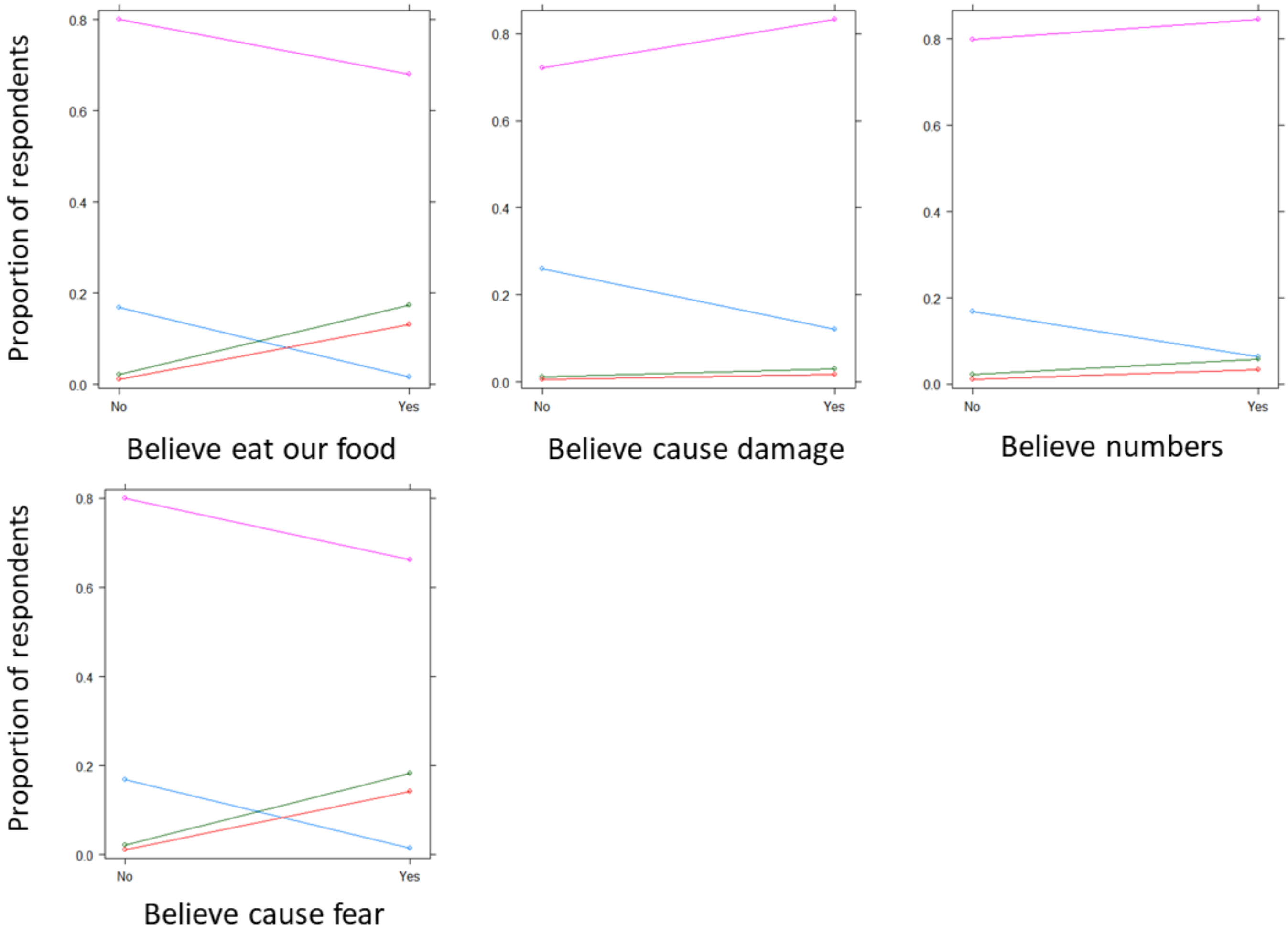

(d) NEGATIVE LANGUAGE ABOUT MICE

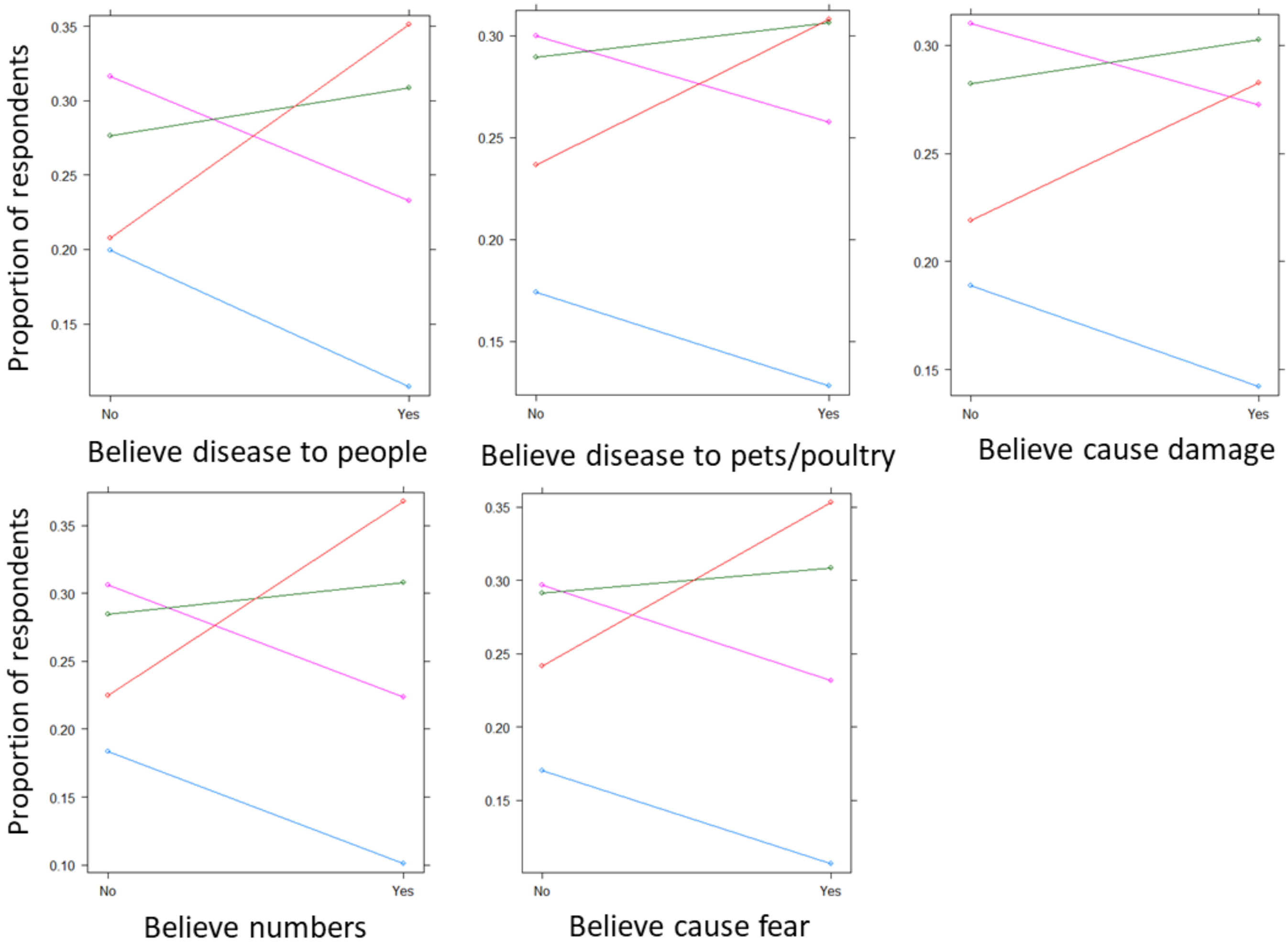

(e) NEGATIVE LANGUAGE ABOUT PIGEONS

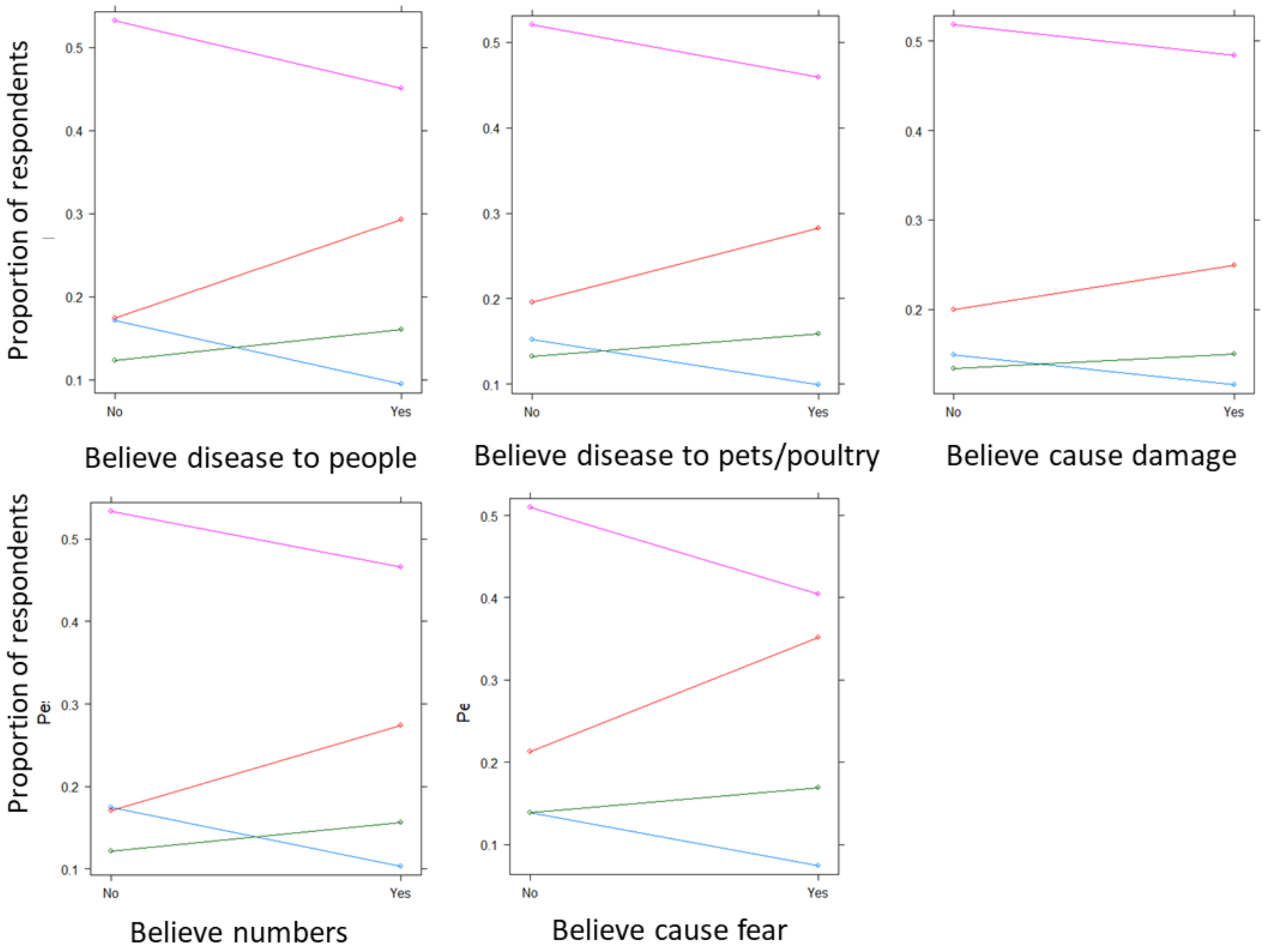

(f) NEGATIVE LANGUAGE ABOUT RABBITS

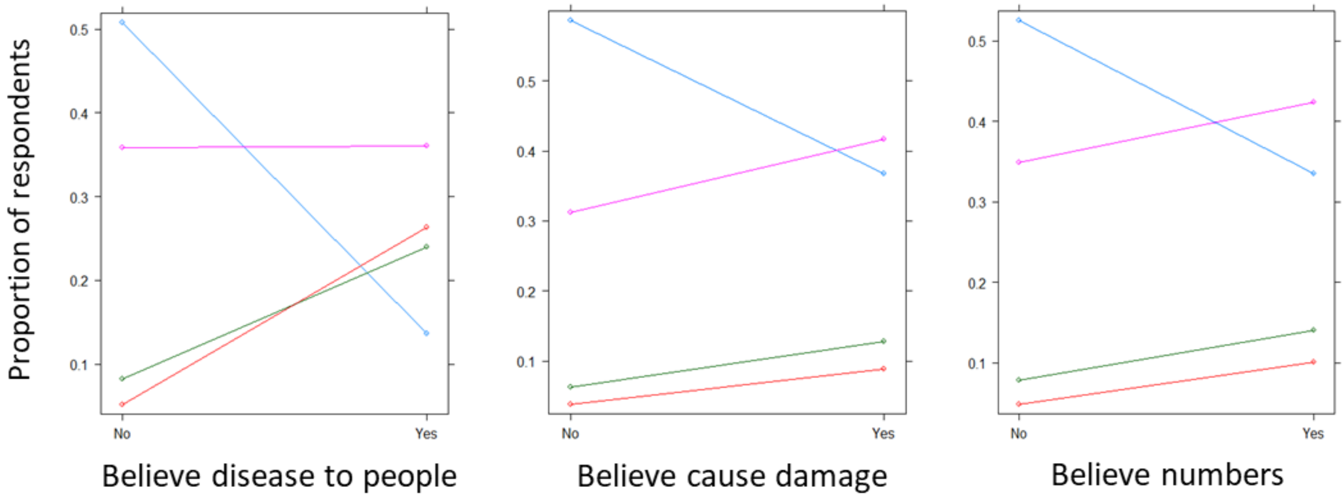

(g) NEGATIVE LANGUAGE ABOUT RATS

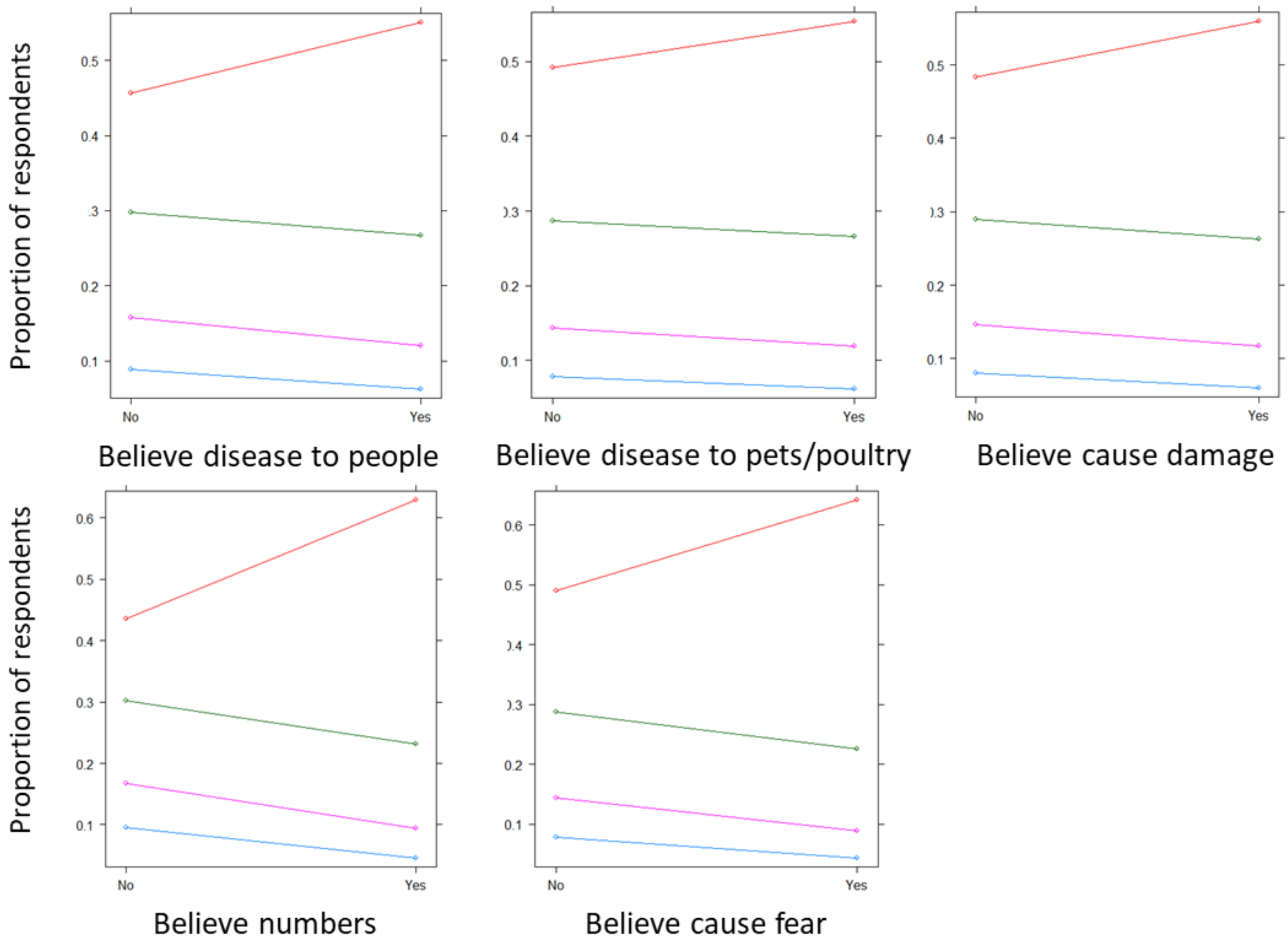

(h) NEGATIVE LANGUAGE ABOUT GULLS

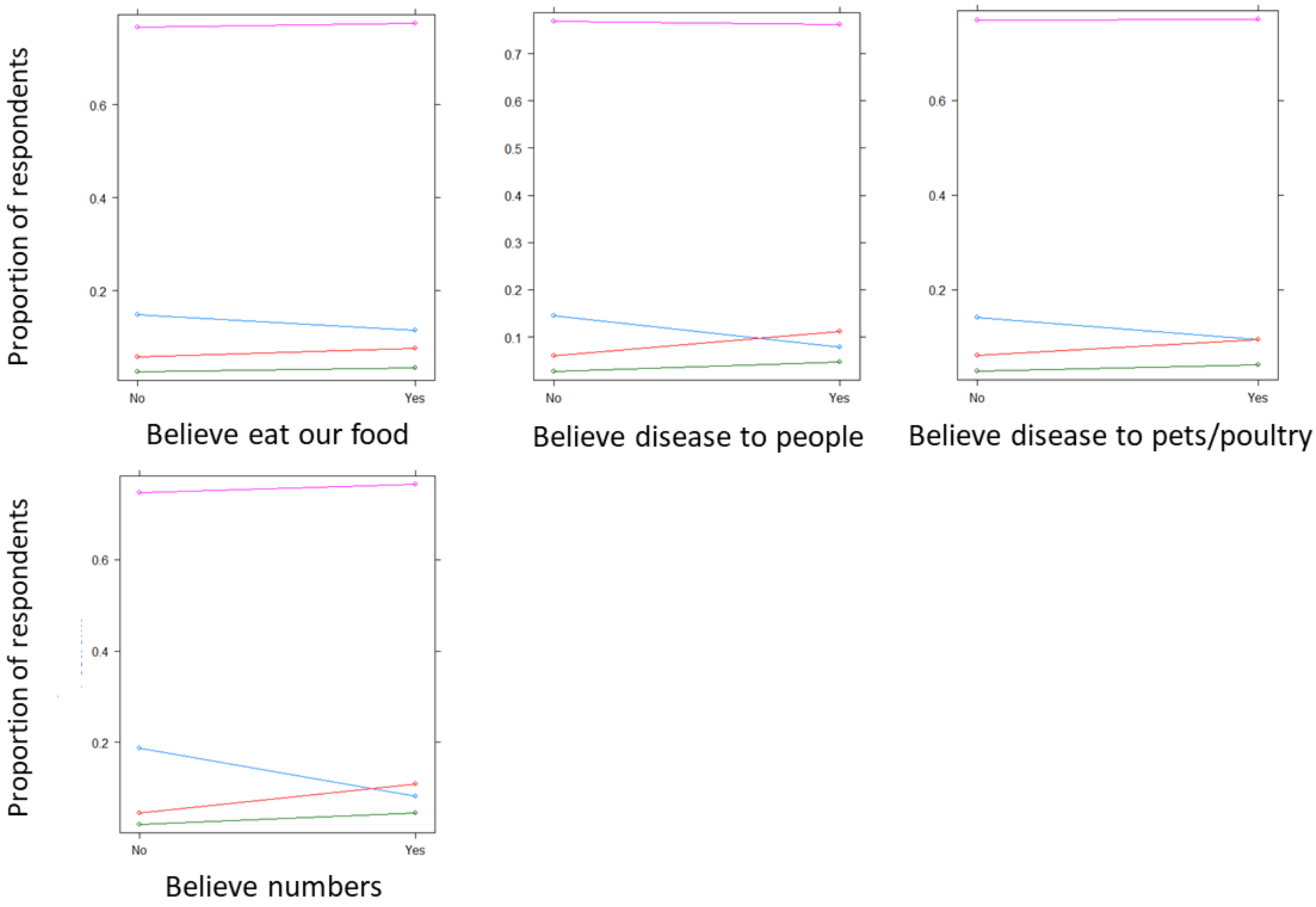

(i) NEGATIVE LANGUAGE ABOUT SQUIRRELS

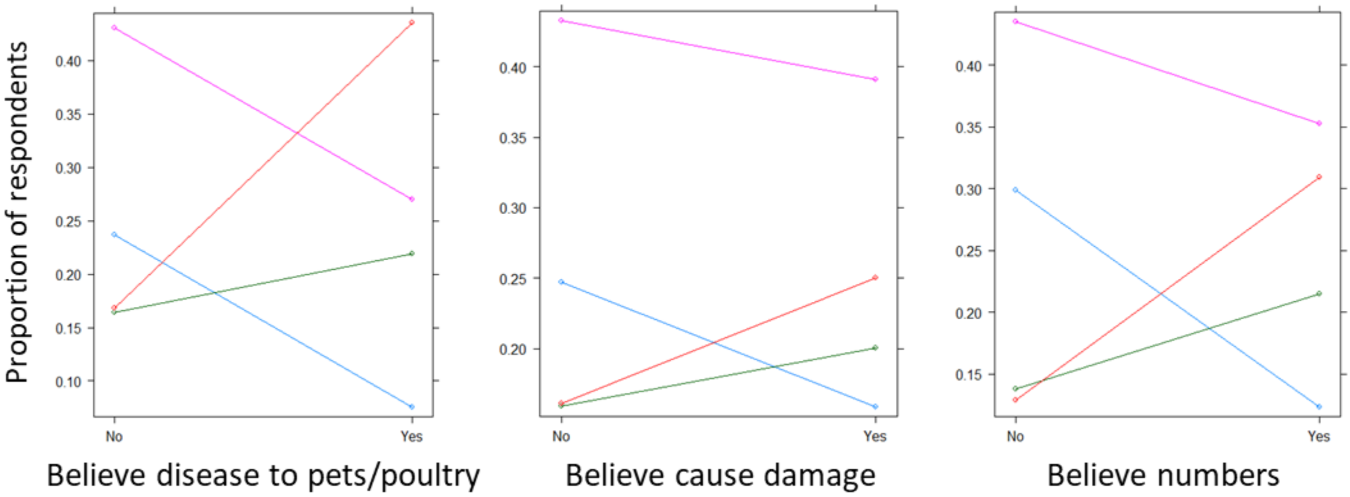

(j) NEGATIVE LANGUAGE ABOUT WASPS

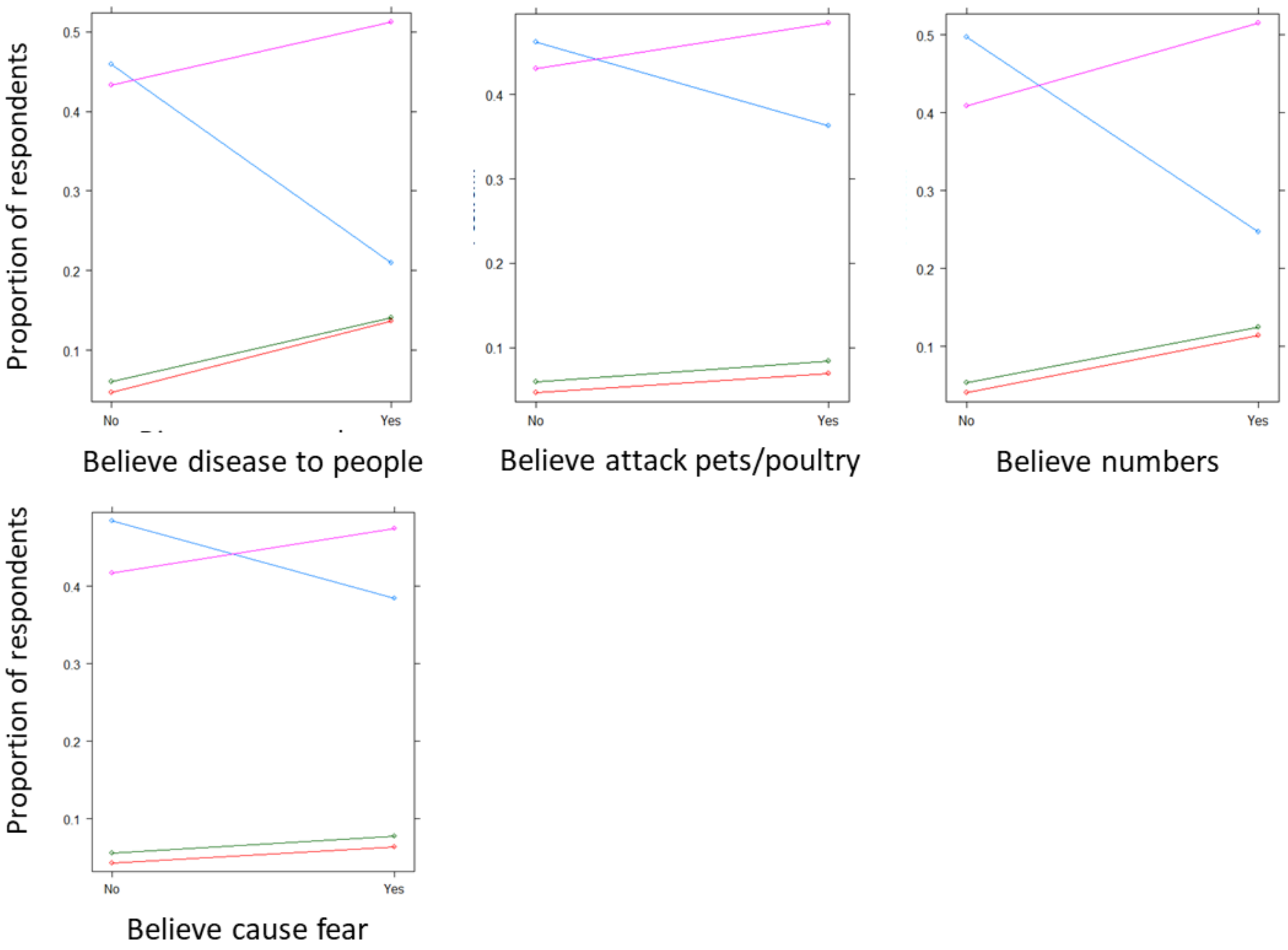

Figure S13. Drivers of respondents' past use of control for each species (marginal effects in logistic regression models), among respondents reporting experience of problems with a particular species: (a) moles; (b) mice; (c) rats; (d) wasps. Confidence Intervals are shown in grey. 'Believe numbers' = Believe numbers are out of control. Home age categories are: 1 = 21st Century (≥2001), 2 = Post World War II (1946-2000), 3 = Pre-World War II (1902-1945), 4 = Victorian (1837-1901), 5 = Pre-Victorian (≤1836). Home type categories are: Semi-detached; Terraced; Flat/maisonette; Detached; Bungalow; Other. Home tenure categories are: Own/part own; Rented private; Social housing; Live with family or friends; Other. Social grade categories are: AB (higher and intermediate managerial, administrative, professional occupations) =1; C1 (supervisory, clerical and junior managerial, administrative, professional occupations) =2; C2 (skilled manual occupations) =3; DE (semi-skilled and unskilled manual occupations, unemployed and lowest grade occupations) =4. Geographical areas are: Southern England, Middle England, Northern England, Northern Ireland, Scotland and Wales.

(a) PAST MOLE CONTROL

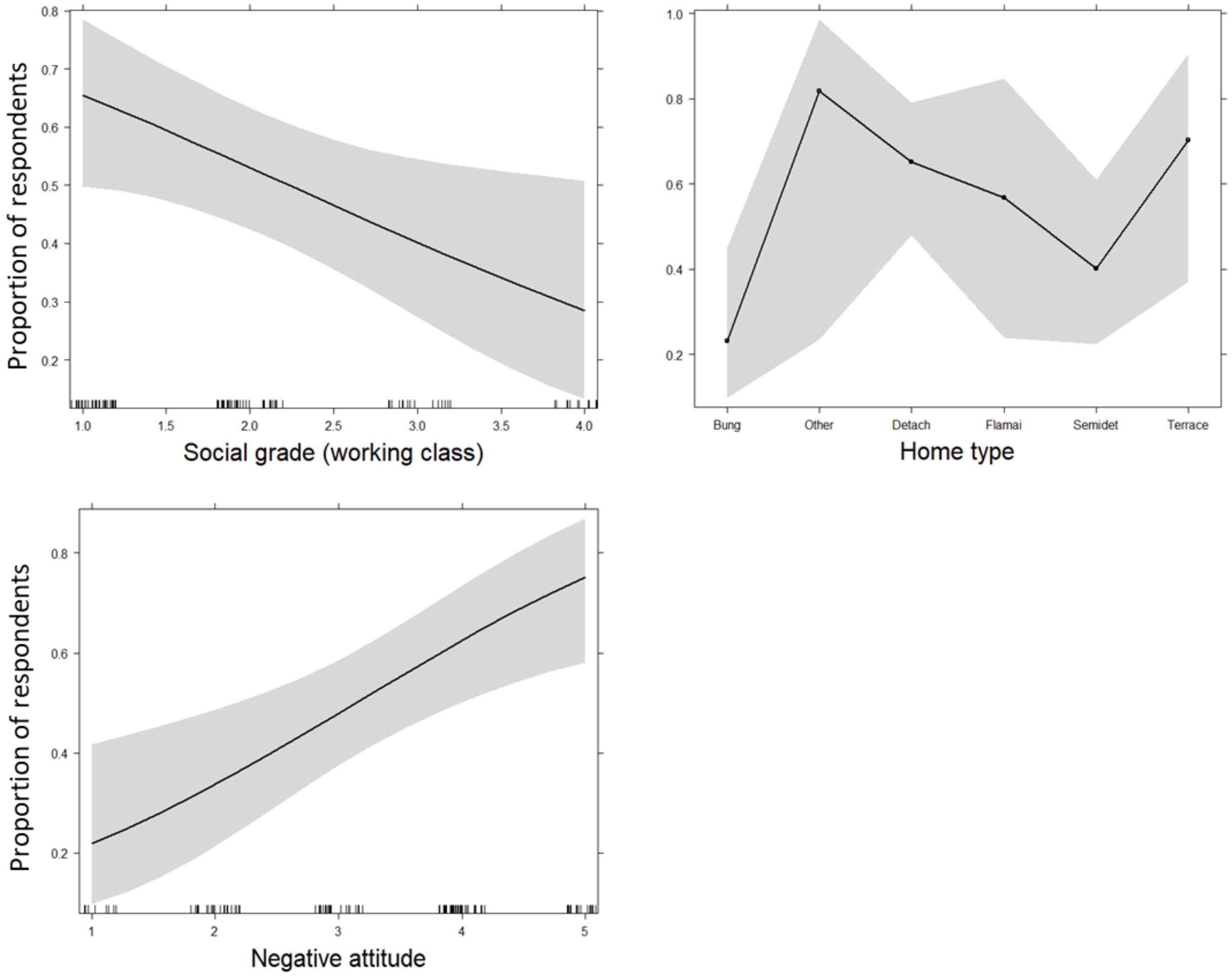

(b) PAST MOUSE CONTROL

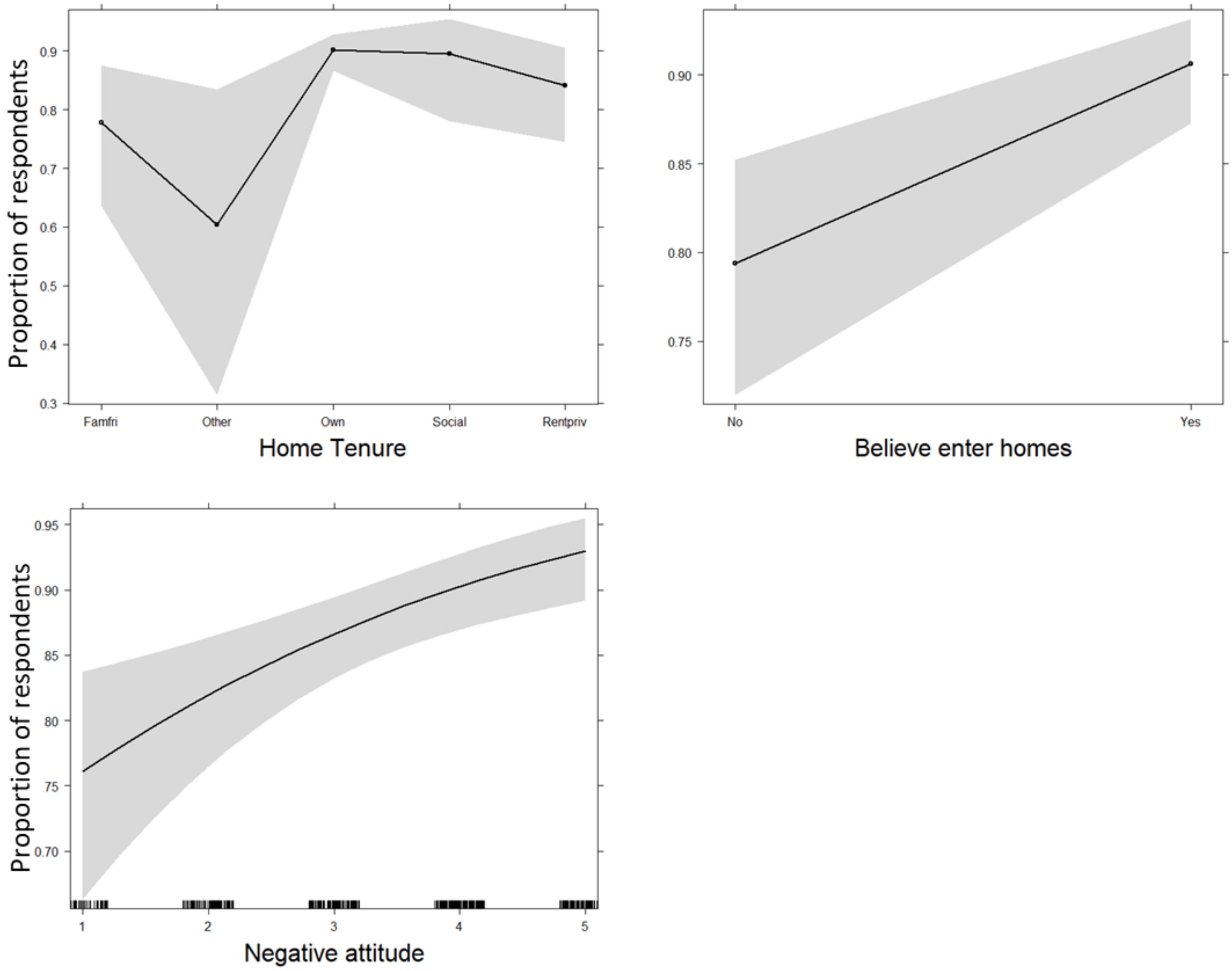

(c) PAST RAT CONTROL

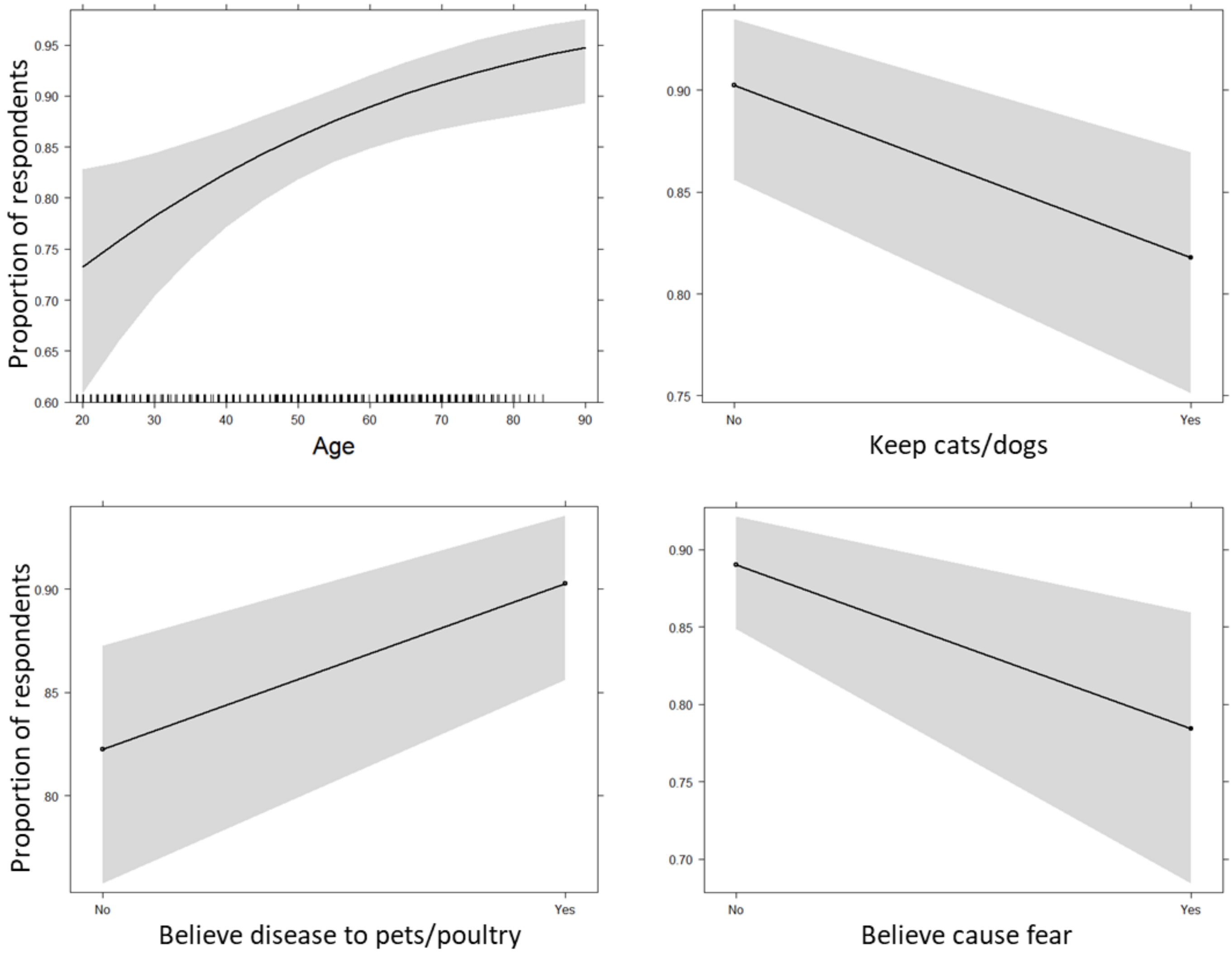

(d) PAST WASP CONTROL

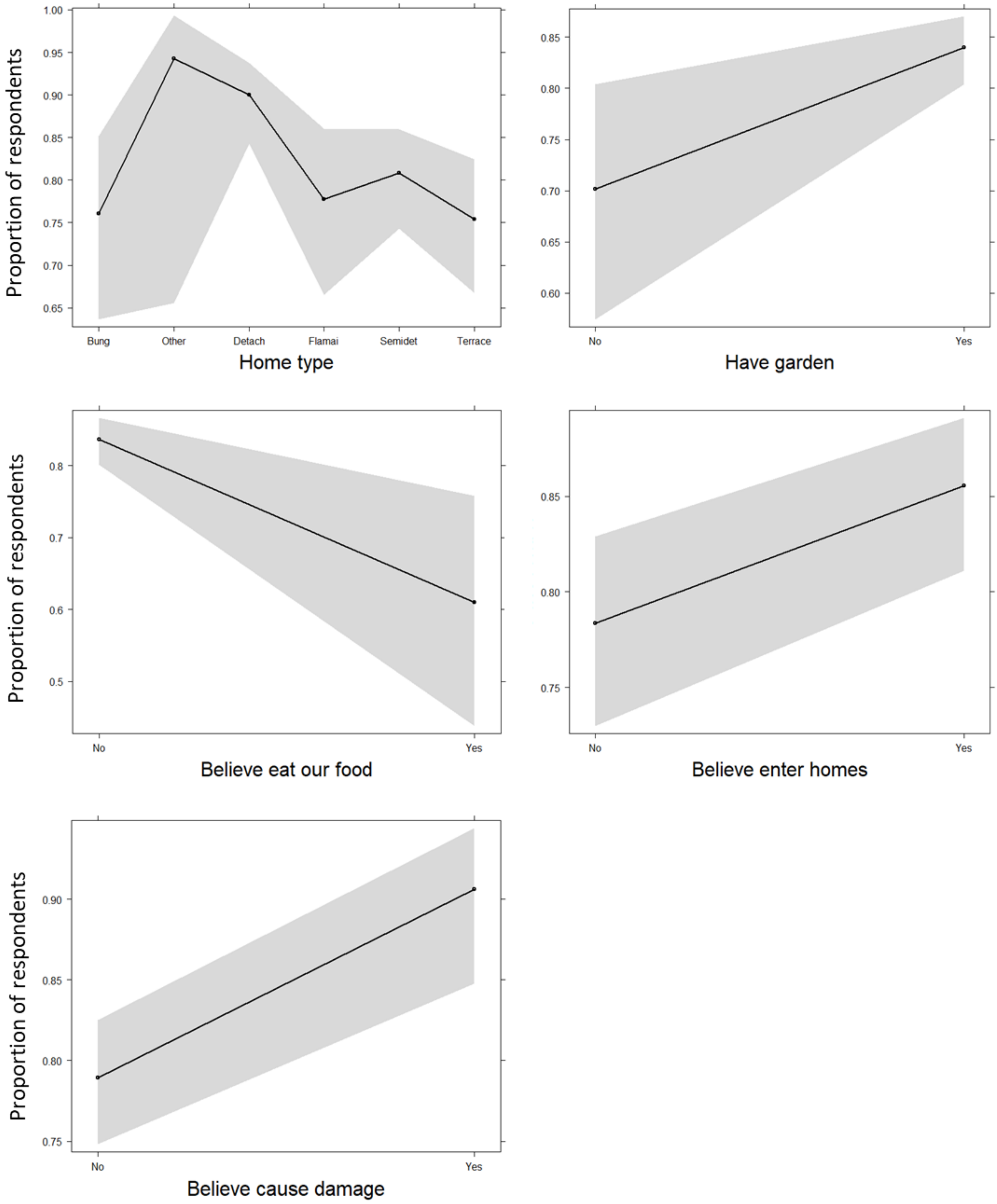

Figure S14. The proportion of councils offering different pest control service types in Middle England (ME), Southern England (SE), Northern England (NE), Scotland (Sc) and Wales (Wa). Red = contracted-out, Khaki = contracted out and referral, Green = In-house, Blue = in-house and contracted-out, Pink = Referral. N = 294.

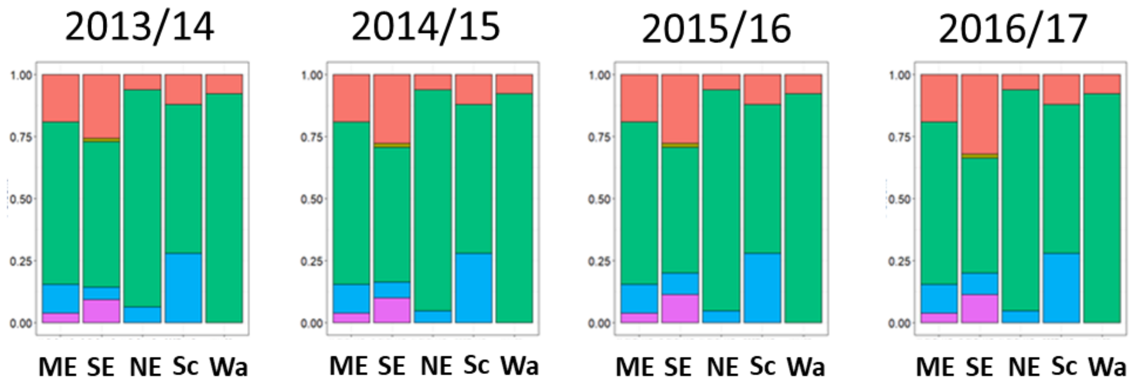

Figure S15. Mean gross expenditure and mean gross revenue per 1,000 households per council associated with providing a public pest control service for 2013/14 (expenditure: n=261, revenue: n=270), 2014/15 (expenditure: n=265, revenue: n=276), 2015/16 (expenditure: n=268, revenue: n=278) and 2016/17 (expenditure: n=264, revenue: n=277). Error bars represent the standard error of the mean.

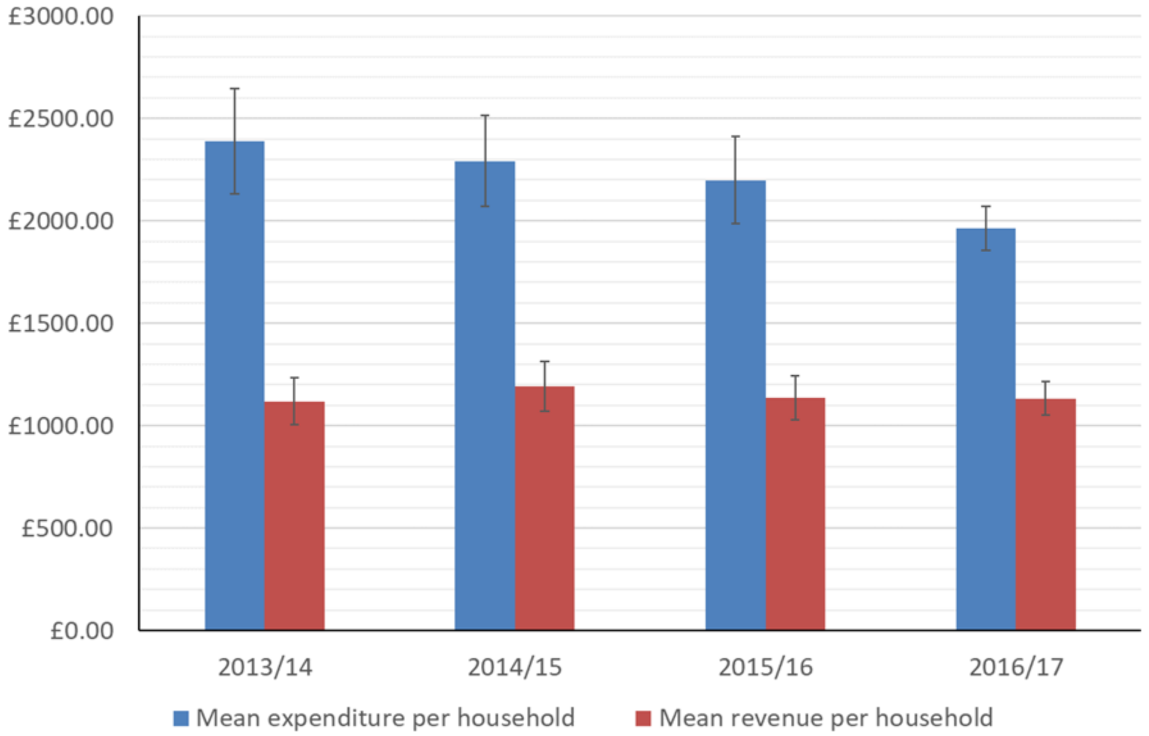

Figure S16: Proportion of UK councils making a net loss and a net gain from public pest control in 2013/14 (n=258), 2014/15 (n=264), 2015/16 (n=267) and 2016/17 (n=263).

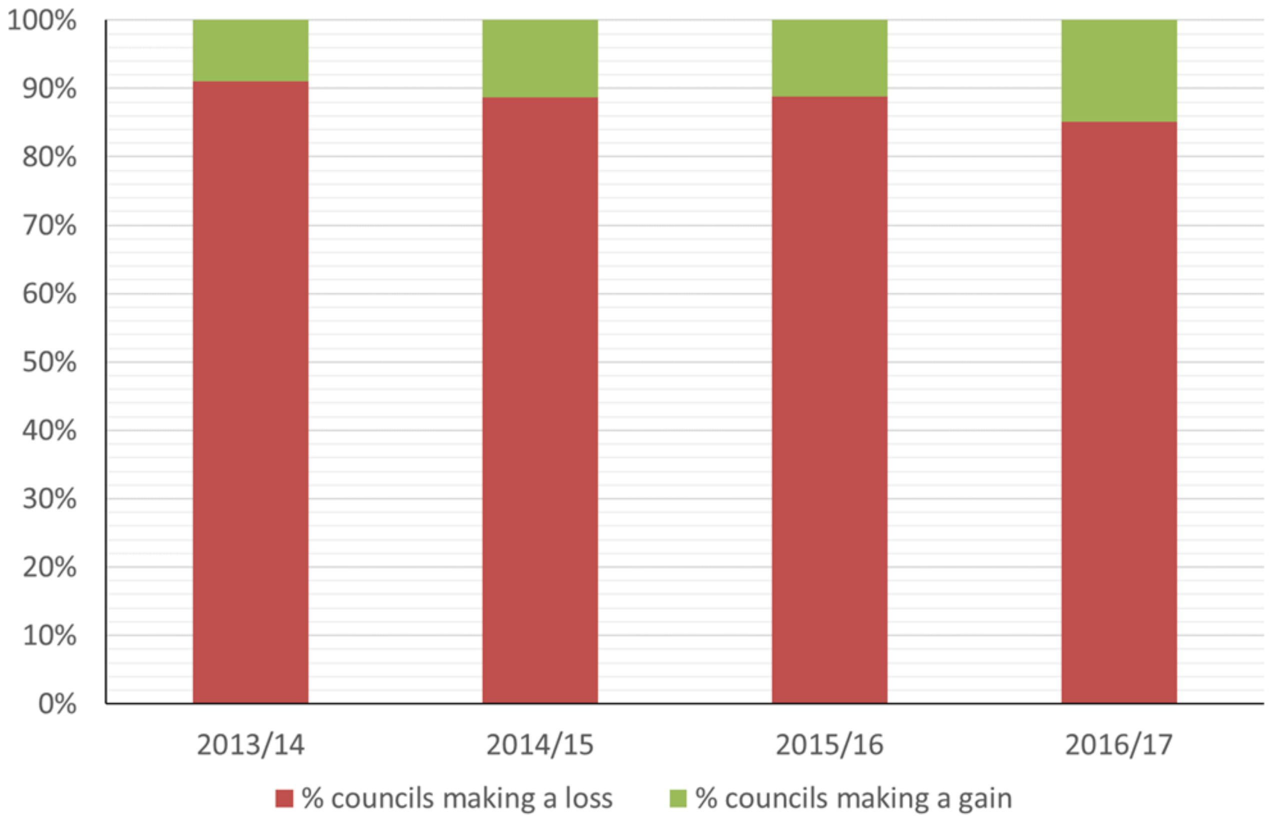

Figure S17: Proportion of councils offering free and paid treatments for each species out of all UK councils providing public pest control in 2017/18 (n=309).

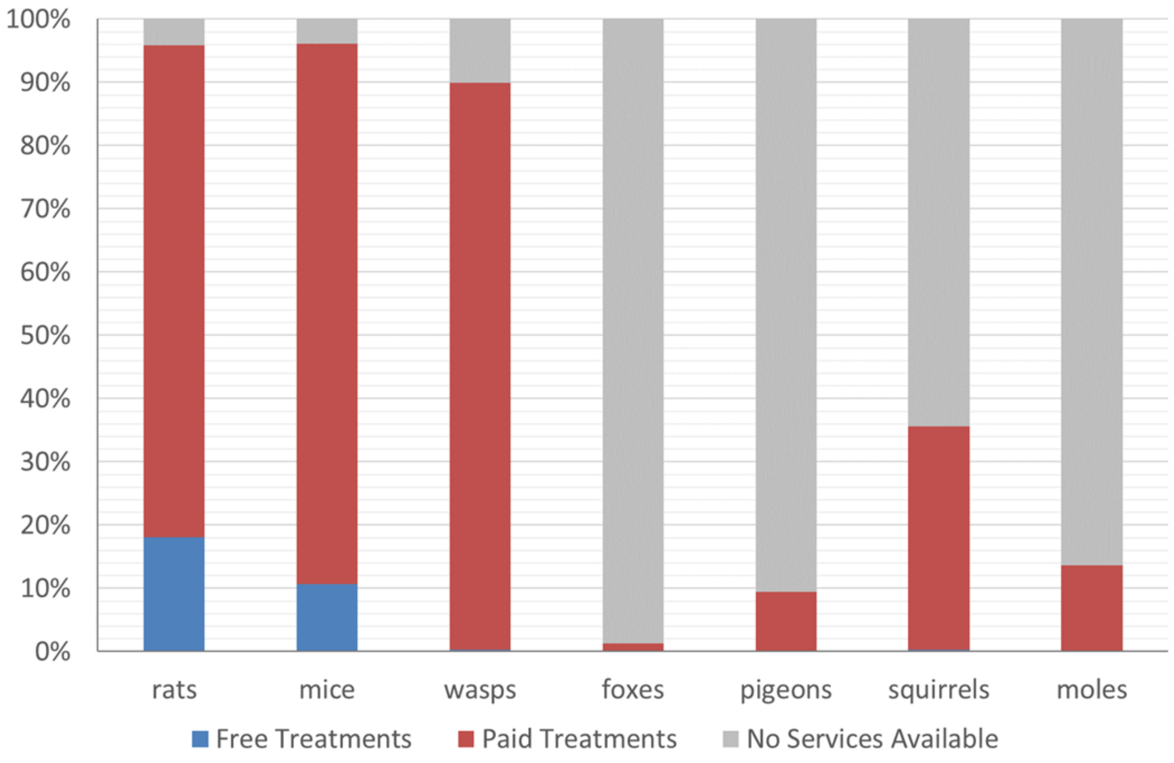

Figure S18: Proportion of councils with (green) and without (red) prevention and deterrence advice for one or more of the selected species, out of all unitary and district councils in East Midlands (n=40), East (n=47), London (n=33), North East (n=12), North West (n=39), Northern Ireland (n=11), South East (n=66), South West (n=37), Scotland (n=32), West Midlands (n=30), Wales (n=22) and Yorkshire and the Humber (n=21).

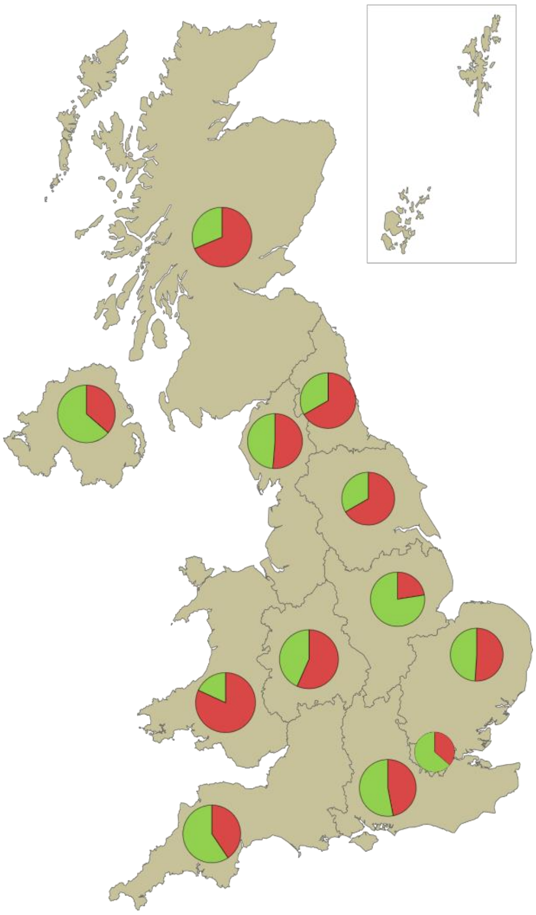

Supplement: Supplementary file 1 [file animals-10-00222-s001.pdf]
